# Efficient Synthesis of α-Branched Purine-Based Acyclic Nucleosides: Scopes and Limitations of the Method

**DOI:** 10.3390/molecules25184307

**Published:** 2020-09-19

**Authors:** Jan Frydrych, Lenka Poštová Slavětínská, Martin Dračínský, Zlatko Janeba

**Affiliations:** Institute of Organic Chemistry and Biochemistry of the Czech Academy of Sciences, Flemingovo nám. 2, CZ-16610 Prague 6, Czech Republic; jan.frydrych@uochb.cas.cz (J.F.); lenka.slavetinska@uochb.cas.cz (L.P.S.); martin.dracinsky@uochb.cas.cz (M.D.)

**Keywords:** acyclonucleosides, hemiaminal ether, purine, multi-component reaction

## Abstract

An efficient route to acylated acyclic nucleosides containing a branched hemiaminal ether moiety is reported via three-component alkylation of *N*-heterocycle (purine nucleobase) with acetal (cyclic or acyclic, variously branched) and anhydride (preferentially acetic anhydride). The procedure employs cheap and easily available acetals, acetic anhydride, and trimethylsilyl trifluoromethanesulfonate (TMSOTf). The multi-component reaction is carried out in acetonitrile at room temperature for 15 min and provides moderate to high yields (up to 88%) of diverse acyclonucleosides branched at the aliphatic side chain. The procedure exhibits a broad substrate scope of *N*-heterocycles and acetals, and, in the case of purine derivatives, also excellent regioselectivity, giving almost exclusively *N*-9 isomers.

## 1. Introduction

**Figure 1:**
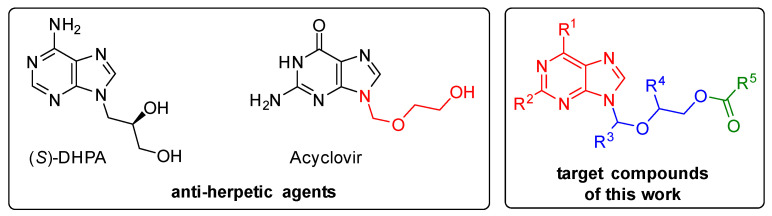
Examples of clinically used acyclic nucleosides and our target compounds.

Structurally modified nucleosides and nucleotides belong to the most studied class of biologically active compounds, especially for their potential antiviral and anticancer properties [1,2,3,4]. Among them, so called acyclic nucleoside and nucleotide analogues have played an important role in the combat with various viral infections [3,5,6]. Acyclic nucleosides (acyclonucleosides), in general, are defined as heterocyclic compounds (usually nucleobase or modified nucleobase) containing one or more functional groups (usually hydroxyls) on the aliphatic side chain. The discovery of acyclovir (Zovirax) [7,8,9] and (*S*)-DHPA [10], the active ingredient of Duviragel (marketed in former Czechoslovakia), as anti-herpetic drugs (Figure 1), sparked the real interest in synthesis and biological evaluation of various types of acyclonucleosides.

**Scheme 1:**
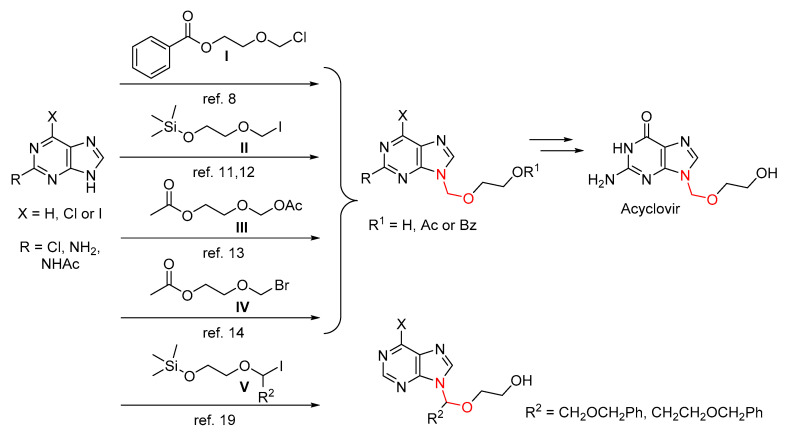
Alkylation of purine nucleobases as the key step in the synthesis of acyclovir.

The key step of the synthesis of acyclovir and its analogues, as the most successful antivirals among acyclonucleosides, consisted of alkylation of properly substituted purine derivative with suitable alkylating agent. This methodology was quite well scrutinized as shown on the example of acyclovir syntheses (Scheme 1). The proper alkylating agents can be conveniently prepared by opening of 1,3-dioxolanes [5]. The original synthesis of acyclovir by Schaeffer et al. [8] started from 2,6-dichloropurine and (2-benzoyloxyethoxy)methyl chloride (**I**, Scheme 1) to give *N*-9 substituted product in a 41% yield.

A more efficient method of acyclovir synthesis was reported by Barrio and coworkers [11,12], when (2-trimethylsilyloxyethoxy)methyl iodide (**II**, Scheme 1), prepared by treatment of 1,3-dioxolane with trimethylsilyl iodide at low temperature, was exploited for alkylation of 2-chloro-6-iodopurine to afford *N*-9 substituted product in a 75% yield. Matsumoto et al. [13] exploited a reaction of diacetylguanine and 2-oxa-1,4-butanediol diacetate (**III**, Scheme 1) in the presence of *p*-toluenesulfonic acid and obtained the *N*-9 substituted product in a 66% yield. The synthesis of purine acyclonucleosides was also studied by Robins and Hatfield [14], where treatment of silylated purines (2,6-dichloropurine and 2-amino-6-chloropurine) with (2-acetoxyethoxy)methyl bromide (**IV**, Scheme 1) in the presence of mercury(II) cyanide afforded desired *N*-9 substituted acyclonucleosides in 89% and 84% yields, respectively. This methodology effectively eliminated formation of *N*-7 isomers that are usually observed during alkylation of purine sodium salts [12].

Acyclovir (acycloguanosine), its derivatives (namely ganciclovir and penciclovir) and their prodrugs (valacyclovir, valganciclovir, and famciclovir, respectively) represent commercially successful anti-herpesvirus drugs [15,16,17,18]. Quite surprisingly, the research of α-branched acyclic nucleoside analogues (i.e., derivatives branched at hemiaminal ether carbon atom C-1′) derived from acyclovir has been somehow neglected and only scarce reports can be found in the literature. Bryant et al. [19] applied the above mentioned methodology [11,12], when treatment of 2-substituted 1,3-dioxolanes with trimethylsilyl iodide at −78 °C afforded the properly branched alkylating agents **V** (Scheme 1), which were further reacted with sodium salt of 6-chloropurine to afford *N*-9 alkylated acyclonucleosides in 50–70% yields.

Other synthetic approaches towards α-branched acyclonucleosides bearing purine nucleobases were based either on an oxidative cleavage of the *cis* vicinal diol group of ribosyl moiety with sodium periodate [20,21], or on a Michael-type addition reaction [22]. These methods, nevertheless, are not suitable for synthesis and biological evaluation of a larger set of acyclonucleosides of the acyclovir type.

Recently, some novel and more efficient synthetic approaches towards purine acyclonucleosides have been reported. *N*-Alkoxyalkylation of nucleobases with cyclic or aliphatic ethers was performed under the catalyzed (CuCl_2_ or *n*-Bu_4_NI) peroxide-promoted (*t*-BuOOH) coupling conditions giving preferentially *N*-9 alkylated purine derivatives in moderate to high yields [23,24]. Another efficient and highly selective method for the synthesis of *N*-9 alkylated purine nucleosides exploited a reaction between purines and alkyl ethers in the presence of (diacetoxyiodo)benzene and iodine [25]. Finally, asymmetric synthesis of purine acyclonucleosides containing a hemiaminal ester moiety was reported via a three-component reaction of purines, aldehydes, and acid anhydrides [26].

α-Branched acyclonucleosides bearing hemiaminal ether moiety represent a great mimic of natural nucleosides. Although quite an impressive progress in synthetic accessibility of such analogues has been done in recent years, a general, efficient, and cheap synthetic methodology towards such compounds is still highly desirable, in order to search for potent antiviral and/or anticancer agents. Herein, we report an attractive and simple multi-component reaction between modified purine (no need for its silylation), anhydride, and acetal in the presence of trimethylsilyl trifluoromethanesulfonate leading to variously branched acyclonucleosides analogues (Figure 1).

## 2. Results and Discussion

Treatment of 6-chloropurine (**1a**) with 2-bromomethyl-1,3-dioxolane (**2a**) and acetic anhydride (**3a**) in a presence of Lewis acid was chosen as a model reaction (Table 1). Since we observed formation of alkylated products for the first time in acetonitrile (MeCN), it was used as a suitable solvent for initial optimization of the reaction conditions. At first, the influence of various Lewis acids and of temperature on the reaction course was studied. Thus, an equimolar mixture of starting compounds **1a**, **2a**, and **3a** with corresponding Lewis acid was stirred in MeCN for 15 min either at room temperature or at 70 °C (analogous treatment at 0 °C gave generally much lower yields, data not shown). The results are summarized in Table 1. The best conversion and regioselectivity (only *N*-9 isomer **4** formed, no *N*-7 isomer **5** observed) was achieved with the use of SnCl_4_ and trimethylsilyl trifluoromethanesulfonate (TMSOTf), up to 83% isolated yields, both at room temperature and at 70 °C. With other Lewis acids used, the conversion was much lower (below 40%) and/or regioselectivity was worse, although *N*-9 isomer **4** always remained the major product. Increased temperature led to significantly increased yields of **4**, compared to room temperature, when FeCl_3_, AlCl_3_, or TiCl_4_ were employed as Lewis acids, but the yields were still lower compared to those with SnCl_4_ or TMSOTf. Furthermore, prolongation of the reaction time had no positive effect on the conversion (data not shown).

Based on the above results (Table 1), the following reaction procedure (general procedure A) was selected for further optimization studies: equimolar (2.0 mmol) mixture of 6-chloropurine (**1a**), 2-bromomethyl-1,3-dioxolane (**2a**), acetic anhydride (**3a**) and TMSOTf in 10 mL of solvent at room temperature for 15 min.

Next, the influence of the solvent was carefully evaluated. The above standard reaction procedure (general procedure A) was carried out in a wide range of solvents (Table 2). In some solvents (DMSO, DMF, NMP, THF, cyclohexane, methanol, pyridine), no reaction was observed, while in other solvents only traces of product (toluene, acetic acid) or low yields of desired product (acetone, DCM) were obtained. High conversion (between 67% and 54%) was observed in nitromethane, EtOAc, and dioxane, but regioselectivity was generally worse and small amounts of undesired *N*-7 isomer **5** (4–6%) were observed or isolated. The best solvent for the glycosylation proved to be originally used MeCN with an 83% yield of compound **4** (Table 1 and Table 2).

**Scheme 2:**
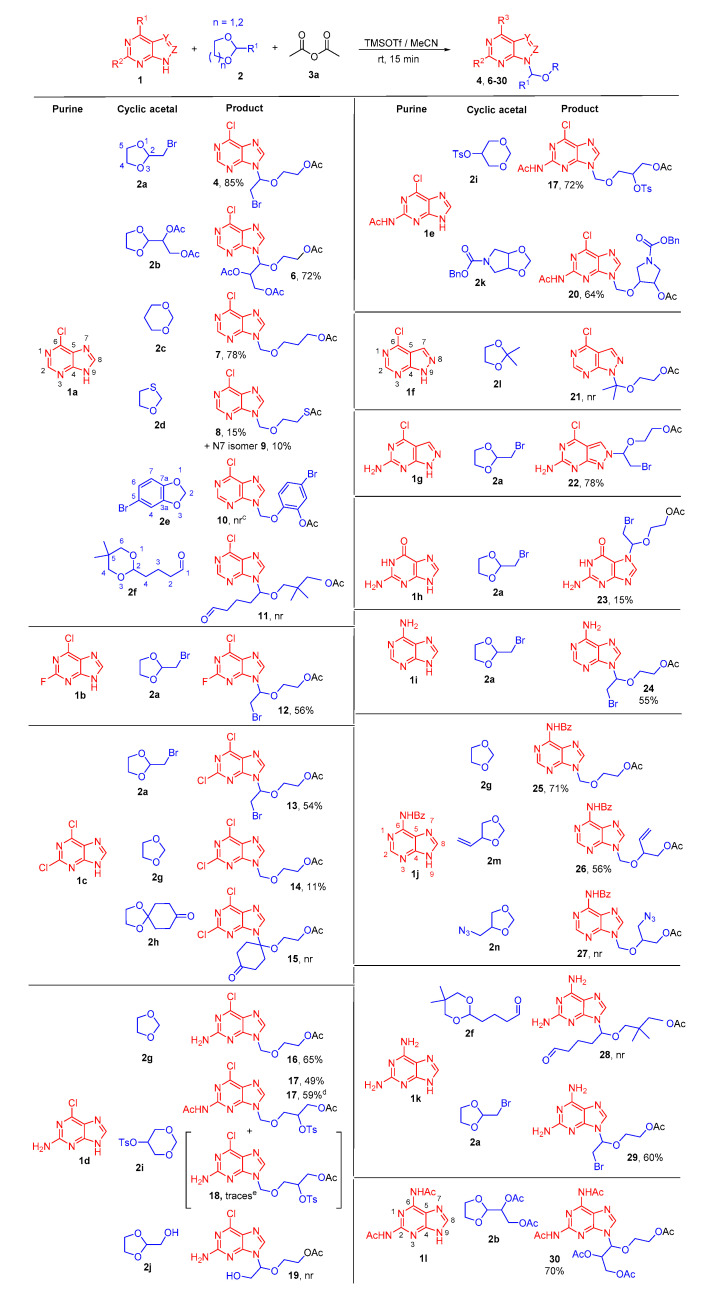
Preparation of acyclonucleosides by multi-component reaction—the scope of purines and cyclic acetals ^a,b^. ^a^ General procedure B. ^b^ Isolated yields. ^c^ nr—no reaction. ^d^ 2.0 equivalent of acetic anhydride used. ^e^ traces—detected by UPLC-MS, not isolated.

The above reaction conditions (general procedure A) developed for the model reaction (Table 1 and Table 2) were slightly optimized into general method B (Scheme 2), where 1.5 equivalent of TMSOTf (instead of 1.0 equivalent) was used, giving comparable or slightly better yield of **4** (85% vs. 83%). It should be noted, that further increase of amount of TMSOTf during the reaction course did not lead to further improvements. With this methodology (general procedure B) in hands, we decided to explore the substrate scope and limitations of this alkylation procedure with regard to *N*-heterocycles, acetals, as well as acid anhydrides and acyl chlorides. 

First, we carried out the reaction with variously substituted purines (including aza/deazapurines, where purine numbering system was used) and with a wide range of cyclic acetals (Scheme 2). Most starting materials were commercially available, some acetals, however, had to be prepared (see the experimental section). It was found, that most of the modified purines were good substrates and afforded the expected products in good to moderate yields, especially with certain type of acetals. For example, when 2-bromomethyl-1,3-dioxolane (**2a**) was used as the reaction partner, all purine analogues used gave the corresponding products in 54–85% yields, with the exception of unprotected guanine (**1h**) when only undesired *N*-7 isomer **23** was obtained in a 15% yield. Moreover, a variety of cyclic acetals, both 5-membered 1,3-dioxolanes (**2a**, **2b**, **2g**, **2k**, and **2m**) and 6-membered 1,3-dioxanes (**2c** and **2i**), afforded the desired products (yields in the range of 11–85%). Nevertheless, when the acetal used contained an aldehyde group (**2f**), a free hydroxyl (**2j**) or an azido group (**2n**), no reaction was observed. Furthermore, the reaction did not take place when 1,3-dioxolanes disubstituted at the acetal carbon atom (compounds **2h** and **2l**) were used as the reaction partners.

In general, desired regioselectivity of the synthetic procedure was great, affording almost exclusively *N*-9 isomers, with two exceptions: treatment of 2-amino-6-chloro-7-deaza-8-azapurine (**1g**) with 2-bromomethyl-1,3-dioxolane (**2a**) gave *N*-8 isomer **22** (78%) and already mentioned reaction of guanine (**1h**) with **2a** afforded *N*-7 isomer **23** (15%). It should be also noted, that isolation of adenine derivative **24** had to be modified since the extraction into chloroform did not work in this case (see the experimental part).

The studied three-component reaction leading to acetylated acyclonucleosides (Scheme 2) proceeded usually with good to high yields and both with five- and six-membered, variously substituted acetals. In several cases, the reaction did not proceed at all or with lower yields. Treatment of 6-chloropurine (**1a**) with 1,3-oxathiolane (**2d**) afforded both *N*-9 isomer **8** (15%) and *N*-7 isomer **9** (10%) in low yields. The alkylation did not proceed with aromatic 1,3-dioxolane **2e** nor with 2,2-disubstituted 1,3-dioxolane **2l** (a compound with disubstituted acetal carbon).

When 2-amino-6-chloropurine (**1d**) was treated with equimolar amount of acetic anhydride (general procedure B), desired product **18** was observed in traces only (UPLC-MS Acquity Waters, USA, H-Class Core System) and the bis-acetylated product **17** was isolated as the major product (49% yield). Interestingly, this was the only case when the exocyclic amino group underwent acetylation during the reaction and up to now, we have no good explanation for this observation. In order to increase the yield, 2 equivalents of acetic anhydride were used next (under otherwise identical conditions), and compound **17** was obtained in a 59% yield. On the other hand, compound **17** was prepared in a 72% yield, when the reaction started directly from *N*-2 acetylated 2-amino-6-chloropurine **1e** instead of **1d**.

Quite a surprise for us was the reaction with carbamate **2k**. Carbamates are, in general, relatively unstable under acidic conditions. However, treatment of carbamate **2k** with *N*-2 acetylated 2-amino-6-chloropurine **1e** gave desired product **20** in a 64% yield.

**Scheme 3:**
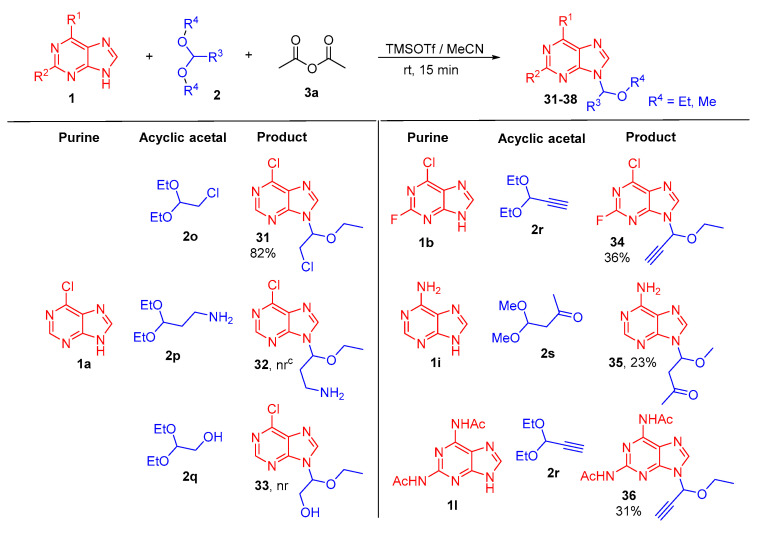
Preparation of acyclonucleosides by multi-component reaction—the scope of purines and acyclic acetals ^a,b^. ^a^ General procedure B. ^b^ Isolated yields. ^c^ nr—no reaction.

Next, the developed alkoxyalkylation methodology (general procedure B) was carried out with selected purines and with various acyclic acetals (Scheme 3). Depending on the starting materials, the reactions afforded products in low to high yields (23–82%), with the exception of acetals containing either unprotected amino (compound **2p**) or hydroxyl (compound **2q**) group, where no reaction was observed.

As shown before, the presence of double bond in acetal **2m** (Scheme 2) was tolerated under the reaction conditions. We were subsequently interested in using acetal with a triple bond. Thus, treatment of purines **1b** and **1l** (Scheme 3) with 3,3-diethoxy-1-propyne (**2r**), diethyl acetal of propynal, afforded desired products **34** (36%) and **36** (31%), respectively, in acceptable yields. Furthermore, ketone containing acetal **2s** was tolerated as well, and product **35** was obtained in a 23% yield.

**Scheme 4:**
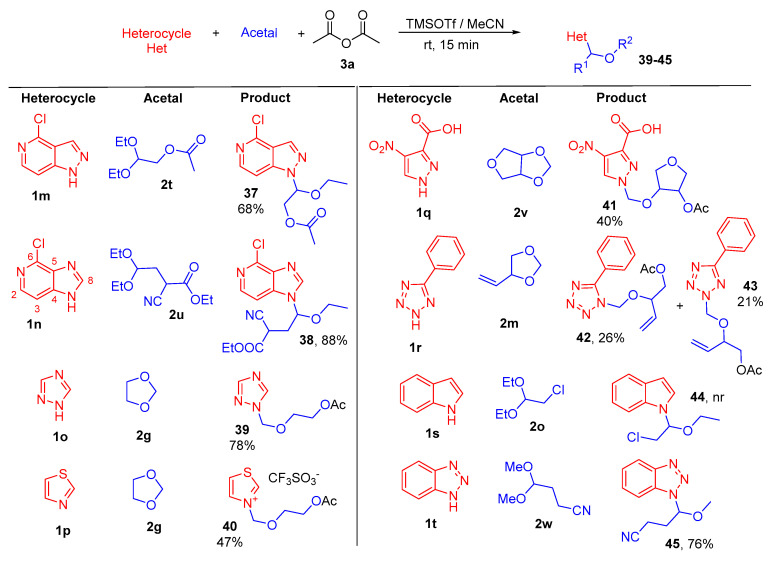
Preparation of acyclonucleosides by multi-component reaction—the scope of nitrogen heterocycles (other than purines) and cyclic/acyclic acetals ^a,b,c^. ^a^ General procedure B. ^b^ Isolated yields. ^c^ nr—no reaction.

In order to extend the substrate scope of the studied synthetic procedure, reactions of various *N*-heterocycles with selected cyclic and acyclic acetals were carried out next (Scheme 4). The reactions afforded acceptable to high yields (21–88%) of target compounds, no reaction was observed only in the case when 1H-indole (**1s**) and acetal **2o** were used as reaction partners. However, when the hydroxyl group of acetal **2q** (Scheme 3) was acetylated, as in acetal **2t** (Scheme 4), desired alkylated product **37** was isolated in a 68% yield. The best yield (88% of **38** as inseparable mixture of diastereoisomers) was obtained during the reaction of 3-deazapurine derivative **1n** with racemic acetal **2u**, which contains both nitrile and ester groups, demonstrating that these functional groups were well-tolerated.

The regioselectivity was usually great, as demonstrated by formation of products **39** (1,2,4-triazole derivative), **41** (pyrazole derivative) and **45** (benzotriazole derivative). The only exception was the reaction of tetrazole **1r** with acetal **2m**, where regioisomers **42** (26%) and **43** (21%) were isolated in almost equimolar ratio.

Furthermore, the reaction of compound **1q** demonstrated that nitro group and even unprotected carboxylic acid are tolerated during the procedure and product **41** was isolated in a 40% yield. In case of thiazolium trifluoromethanesulfonate **40**, which is highly soluble in water, the extraction had to be replaced by the purification on C18-silica gel column. Nevertheless, it can be concluded that described procedure can be applied to a large variety of *N*-heterocyclic compounds.

**Scheme 5:**
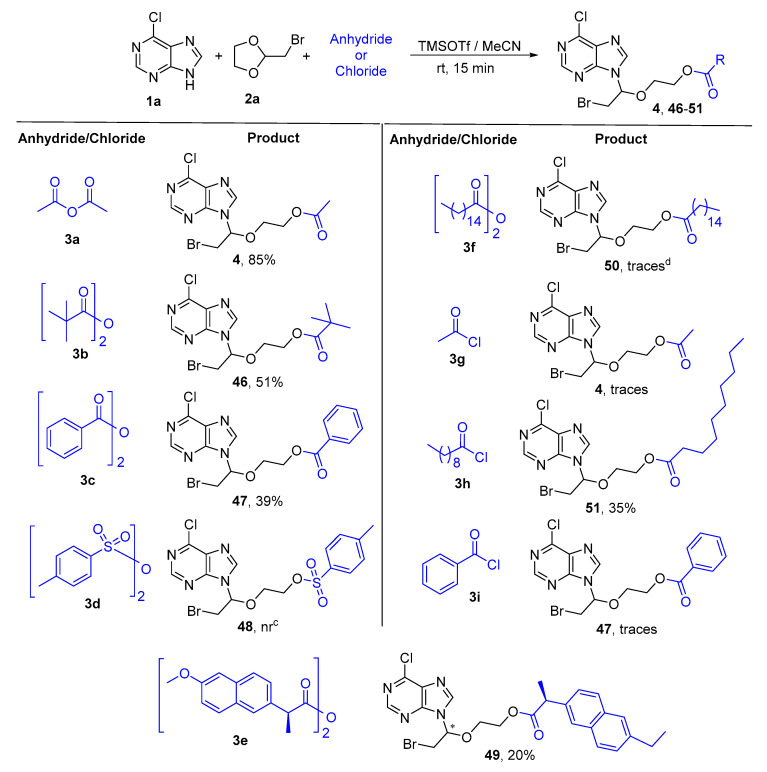
Preparation of acyclonucleosides by multi-component reaction—the scope of carboxyl acid anhydrides and acyl chlorides ^a,b^. ^a^ General procedure B. ^b^ Isolated yields. ^c^ nr—no reaction. ^d^ traces—detected by UPLC-MS, not isolated.

The last goal of the study was to test whether different anhydrides of carboxylic acids or even acyl chlorides could be exploited for such methodology (Scheme 5). For this purpose, our model reaction of 6-chloropurine (**1a**) with 2-bromomethyl-1,3-dioxolane (**2a**) under standard reaction conditions (general procedure B) was chosen, where acetic hydride (**3a**) was replaced by other anhydrides or acyl chlorides.

When pivalic anhydride (**3b**) or benzoic anhydride (**3c**) were used, the corresponding acylated acyclonucleosides **46** (51%) and **47** (39%), respectively, were obtained in satisfactory yields. The yields were, however, lower compared to the reaction with acetic anhydride (85% of **4**). This methodology could be exploited in the synthesis where pivaloyl (Piv) or benzoyl (Bz) protected hydroxyl intermediate would be beneficial for subsequent reaction steps. Moreover, these protecting groups could be easily removed in situ. The reaction, though, did not proceed with *p*-toluenesulfonic anhydride (**3d**, no product observed; the expected product **48** would be probably too reactive/unstable), nor with stearic anhydride (**3f**, traces of product **50** observed in UPLC-MS, impossible to isolate due to its high lipophilicity).

Interestingly, when (*S*,*S*)-2-(6-methoxynaphthalene-2-yl)propionic anhydride (**3e**, Naproxen anhydride) [27], a chiral derivatizing reagent applicable in enantioresolution of certain pharmaceuticals, was treated with **1a** and **2a** (general procedure B), the desired product **49** was obtained in a 20% yield as a mixture of two inseparable diastereomers in 1:1 ratio. No enantioselective reaction was observed in this case.

When anhydrides were replaced with the corresponding acyl chlorides, the glycosylation did not proceed well. The reaction of **1a** and **2a** with acetyl chloride (**3g**) or benzoyl chloride (**3i**) did not afford any isolable product (only traces of **4** and **47** were detected by UPLC-MS), while analogous procedure with anhydrides afforded target compounds **4** and **47** in 85% and 39% yields (Scheme 5). On the other hand, when decanoyl chloride (**3h**) was employed, the reaction afforded the expected acylated product **51** in a 35% yield. In certain cases it may be desirable to prepare lipophilic compounds (e.g., in order to be cell penetrable) and also their isolation can be conveniently done with simple extraction into an organic solvent.

**Scheme 6:**
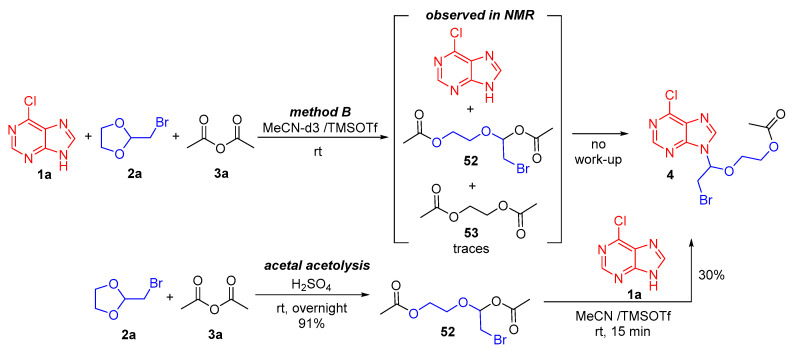
Study of the reaction course and synthesis of intermediate **52**.

In order to reveal the plausible reaction mechanism, the reaction (general procedure B with **1a**, **2a**, and **3a**) was run in deuterated MeCN directly in the NMR tube. Intermediate **52** (Scheme 6) was observed immediately after the reaction set up, together with a small amount of ethylene glycol diacetate (**53**) formed as a by-product (its structure was compared with commercially available sample). In order to confirm the structure of compound **52**, it was prepared independently in a 91% yield by treatment of 2-bromomethyl-1,3-dioxolane (**2a**) and acetic anhydride (**3a**) in the presence of sulfuric acid (a modified procedure of Rosowsky et al. [28] for the preparation of 2-acetoxyethyl acetoxymethyl ether). Compound **52** was fully characterized and its NMR spectra were identical with those of the intermediate observed during the NMR experiment. Thus, the first reaction step of the synthesis of acyclic nucleosides is acetolysis of the corresponding acetal, in this case of compound **2a**. We also performed reaction of compound **52** with 6-chloropurine (**1a**) and TMSOTf (1.5 equivalent) in MeCN (analogy to general procedure B) and product **4** (Scheme 6) was isolated in a 30% yield. Thus, the multi-component one-pot approach affords much better yields of the target compound **4** (85% versus 30%) and eliminates the reaction step needed for preparation of the alkylating agent (e.g., compound **52**).

## 3. Materials and Methods 

General Methods. Unless otherwise stated, solvents were evaporated at 40 °C/2 kPa and prepared compounds were dried at 30 °C at 2 kPa. Reaction flasks were heated in aluminum heating blocks. Tetrahydrofuran, dioxane, and acetonitrile were dried by activated neutral alumina (Drysphere). Dimethylformamide was dried by activated molecular sieves (3 Å). Other dry solvents were purchased from commercial suppliers. Analytical TLC was performed on silica gel pre-coated aluminum plates with the fluorescent indicator Merck 60 F254 (Sigma-Aldrich, Prague, Czech Republic). Flash column chromatography was carried out by Teledyne ISCO CombiFlash Rf200 with a dual absorbance detector (Teledyne ISCO, Lincoln, NE, USA). HRMS spectra (ESI^+^ or EI^+^) were recorded on LTQ Orbitrap XL spectrometer (Thermo Fisher Scientific, Waltham, MA, USA) with the ESI or EI ionization method. NMR spectra were recorded on Bruker (Rheinstetten, Germany) Avance III (400 MHz or 500 MHz) spectrometers and referenced to the residual solvent signal or a specified additive (see Supplementary Materials). The structure determination for all compounds and the assignment of NMR signals was done with the help of a combination of 1D proton and carbon experiments with 2D H,H-COSY, H,C-HSQC, and H,C-HMBC experiments. Standard experiments from the Bruker pulse-sequence library were used. Purity of compounds was measured on Waters UPLC-MS system (Santa Clara, CA, USA) that consisted of Waters UPLC H-Class Core System (column Waters ACQUITY UPLC BEH C18 1.7 mm, 2.1 × 100 mm), Waters ACQUITY UPLC PDA detector and Mass spectrometer Waters SQD2. The universal LC method was used (eluent H_2_O/MeCN, gradient 0–100%, run length 7 min) and MS method (ESI^+^ and/or ESI^−^, cone voltage = 30 V, mass detector range 100–1000 Da). Purity of the final compounds was >95%, unless otherwise stated.

Preparation of starting material. Starting compounds and reagents were purchased from commercial suppliers (Sigma-Aldrich, Prague, Czech Republic; Fluorochem, Hadfield, UK; Fisher Scientific, Pittsburgh, PA, USA; Carbosynth, Compton, UK; TCI Europe, Zwijndrecht, Belgium) and used without further purification. In several cases, we had to prepare the starting material that was not commercially available. Compounds **2d** [29], **2i** [30], and **2m** [31] were prepared according to the published procedures.

*2-(1,2-Dihydroxyethyl)-1,3-dioxolane-1,3-diacetate (***2b***).* First, 2-vinyl-1,3-dioxolane was prepared using modified procedure reported by Coates et al. [32]: a mixture of acrolein (126 g, 147 mL, 2.2 mol), ethane-1,2-diol (126 g, 113 mL, 2.0 mol) and pyridinium *p*-toluenesulfonate (1.51 g, 6.0 mmol) in pentane (300 mL) and diethyl ether (100 mL) was refluxed (45 °C) for 1 week using azeotropic apparatus. Acrolein and solvents were distilled off in vacuo. Distillation of the crude reaction mixture (120 °C, ambient pressure) afforded 2-vinyl-1,3-dioxolane (55.5 g, 24%) as bright colorless liquid. ^1^H- and ^13^C-NMR spectra were in accordance with the literature [32]. Secondly, 1-(1,3-dioxolan-2-yl)ethane-1,2-diol was prepared: 4% aq. solution of OsO_4_ (5 mL) was added to a stirred solution of 2-vinyl-1,3-dioxolane (20 g, 0.20 mol) in a mixture of acetone/water (5:1, 100 mL) and the resulting mixture was stirred at rt for 10 min. *N*-Methylmorpholine *N*-oxide (25.8 g, 0.22 mol) was added and the mixture was stirred at rt overnight. A 50% aq. solution of sodium thiosulfate (5 mL) was added and the mixture stirred for additional 30 min. Solvents were removed in vacuo and resulting slurry was dissolved in a mixture of chloroform/methanol (5:1, 150 mL) and filtered through a 5 cm silica gel column. Solvents were removed in vacuo to give 1-(1,3-dioxolan-2-yl)ethane-1,2-diol (21.7 g, 81%) as yellow viscous oil. ^1^H-NMR (400 MHz, CDCl_3_): δ = 4.89–4.83 (m, 1H, O-CH-O), 4.06–3.82 (m, 4H, O-CH_2_-CH_2_-O), 3.76–3.63 (m, 3H, H-1, H-2 and H-3), 3.29 (bs, 2H, OH). ^13^C-NMR (100 MHz, CDCl_3_): δ = 103.7 (C-1), 72.1 (C-2), 65.4 (O-CH_2_-CH_2_-O), 65.2 (O-CH_2_-CH_2_-O), 62.8 (C-3). HRMS (EI^+^) *m*/*z*: [M + H]^+^ calcd for C_5_H_11_O_4_, 135.0657; found, 135.0654. Finally, acetic anhydride (10 mL) was added to a solution of 1-(1,3-dioxolan-2-yl)ethane-1,2-diol (5.0 g, 37.3 mmol) in dry pyridine (50 mL) and the resulting mixture was stirred at room temperature overnight. Volatiles were removed in vacuo and the residue was suspended in water (50 mL). The suspension was extracted chloroform (3 × 50 mL). The combined organic fractions were washed with brine (50 mL), dried over MgSO_4_, and evaporated to give **2b** (4.32 g, 53%) as yellowish viscous oil. ^1^H-NMR (400 MHz, CDCl_3_): δ = 5.03 (ddd, *J*(2,3b) = 6.8 Hz, *J*(2,1) = 4.5 Hz, *J*(2,3a) = 3.1 Hz, 1H, H-2), 4.97 (d, *J*(1,2) = 4.5 Hz, 1H, H-1), 4.31 (dd, *J*(gem) = 12.1 Hz, *J*(3a,2) = 3.1 Hz, 1H, H-3a), 4.09 (dd, *J*(gem) = 12.1 Hz, *J*(3b,2) = 6.8 Hz, 1H, H-3b), 3.91 (m, 2H, O-CH_2_-CH_2_-O), 3.83 (m, 2H, O-CH_2_-CH_2_-O), 2.03 (s, 3H, 2-O-COCH_3_), 1.79 (s, 3H, 3-O-COCH_3_). ^13^C-NMR (100 MHz, CDCl_3_): δ = 170.6 (3-O-CO), 170.0 (2-O-CO), 101.3 (C1), 71.1 (C2), 65.5 (O-CH_2_-CH_2_-O), 65.1 (O-CH_2_-CH_2_-O), 62.0 (C-3), 20.8 (2-O-COCH_3_), 20.7 (3-O-COCH_3_). HRMS (ESI^+^) *m*/*z*: [M + Na]^+^ calcd for C_9_H_14_O_6_Na, 241.0688; found, 241.0683.

*1,3-Dioxan-5-yl 4-methylbenzenesulfonate (***2i***)* [30]. *p*-Toluenesulfonyl chloride (38.1 g, 0.20 mol) was added to a solution of glycerol formal (20.8 g, 0.20 mol, a mixture of 67% of 1,3-dioxan-5-ol and 33% of (1,3-dioxolan-4-yl)methanol) in dry pyridine (200 mL) cooled to 0 °C. The mixture was stirred at 0 °C for 30 min and then at room temperature for 24 h. Aq. 6N HCl (0.5 L) was added and the mixture was extracted EtOAc (4 × 200 mL). Combined organic layers were washed with 1N HCl (2 × 200 mL), brine (200 mL), dried over MgSO_4_, and evaporated. The crude product was dissolved in diethyl ether (500 mL) from which pure product crystallized to give **2i** (17.0 g, 33%) as white crystals. Analytical data were in accordance with the literature [30]. The evaporated filtrate afforded (1,3-dioxolan-4-yl)methyl 4-methylbenzenesulfonate (6.3 g, 12%) as a whitish solid, which was used further in the synthesis of compound **2n**.

*Benzyl (3aR, 6aS)-tetrahydro-5H-[1,3]dioxolo[4,5-c]pyrrole-5-carboxylate (***2k***).* First, a treatment of benzyl 3-pyrroline-1-carboxylate (1.5 g, 7.38 mmol), *N*-methylmorpholine *N*-oxide (1.3 g, 11.0 mmol) and 4% aq. OsO_4_ solution (0.5 mL) in a THF/water mixture (5:1, 30 mL) afforded benzyl (*3S*,*4R*)-3,4-dihydroxypyrrolidine-1-carboxylate (1.5 g, 86%) as light brown viscous oil, which was used directly in the next step. Benzyl (*3S*,*4R*)-3,4-dihydroxypyrrolidine-1-carboxylate (1.27 g, 5.35 mmol) and dimethoxymethane (0.26 mL, 5.35 mmol) were dissolved in dry DCM (30 mL). BF_3_·Et_2_O (0.68 mL, 5.35 mmol) was added in one portion and the reaction mixture was stirred at room temperature for 15 min. Another portion of dimethoxymethane (0.26 mL, 5.35 mmol) was added and the mixture was stirred at rt for additional 15 min. Water (20 mL) was added, the mixture was extracted with DCM (3 × 20 mL) and combined organic layers washed with brine, dried over MgSO_4_ and evaporated to give the crude product. Column chromatography on silica gel (3% methanol in chloroform) gave **2k** (750 mg, 56%) as light brown viscous oil. ^1^H-NMR (400 MHz, CDCl_3_): δ = 7.35 (m, 5H, H-*o*-Bn, H-*m*-Bn, H-*p*-Bn ), 5.13 (s, 2H, Bn-CH_2_), 5.08 (s, 1H, O-CH_2_a-O), 4.89 (s, 1H, O-CH_2_b-O), 4.67 (dt, *J*(*vic*) = 3.7 Hz, *J*(2-3b) = 1.0 Hz, 2H, CH-O), 3.82 (bs, 2H, N-CH_2_a), 3.44 (m, 2H, N-CH_2_b). ^13^C-NMR (100 MHz, CDCl_3_): δ = 154.1 (C=O), 136.7 (C-*i*-Bn), 128.6 (C-*o*-Bn), 128.2 (C-*p*-Bn), 128.1 (C-*m*-Bn), 96.0 (O-CH_2_-O), 79.5 (CH-O ), 78.6 (CH-O ), 67.2 (1′-CH_2_), 51.3 (N-CH_2_). HRMS (ESI^+^) *m*/*z*: [M]^+^ calcd for C_13_H_15_NO_4_, 249.1001; found, 249.1003.

*4-(Azidomethyl)-1,3-dioxolane (***2n***).* A mixture of (1,3-dioxolan-4-yl)methyl 4-methylbenzenesulfonate (5.0 g, 19.4 mmol) and sodium azide (2 g, 30.8 mmol) in dry DMSO (30 mL) was stirred at 90 °C for 6 h. The mixture was then poured into water (50 mL) and extracted with diethyl ether (3 × 50 mL). Combined organic layers were washed with brine (50 mL), dried over MgSO_4_, and evaporated. The crude mixture was purified by flash chromatography (silica gel, cHex to 30% EtOAc linear gradient) to obtain **2n** (1.25 g, 50%) as colorless liquid. ^1^H-NMR (400 MHz, CDCl_3_): δ = 5.05 (s, 1H, O-CH_2_a-O), 4.86 (s, 1H, O-CH_2_b-O), 4.20 (ddt, *J*(CH,CH_2_a) = 6.8 Hz, *J*(CH,CH_2_b) = 5.6 Hz, *J*(CH,CH_2_N_3_) = 4.5 Hz, 1H, CH), 3.94 (dd, *J*(*gem*) = 8.5 Hz, *J*(CH,CH_2_a) = 6.8 Hz, 1H, O-CH_2_a), 3.70 (dd, *J*(*gem*) = 8.5 Hz, *J*(CH,CH_2_b) = 5.6 Hz, 1H, O-CH_2_a), 3.44–3.25 (m, 2H, CH_2_-N_3_). ^13^C-NMR (100 MHz, CDCl_3_): δ = 95.5 (O-CH_2_-O), 74.3 (CH), 67.3 (O-CH_2_), 52.5 (CH_2_-N_3_). HRMS (EI^+^) *m*/*z*: no peak observed.

*2,2-Diethoxyethan-1-ol (***2q***).* 2-Hydroxyacetaldehyde (50 g, 0.28 mol) was added in one portion to 1M solution of NaOH in ethanol (285 mL) and the mixture was stirred at room temperature for 15 min. Then volatiles were removed in vacuo and the residue dissolved in water (50 mL). The solution was extracted with chloroform (3 × 150 mL) and combined organic layers were washed with brine (150 mL), dried over MgSO_4_, and chloroform was carefully evaporated in vacuo. The residue was distillated (30 °C, 5.85 torr) to give **2q** (34.6 g, 91%) as colorless liquid. ^1^H-NMR (400 MHz, DMSO-*d*_6_): δ = 4.63 (t, *J*(CH_2_-OH) = 6.1 Hz, 1H, OH), 4.40 (t, *J*(CH-CH_2_) = 5.4 Hz, 1H, CH), 3.41–3.64 (m, 4H, CH_2_-CH_3_), 3.36–3.29 (m, 2H, CH_2_-OH), 1.11 (t, *J*(CH_2_-CH_3_) = 7.0 Hz,). ^13^C-NMR (100 MHz, DMSO-*d*_6_): δ = 102.5 (CH), 62.0 (CH_2_-OH), 61.4 (CH_2_-CH_3_), 15.3 (CH_3_). HRMS (EI^+^) *m*/*z*: [M + Na]^+^ calcd for C_6_H_14_O_3_Na, 157.0841; found, 157.0839.

*2,2-Diethoxyethyl acetate (***2t***).* A mixture of bromoacetaldehyde diethyl acetal (53 g, 0.27 mol), potassium acetate (26 g, 0.27 mol), and tetrabutylammonium bromide (43 g, 0.13 mol) in dry acetonitrile (400 mL) was heated at 130 °C overnight. The mixture was cooled to room temperature, water (100 mL) was added and the mixture was extracted diethyl ether (3 × 30 mL). Combined organic layers were washed with brine, dried over MgSO_4_ and evaporated. The crude product vas distilled (28 °C, 7.10-2 mbar) to give **2t** (38 g, 80%) as colorless liquid. ^1^H-NMR (400 MHz, CDCl_3_): δ = 6.53 (t, *J* (CH_2_,CH) = 5.3 Hz, 1H, H-2), 5.94 (d, *J* (CH_2_,CH) = 5.3 Hz, 2H, H-1), 5.57 (m, 2H, O-CH_2_-CH_3_), 5.43 (m, 2H, O-CH_2_-CH_3_), 3.94 (s, 3H, OAc-CH_3_), 3.08 (t, *J*(CH_3_,CH_2_) = 7.1 Hz, 6H, CH_3_). ^13^C-NMR (100 MHz, CDCl_3_): δ = (101 MHz, CDCl_3_) δ 170.6 (C=O), 99.5 (C-2), 63.9 (C-1), 62.3 (O-CH_2_-CH_3_), 20.8 (OAcCH_3_), 15.2 (O-CH_2_-CH_3_). HRMS (EI^+^) *m*/*z*: [M + Na]^+^ calcd for C_8_H_16_O_4_Na, 199.0946; found, 199.0939.

*Tetrahydrofuro[3,4-d][1,3]dioxole (***2v***)* [33]. First, a treatment of 2,5-dihydrofuran (2.0 g, 28.5 mmol), *N*-methylmorpholine *N*-oxide (5.0 g, 42.8 mmol), and 4% aq. OsO_4_ solution (0.7 mL) in an acetone/water mixture (5:1, 30 mL) afforded (*3R*,*4S*)-tetrahydrofuran-3,4-diol (2.5 g, 83%) as viscous colorless oil. (*3R*,*4S*)-tetrahydrofuran-3,4-diol (2.1 g, 20 mmol) and dimethoxymethane (2.1 g, 2.3 mL, 26.2 mmol) were dissolved in dry DCM (20 mL). BF_3_·Et_2_O (2.9 g, 2.6 mL, 20.0 mmol) was added in one portion to the solution and the mixture was stirred at room temperature for 2 h. Water (50 mL) was added and the mixture was extracted with DCM (3 × 50 mL). Combined organic layers were washed with brine, dried over MgSO_4_, and carefully evaporated to give the crude product, which was redistilled to afford **2v** (2.0 g, 85%) as light brown liquid. Product was stored under molecular sieves in order to remove any traces of water. ^1^H-NMR (400 MHz, CDCl_3_): δ = 5.05 (s, 1H, O-CHa-O), 4.89 (s, 1H, O-CHb-O), 4.67 (m, 2H, CH), 4.08 (m, 2H, CH_2_a), 3.47 (m, 2H, CH_2_b). ^13^C-NMR (100 MHz, CDCl_3_): δ = 96.5 (O-CH-O), 80.3 (CH), 73.8 (CH_2_). HRMS (EI^+^) *m*/*z*: [M]^+^ calcd for C_5_H_8_O_3_, 116.0473; found, 116.0475.

*(S,S)-2-(6-Methoxynaphthalene-2-yl)propionic anhydride (***3e***)* [34]. *N*,*N*′-Dicyclohexylcarbodiimide (1.1 g, 5.33 mmol) was added to a solution of (*S*)-2-(6-methoxy-2-naphthyl)propionic acid (2.91 g, 2.6 mL, 20 mmol) and DMAP (80 mg, 0.71 mmol) in dry DCM (100 mL). The mixture was stirred at room temperature for 2 h. Resulting slurry was then filtered and the filtrate evaporated to give 3e (1.0 g, 52%) as white amorphous solid. ^1^H-NMR (500 MHz, CDCl_3_): *δ* = 7.50 (d, *J*(4,3) = 8.5 Hz, 2H, H-4), 7.49 (d, *J*(8,7) = 8.9 Hz, 2H, H-8), 7.40 (d, *J*(1,3) = 2.0 Hz, 2H, H-1), 7.13 (dd, *J*(3,4) = 8.5 Hz, *J*(3,1) = 2.0 Hz, 2H, H-3), 7.10 (dd, *J*(7,8) = 8.9 Hz, *J*(7,5) = 2.6 Hz, 2H, H-7), 7.03 (d, *J*(5,7) = 2.6 Hz, 2H, H-5), 3.93 (s, 6H, CH_3_O), 3.80 (q, *J*(CH,CH_3_) = 7.1 Hz, 2H, CH_3_-CH), 1.49 (d, *J*(CH_3_,CH) = 7.1 Hz, 6H, CH_3_). ^13^C-NMR (125 MHz, CDCl_3_) *δ* = 170.0 (COO), 157.7 (C-6), 133.7 (C-4a), 133.6 (C-2), 129.2 (C-8), 128.8 (C-8a), 127.3 (C-4), 126.3 (C1), 125.8 (C3), 119.0 (C-7), 105.5 (C-5), 55.3 (CH_3_O), 46.3 (CH_3_-CH), 17.8 (CH_3_-CH). HRMS (ESI^+^) *m*/*z*: [M + Na]^+^ calcd for C_28_H_26_O_5_Na, 465.1672; found, 465.1668.

Preparation of acyclic nucleoside analogues. General procedure A. The 6-Chloropurine (**1a**, 310 mg, 2.0 mmol), 2-bromomethyl-1,3-dioxolane (**2a**, 334 mg, 0.21 mL, 2.0 mmol) and acetic anhydride (**3a**, 204 mg, 0.2 mL, 2.0 mmol) were dissolved in dry acetonitrile (10 mL, or other solvent). Corresponding Lewis acid (2.0 mmol), e.g., trimethylsilyl trifluoromethanesulfonate (TMSOTf, 445 mg, 0.36 mL, 2.0 mmol), was added in one portion (either at room temperature or at 70 °C) and the reaction mixture was stirred for 15 min at the same temperature. The mixture was then poured into water (50 mL) and stirred for additional 2 min at room temperature. The slurry was washed with chloroform (3 × 50 mL) and combined organic layers were washed with brine, dried over MgSO_4_ and evaporated. The crude product was purified with flash chromatography on silica gel using gradient CHCl_3_ to 10% MeOH to obtain the pure product.

General synthetic procedure B. The 6-Chloropurine (**1a**, 310 mg, 2.0 mmol), 2-bromomethyl-1,3-dioxolane (**2a**, 334 mg, 0.21 mL, 2.0 mmol) and corresponding acid anhydride or acyl chloride, e.g., acetic anhydride (**3a**, 204 mg, 0.2 mL, 2.0 mmol), were dissolved in dry acetonitrile (10 mL). Corresponding Lewis acid (3.0 mmol), e.g., TMSOTf (667 mg, 0.54 mL, 3.0 mmol), was added in one portion at room temperature and the reaction mixture was stirred for 15 min at room temperature. Reaction work up as in General procedure A.

*2-(2-Bromo-1-(6-chloro-9H-purin-7-yl)ethoxy)ethyl acetate (***4***) and 2-(2-bromo-1-(6-chloro-7H-purin-7-yl)ethoxy)ethyl acetate (***5***).***Method A:** treatment of 6-chloropurine (**1a**, 310 mg, 2.0 mmol) and 2-bromomethyl-1,3-dioxolane (**2a**, 334 mg, 0.21 mL, 2.0 mmol) in MeCN by synthetic procedure A afforded **4** (508 mg, 83%) as yellowish viscous oil. **Method B:** treatment of 6-chloropurine (**1a**, 310 mg, 2.0 mmol) and 2-bromomethyl-1,3-dioxolane (**2a**, 334 mg, 0.21 mL, 2.0 mmol) by synthetic procedure B afforded **4** (520 mg, 85%) as yellowish viscous oil. **Method C:** treatment of 6-chloropurine (**1a**, 310 mg, 2.0 mmol) and 2-bromomethyl-1,3-dioxolane (**2a**, 334 mg, 0.21 mL, 2.0 mmol) in nitromethane by synthetic procedure A afforded **4** (380 mg, 62%) and **5** (31 mg, 5%) as yellowish viscous oils. Compound **4**: ^1^H-NMR (500 MHz, DMSO-d_6_): δ = 8.95 (s, 1H, H-8), 8.85 (s, 1H, H-2), 6.14 (dd, J(1′,2′b) = 7.1 Hz, J(1′,2′a) = 5.8 Hz, 1H, H-1′), 4.23 (dd, J(gem) = 10.8 Hz, J(2′b,1′) = 7.1 Hz, 1H, H-2′b), 4.15 (dd, J(gem) = 10.8 Hz, J(2′a,1′) = 5.8 Hz, 1H, H-2′a), 4.09 (ddd, J(gem) = 12.4 Hz, J(CH_2_b,CH_2_a) = 6.6 Hz, J(CH_2_b,CH_2_b) = 2.8 Hz, 1H, COO-CH_2_b-CH_2_-O), 4.01 (ddd, J(gem) = 12.4 Hz, J(CH_2_a,CH_2_b) = 6.0 Hz, J(CH_2_a,CH_2_a) = 2.8 Hz, 1H, COO-CH_2_a-CH_2_-O), 3.86 (ddd, J(gem) = 11.7 Hz, J(CH_2_b,CH_2_a) = 6.0 Hz, J(CH_2_b,CH_2_b) = 2.8 Hz, 1H, COO-CH_2_-CH_2_b-O), 3.62 (ddd, J(gem) = 11.7 Hz, J(CH_2_a,CH_2_b) = 6.7 Hz, J(CH_2_a,CH_2_a) = 2.8 Hz, 1H, COO-CH_2_-CH_2_a-O), 1.88 (s, 3H, CH_3_COO); ^13^C-NMR (125 MHz, DMSO-d_6_): δ = 170.3 (COO), 152.3 (C-2), 152.1 (C-4), 149.6 (C-6), 145.8 (C-8), 131.3 (C-5), 84.2 (C-1′), 68.8 (COO-CH_2_-CH_2_-O), 62.8 (COO-CH_2_-CH_2_-O), 31.8 (C-2′), 20.7 (CH_3_-COO); HRMS (ESI^+^) m/z: [M + Na]^+^ calcd. for C_11_H_12_O_3_N_4_BrClNa, 384.9674; found, 384.9675. Compound **5**: ^1^H NMR (400 MHz, DMSO-d6): δ = 9.07 (s, 1H, H-8), 8.88 (s, 1H, H-2), 6.41 (t, J(1′-2′) = 6.0 Hz, 1H, H-1′), 4.11 (m, 4H, H-2′, CH_2_-OAc), 3.93 (ddd, J(gem) = 11.7 Hz, J(CH2-CH2) = 5.7 Hz, J(CH2-CH2) = 2.9 Hz, 1H, 1’-O-CH_2_b), 3.75 (ddd, J(gem) = 11.6 Hz, J(CH2-CH2) = 6.5 Hz, J(CH2-CH2) = 2.9 Hz, 1H, 1′-O-CH_2_a), 1.91 (s, 3H, CH_3_); ^13^C-NMR (101 MHz, DMSO-d6) δ = 170.1 (COO), 161.8 (C-4), 151.9 (C-2), 148.8 (C-8), 142.2 (C-6), 122.0 (C-5), 85.7 (C-1′), 67.6 (1′-O-CH_2_), 62.6 (CH_2_-OAc), 33.4 (C-2′), 20.5 (CH_3_); HRMS (ESI^+^): m/z: [M + Na]^+^ calcd. for C_11_H_12_O_3_N_4_BrClNa, 384.9674, found, 384.9679.

*3-(2-Acetoxyethoxy)-3-(6-chloro-9H-purin-9-yl)propane-1,2-diyl diacetate (***6***).* Treatment of 6-chloropurine (**1a**, 310 mg, 2.0 mmol) and 2-(1,2-dihydroxyethyl)-1,3-dioxolane-1,3-diacetate (**2b**, 439 mg, 2.0 mmol) by synthetic procedure B afforded **6** (413 mg, 72%) as a white amorphous solid. ^1^H-NMR (500 MHz, DMSO-*d*_6_): *δ* = 8.83–8.89 (m, 2H, H-2 and H-8), 6.19–6.10 (m, 1H, H-1′), 5.75–5.65 (m, 1H, H-2′), 4.41–4.25 (m, 2H, H-3′), 3.99–4.14 (m, 2H, 1′-O-CH_2_-CH_2_), 3.60–3.69 and 3.76–3.83 (m, 2H, 1′-O-CH_2_), 2.05, 2.00, 1.94, 1.93, 1.90 and 1.76 (6×s, 9H, CH_3_). ^13^C-NMR (125 MHz, DMSO-*d*_6_): *δ* = 169.1–170.3 (m, COO), 152.3 and 151.8 (C-4), 152.24 and 152.21 (C-2), 149.5 (C-6), 146.3 and 145.7 (C-8), 131.4 and 131.2 (C-5), 83.5 and 82.9 (C-1′), 70.4 and 70.3 (C-2′), 68.0 and 67.7 (1′-O-CH_2_), 62.8 and 62.5 (1′-O-CH_2_-CH_2_), 61.9 and 61.4 (C-3′), 20.3–20.6 (m, CH_3_). HRMS (ESI^+^) *m*/*z*: [M + Na]^+^ calcd. for C_16_H_19_O_7_N_4_ClNa, 437.0835; found, 437.0835.

*3-((6-Chloro-9H-purin-9-yl)methoxy)propyl acetate (***7***).* Treatment of 6-chloropurine (**1a**, 310 mg, 2.0 mmol) and 1,3-dioxane (**2c**, 283 mg, 0.28 mL, 2.0 mmol) by synthetic procedure B afforded **7** (720 mg, 78%) as a white amorphous solid. ^1^H-NMR (500 MHz, DMSO-*d*_6_): *δ* = 8.85 (s, 1H, H-8), 8.82 (s, 1H, H-2), 5.69 (s, 2H, H-1′), 3.92 (t, *J*(4′,3′) = 6.4 Hz, 2H, H-4′), 3.57 (t, *J*(2′,3′) = 6.2 Hz, 2H, H-2′), 1.88 (s, 3H, CH_3_), 1.75 (p, *J*(3′,2′) = 6.4 Hz, *J*(3′,4′) = 6.4 Hz, 2H, H-3′). ^13^C-NMR (125 MHz, DMSO-*d*_6_): *δ* = 170.4 (COO), 152.3 (C-4), 152.2 (C-2), 149.4 (C-6), 147.9 (C-8), 131.0 (C-5), 73.1 (C-1′), 65.7 (C-2′), 60.8 (C-4′), 28.2 (C-3′), 20.7 (CH_3_). HRMS (ESI^+^) *m*/*z*: [M + H]^+^ calcd for C_11_H_14_O_3_N_4_Cl, 285.0749; found, 285.0748.

*S-(2-((6-Chloro-9H-purin-9-yl)methoxy)ethyl) ethanethioate (***8***) and S-(2-((6-chloro-7H-purin-7-yl)methoxy)ethyl) ethanethioate (***9***).* Treatment of 6-chloropurine (**1a**, 310 mg, 2.0 mmol) and 1,3-oxathiolane (**2d**, 180 mg, 2.0 mmol) by synthetic procedure B afforded **8** (86 mg, 15%) and **9** (57 mg, 10%) as brownish oils that solidified upon standing. Compound **8**: ^1^H-NMR (500 MHz, DMSO-d_6_): δ = 8.84 (s, 1H, H-8), 8.83 (s, 1H, H-2), 5.70 (s, 2H, N-CH_2_-O), 3.65 (t, J(CH_2_,CH_2_) = 6.2 Hz, 2H, CH_2_-CH_2_-S), 2.97 (t, J(CH_2_,CH_2_) = 6.2 Hz, 2H, CH_2_-S), 2.25 (s, 3H, CH_3_); ^13^C-NMR (125 MHz, DMSO-d_6_) δ = 195.0 (C=O), 152.3 (C-4), 152.3 (C-2), 149.5 (C-6), 147.9 (C-8), 131.1 (C-5), 72.9 (N-CH_2_-O), 67.9 (CH_2_-CH_2_-S), 30.6 (CH_3_), 28.3 (CH_2_-S); HRMS (EI^+^) m/z: [M]^+^ calcd. for C_10_H_11_ClN_4_O_2_S, 286.0291; found, 286.0287. Compound **9**: ^1^H-NMR (500 MHz, DMSO-d_6_): δ = 8.98 (s, 1H, H-8), 8.86 (s, 1H, H-2), 5.82 (s, 2H, N-CH_2_-O), 3.59 (t, J(CH_2_,CH_2_) = 6.2 Hz, 2H, CH_2_-CH_2_-S), 2.97 (t, J(CH_2_,CH_2_) = 6.2 Hz, 2H, CH_2_-S), 2.21 (s, 3H, CH_3_); ^13^C-NMR (125 MHz, DMSO-d_6_): δ = 195.0 (C=O), 162.3 (C-4), 152.4 (C-2), 151.4 (C-8), 143.1 (C-6), 122.1 (C-5), 75.4 (N-CH_2_-O), 66.8 (CH_2_-CH_2_-S), 30.5 (CH_3_), 28.3 (CH_2_-S); HRMS (EI^+^) m/z: [M]^+^ calcd. for C_10_H_11_ClN_4_O_2_S, 286.0291; found, 286.0292.

*2-(2-Bromo-1-(6-chloro-2-fluoro-9H-purin-9-yl)ethoxy)ethyl acetate (***12***).* Treatment of 6-chloro-2-fluoropurine (**1b**, 345 mg, 2.0 mmol) and 2-bromomethyl-1,3-dioxolane (**2a**, 334 mg, 0.21 mL, 2.0 mmol) by synthetic procedure B afforded **12** (427 mg, 56%) as a yellow oil. ^1^H-NMR (500 MHz, DMSO-*d*_6_): δ = 8.94 (s, 1H, H-8), 6.06 (dd, *J*(1′,2′b) = 6.9 Hz, *J*(1′,2′a) = 5.9 Hz, 1H, H-1′), 4.14 (dd, *J*(gem) = 10.9 Hz, *J*(2′b,1′) = 6.9 Hz, 1H, H-2′b), 4.11 (ddd, *J*(gem) = 12.4 Hz, *J*(CH_2_b,CH_2_a) = 6.5 Hz, *J*(CH_2_b,CH_2_b) = 2.9 Hz, 1H, COO-CH_2_b-CH_2_-O), 4.10 (dd, *J*(gem) = 10.9 Hz, *J*(2′a,1′) = 5.9 Hz, 1H, H-2′a), 4.03 (ddd, *J*(gem) = 12.4 Hz, *J*(CH_2_a,CH_2_b) = 6.1 Hz, *J*(CH_2_a,CH_2_a) = 2.9 Hz, 1H, COO-CH_2_a-CH_2_-O), 3.86 (ddd, *J*(gem) = 11.6 Hz, *J*(CH_2_b,CH_2_a) = 6.1 Hz, *J*(CH_2_b,CH_2_b) = 2.9 Hz, 1H, COO-CH_2_-CH_2_b-O), 3.65 (ddd, *J*(gem) = 11.6 Hz, *J*(CH_2_a,CH_2_b) = 6.5 Hz, *J*(CH_2_a,CH_2_a) = 2.9 Hz, 1H, COO-CH_2_-CH_2_a-O), 1.93 (s, 3H, CH_3_COO). ^13^C-NMR (125 MHz, DMSO-*d*_6_): δ = 170.4 (COO), 156.5 (d, *J*(2,F) = 214.6 Hz, C-2), 154.0 (d, *J*(4,F) = 17.5 Hz, C-4), 151.0 (d, *J*(6,F) = 18.2 Hz, C-6), 146.5 (d, *J*(8,F) = 2.9 Hz, C-8), 130.4 (d, *J*(5,F) = 4.9 Hz, C-5), 84.3 (C-1′), 67.8 (1′-CH_2_-CH_2_-O), 62.8 (1′-CH_2_-CH_2_-O), 31.7 (C-2′), 20.7 (CH_3_COO). HRMS (ESI^+^) *m*/*z*: [M + Na]^+^ calcd. for C_11_H_11_O_3_N_4_BrClFNa, 402.9579; found, 402.9572.

*2-(2-Bromo-1-(2,6-dichloro-9H-purin-9-yl)ethoxy)ethyl acetate (***13***).* Treatment of 2,6-dichloropurine (**1c**, 378 mg, 2.0 mmol) and 2-bromomethyl-1,3-dioxolane (**2a**, 334 mg, 0.21 mL, 2.0 mmol) by synthetic procedure B afforded **13** (421 mg, 54%) as a viscous yellow oil. ^1^H-NMR (400 MHz, DMSO-*d*_6_) δ = 8.95 (s, 1H, H-8), 6.10 (bt, *J*(CH-CH_2_) = 6.3 Hz, H-1’), 4.14 (dd, *J*(gem) = 10.9 Hz, *J*(CH_2_-CH) = 6.7 Hz, 1H, H-2’b), 4.11 (m, 1H, 1’-O-CH_2_-CH_2_b), 4.10 (dd, *J*(gem) = 10.9 Hz, *J*(CH_2_-CH) = 6.0 Hz, 1H, H-2’a), 4.04 (ddd, *J*(gem) = 12.4 Hz, *J*(CH_2_-CH_2_) = 6.1 Hz, *J*(CH_2_-CH_2_) = 2.9 Hz, 1H, 1’-O-CH_2_-CH_2_a), 3.86 (ddd, *J*(gem) = 11.7 Hz, *J*(CH_2_-CH_2_) = 6.1 Hz, *J*(CH_2_-CH_2_) = 2.9 Hz, 1H, 1’-O-CH_2_b-CH_2_), 3.66 (ddd, *J*(gem) = 11.7 Hz, *J*(CH_2_-CH_2_) = 6.5 Hz, *J*(CH_2_-CH_2_) = 2.9 Hz, 1H, 1’-O-CH_2_a-CH_2_), 1.93 (s, 3H, CH_3_OAc). ^13^C-NMR (100 MHz, DMSO-*d*_6_): δ = 170.70 (COO), 153.5 (C-4), 151.6 (C-2 or C-6), 150.2 (C-2 or C-6), 146.5 (C-8), 130.93 (C-5), 84.3 (C-1’), 67.8 (1’-O-CH_2_-CH_2_), 62.7 (1’-O-CH_2_-CH_2_), 31.8 (C-2), 20.7 (CH_3_OAc). HRMS (ESI^+^) *m*/*z*: [M + Na]^+^ calcd. for C_11_H_11_O_3_N_4_BrCl_2_Na, 418.9283; found, 418.9281.

*2-((2,6-Dichloro-9H-purin-9-yl)methoxy)ethyl acetate (***14***)* [14]. Treatment of 2,6-dichloropurine (**1c**, 378 mg, 2.0 mmol) and 1,3-dioxolane (**2g**, 148 mg, 0.14 mL, 2.0 mmol) by synthetic procedure B afforded **14** (65 mg, 11%) as a yellowish oil. Analytical data were in accordance with the literature [14]. HRMS (ESI^+^) *m*/*z*: [M + Na]^+^ calcd. for C_10_H_10_O_3_N_4_Cl_2_Na, 327.0022; found, 327.0021.

*2-((2-Amino-6-chloro-9H-purin-9-yl)methoxy)ethyl acetate (***16***)* [14]. Treatment of 2-amino-6-chloropurine (**1d**, 340 mg, 2.0 mmol) and 1,3-dioxolane (**2g**, 148 mg, 0.14 mL, 2.0 mmol) by synthetic procedure B afforded **16** (370 mg, 65%) as a viscous yellowish oil. Analytical data were in accordance with the literature [14]. HRMS (ESI^+^) *m*/*z*: [M + Na]^+^ calcd. for C_10_H_12_O_3_N_5_ClNa, 308.0521; found, 308.0523.

*3-((2-Acetamido-6-chloro-9H-purin-9-yl)methoxy)-2-(tosyloxy)propyl acetate (***17***).* Method A: treatment of 2-amino-6-chloropurine (**1d**, 340 mg, 2.0 mmol), 1,3-dioxan-5-yl 4-methylbenzenesulfonate (**2i**, 510 mg, 2.0 mmol) and acetic anhydride (**3a**, 204 mg, 0.2 mL, 2.0 mmol)by synthetic procedure B afforded **17** (502 mg, 49%) as a white amorphous solid. Method B: 2-Amino-6-chloropurine (**1d**, 340 mg, 2.0 mmol), 1,3-dioxan-5-yl 4-methylbenzenesulfonate (**2i**, 510 mg, 2.0 mmol) and acetic anhydride (**3a**, 408 mg, 0.4 mL, 4.0 mmol) were dissolved in dry acetonitrile (10 mL). TMSOTf (667 mg, 0.54 mL, 3.0 mmol), was added in one portion at room temperature and the reaction mixture was stirred for 15 min at room temperature. Reaction work up as in method A afforded **17** (605 mg, 59%) as a white amorphous solid. Method C: treatment of *N*-(6-chloro-7(9)*H*-purin-2-yl)acetamide (**1e**, 423 mg, 2.0 mmol) and 1,3-dioxan-5-yl 4-methylbenzenesulfonate (**2i**, 510 mg, 2.0 mmol) by synthetic procedure B afforded **17** (738 mg, 72%) as a white amorphous solid. ^1^H-NMR (500 MHz, DMSO-*d*_6_): *δ* = 10.88 (bs, 1H, NH), 8.57 (s, 1H, H-8), 7.69 (m, 2H, H-2″), 7.37 (m, 2H, H-3″), 5.52 (s, 2H, H-1′), 4.74 (m, 1H, H-3′), 4.02–4.10 (m, 2H, H-4′), 3.78 (dd, *J*(gem) = 11.5 Hz, *J*(2′a,3′) = 5.3 Hz, 1H, H-2′a), 3.73 (dd, *J*(gem) = 11.4 Hz, *J*(2′b,3′) = 3.8 Hz, 1H, H-2′b), 2.38 (s, 3H, 4″-CH_3_), 2.20 (s, 3H, CH_3_-CON), 1.81 (s, 3H, CH_3_-COO). ^13^C-NMR (125 MHz, DMSO-*d*_6_): *δ* = 170.0 (COO), 169.0 (CON), 153.2 (C-4), 152.6 (C-2), 149.4 (C-6), 146.5 (C-8), 145.1 (C-4″), 133.2 (C-1″), 130.1 (C-3″), 127.7 (C-2″), 127.3 (C-5), 78.4 (C-3′), 72.9 (C-1′), 67.9 (C-2′), 62.2 (C-4′), 24.8 (CH_3_-CON), 21.3 (4″-CH_3_), 20.4 (CH_3_-COO). HRMS (ESI^+^) *m*/*z*: [M + H]^+^ calcd. for C_20_H_23_O_7_N_5_ClS, 512.1001; found, 512.1002.

*Benzyl 3-((2-acetamido-6-chloro-9H-purin-9-yl)methoxy)-4-acetoxypyrrolidine-1-carboxylate (***20***).* Treatment of *N*-(6-chloro-7(9)*H*-purin-2-yl)-acetamide (**1e**, 423 mg, 2.0 mmol) and benzyl tetrahydro-5*H*-[1,3]dioxolo[4,5-c]pyrrole-5-carboxylate (**2k**, 500 mg, 2.0 mmol) by synthetic procedure B afforded **20** (640 mg, 64%) as a yellowish viscous oil. ^1^H-NMR (500 MHz, DMSO-*d*_6_): *δ* = 10.91 and 10.92 (bs, 1H, NH), 8.62 and 8.62 (s, 1H, H-8), 7.28–7.37 (m, 5H, H-2″ and H3″ and H-4″), 5.60–5.68 (m, 2H, N-CH_2_-O), 5.20–5.24 (m, 1H, H-4′), 5.00–5.07 (m, 2H, 1″-CH_3_), 4.60–4.64 (m, 1H, H-3′), 3.55–3.67 (m, 2H, H-2′a and H-5′a), 3.17–3.37 (m, 2H, H-2′b and H-5′b), 2.17 and 2.17 (s, 3H, CH_3_-CON), 1.90 and 1.91 (s, 3H, CH_3_). ^13^C-NMR (125 MHz, DMSO-*d*_6_): *δ* = 169.83 and 169.80 (CH_3_-COO), 168.90 and 168.88 (CH_3_-CON), 154.0 and 154.1 (N-COO), 153.3 and 153.3 (C-4), 152.5 (C-2 or C-6), 149.4 (C-2 or C-6), 146.6 (C-8), 137.01 and 136.98 (C-1″), 128.6 (C-3″), 128.07 and 128.05 (C-4″), 127.77 and 127.75 (C-2″), 127.4 (C-5), 75.6 and 76.1 (C-3′), 72.14 and 72.08 (N-CH_2_-O), 71.2 and 70.5 (C-4′), 66.28 and 66.26 (1″-CH_2_), 48.8 and 49.1 (C-5′), 47.9 and 48.2 (C-2′), 24.7 (CH_3_-CON), 20.70 and 20.68 (CH_3_-COO). HRMS (ESI^+^) *m*/*z*: [M + H]^+^ calcd. for C_22_H_24_O_6_N_6_Cl, 503.1440; found, 503.1444.

*2-(1-(6-Amino-4-chloro-2H-pyrazolo[3,4-d]pyrimidin-2-yl)-2-bromoethoxy)ethyl acetate (***22***).* Treatment of 4-chloro-1*H*-pyrazolo[3,4-*d*]pyrimidine-6-amine **1g** (340 mg, 2.0 mmol) and 2-bromomethyl-1,3-dioxolane (**2a**, 334 mg, 0.21 mL, 2.0 mmol) by synthetic procedure B afforded **22** (720 mg, 78%) as a white amorphous solid. ^1^H-NMR (500 MHz, DMSO-*d*_6_): δ = 8.86 (s, 1H, H-7), 8.37 (bs, 2H, NH_2_), 5.83 (t, *J*(1′,2′) = 2.9 Hz, 1H, H-1′), 4.13 (ddd, *J*(gem) = 12.3 Hz, *J*(CH_2_,CH_2_) = 6.5 Hz, *J*(CH_2_,CH_2_) = 2.9 Hz, 1H, CH_2_b-OAc), 4.03 (ddd, *J*(gem) = 12.3 Hz, *J*(CH_2_,CH_2_) = 6.2 Hz, *J*(CH_2_,CH_2_) = 3.0 Hz, 1H, CH_2_a-OAc), 3.93 (d, *J*(2′,1′) = 6.2 Hz, 2H, H-2′), 3.79 (ddd, *J*(gem) = 11.6 Hz, *J*(CH_2_,CH_2_) = 6.2 Hz, (CH_2_,CH_2_) = 2.9 Hz, 1H, 1′-O-CH_2_b), 3.55 (ddd, *J*(gem) = 11.6 Hz, *J*(CH_2_,CH_2_) = 6.5 Hz, (CH_2_,CH_2_) = 2.9 Hz, 1H, 1′-O-CH_2_a), 1.98 (s, 3H, CH_3_). ^13^C-NMR (125 MHz, DMSO-*d*_6_): δ = 170.5 (COO), 156.6 (C-2), 153.2 (C-6), 150.1 (C-4), 131.8 (C-7), 102.0 (C-5), 89.7 (C-1′), 67.4 (1′-O-CH_2_), 62.7 (CH_2_-OAc), 31.8 (C-2′), 20.9 (CH_3_). HRMS (ESI^+^) *m*/*z*: [M + H]^+^ calcd. for C_11_H_14_O_3_N_5_BrCl, 377.9963; found, 377.9965.

*2-(1-(Guanine-7-yl)-2-bromoethoxy)ethyl acetate (***23***).* Treatment of guanine (**1h**, 300 mg, 2.0 mmol) and 2-bromomethyl-1,3-dioxolane (**2a**, 334 mg, 0.21 mL, 2.0 mmol) by synthetic procedure B afforded **23** (86 mg, 15%) as a white amorphous solid. ^1^H-NMR (500 MHz, DMSO-*d*_6_): δ = 10.97 (bs,1H,1-NH), 8.24 (s, 1H, H-8), 6.26 (bs, 2H, NH_2_), 6.03 (t, *J*(1′,2′a) = *J*(1′,2′b) = 6.3 Hz, 1H, H-1′), 4.16 (dd, *J*(gem) = 10.7 Hz, *J*(2′b,1′) = 6.7 Hz, 1H, H-2′b), 4.12 (ddd, *J*(gem) = 12.3 Hz, *J*(CH_2_b,CH_2_a) = 6.8 Hz, *J*(CH_2_b,CH_2_b) = 2.9 Hz, 1H, 1′-O-CH_2_-CH_2_b-O), 4.03 (dd, *J*(gem) = 10.7 Hz, *J*(2′a,1′) = 5.9 Hz, 1H, H-2′a), 4.01 (ddd, *J*(gem) = 12.3 Hz, *J*(CH_2_a,CH_2_b) = 5.9 Hz, *J*(CH_2_a,CH_2_a) = 2.8 Hz, 1H, 1′-O-CH_2_-CH_2_a-O), 3.76 (ddd, *J*(gem) = 11.7 Hz, *J*(CH_2_b,CH_2_a) = 5.9 Hz, *J*(CH_2_b,CH_2_b) = 2.9 Hz, 1H, 1′-O-CH_2_b-CH_2_-O), 3.57 (ddd, *J*(gem) = 11.7 Hz, *J*(CH_2_a,CH_2_b) = 6.8 Hz, *J*(CH_2_a,CH_2_a) = 2.9 Hz, 1H, 1′-O-CH_2_a-CH_2_-O-), 1.95 (s, 3H, CH_3_CO). ^13^C-NMR (125 MHz, DMSO-*d*_6_): δ = 170.4 (COO), 160.7 (C-4), 154.5 (C-6), 153.2 (C-2), 142.5 (C-8), 107.7 (C-5), 85.2 (C-1′), 67.1 (1′-O-CH_2_-CH_2_-O), 62.8 (1′-O-CH_2_-CH_2_-O), 32.9 (C-2′), 20.8 (CH_3_COO). HRMS (ESI^+^) *m*/*z*: [M + Na]^+^ calcd for C_11_H_14_O_4_N_5_BrNa, 382.0121; found, 382.0118.

*2-(1-(Adenin-9-yl)-2-bromoethoxy)ethyl acetate (***24***).* Treatment of adenine (**1i**, 270 mg, 2.0 mmol) and 2-bromomethyl-1,3-dioxolane (**2a**, 334 mg, 0.21 mL, 2.0 mmol) by synthetic procedure B and modified work up (the crude reaction mixture was adsorbed on silica gel, followed by flash chromatography using gradient CHCl_3_ to 10% MeOH) afforded **24** (378 mg, 55%) as a yellow viscous oil. ^1^H-NMR (400 MHz, DMSO-*d*_6_) δ = 8.37 (s, 1H, H-8), 8.17 (s, 1H, H-2), 7.36 (bs, 2H, NH_2_), 5.94 (dd, *J*(CH_2_-CH_2_) = 7.3 Hz, *J*(CH_2_-CH_2_) = 5.7 Hz, 1H, H-1’), 4.22 (d, *J*(gem) = 10.7 Hz, *J*(CH_2_-CH_2_) = 7.3 Hz, 1H, H-2’b), 4.10 (ddd, *J*(gem) = 12.4 Hz, *J*(CH_2_-CH_2_) = 6.7 Hz, *J*(CH_2_-CH_2_) = 2.9 Hz, 1H, 1’-O-CH_2_-CH_2_b), 4.09 (dd, *J*(gem) = 10.6 Hz, *J*(CH_2_-CH) = 5.7 Hz, H-2’a), 4.00 (ddd, *J*(gem) = 12.3 Hz, *J*(CH_2_-CH_2_) = 6.0 Hz, *J*(CH_2_-CH_2_) = 2.9 Hz, 1H, 1’-O-CH_2_-CH_2_a), 3.79 (ddd, *J*(gem) = 11.6 Hz, *J*(CH_2_-CH_2_) = 6.0 Hz, *J*(CH_2_-CH_2_) = 2.9 Hz, 1H, 1’-O-CH_2_b-CH_2_), 3.55 (ddd, *J*(gem) = 11.6 Hz, *J*(CH_2_-CH_2_) = 6.7 Hz, *J*(CH_2_-CH_2_) = 2.9 Hz, 1H, 1’-O-CH_2_a-CH_2_), 1.91 (s, 3H, CH_3_). ^13^C-NMR (100 MHz, DMSO-*d*_6_): δ = 170.4 (COO), 156.2 (C-6), 153.0 (C-2), 149.8 (C-4), 139.2 (C-8), 118.8 (C-5), 83.2 (C-1’), 67.7 (1’-O-CH_2_-CH_2_), 62.8 (1’-O-CH_2_-CH_2_), 31.9 (C-2’), 20.7 (CH_3_-OAc). HRMS (ESI^+^) *m*/*z*: [M + H]^+^ calcd. for C_11_H_15_O_3_N_5_Br, 344.0353; found, 344.0348.

*2-((6-Benzamido-9H-purin-9-yl)methoxy)ethyl acetate (***25***).* Treatment of *N*^6^-benzoyladenine (**1j**, 480 mg, 2.0 mmol) and 1,3-dioxolane (**2g**, 148 mg, 0.14 mL, 2.0 mmol) by synthetic procedure B afforded **25** (500 mg, 71%) as a white amorphous solid. ^1^H-NMR (500 MHz, DMSO-*d*_6_): *δ* = 11.25 (bs, 1H, NH). 8.78 (s, 1H, H-2), 8.64 (s, 1H, H-8), 8.06 (m, 2H, H-2″), 7.65 (m, 1H, H-4″), 7.55 (m, 2H, H-3″), 5.72 (s, 2H, H-1′), 4.10 (m, 2H, H-3′)3.77, (m, 2H, H-2′), 1.93 (s, 3H, CH_3_). ^13^C-NMR (125 MHz, DMSO-*d*_6_) *δ* = 170.5 (COO), 165.9 (CON), 152.8 (C-4), 152.1 (C-2), 150.6 (C-6), 145.1 (C-8), 133.6 (C-1″), 132.6 (C-4″), 128.7 (C-2″ and C-3″), 125.4 (C-5), 72.6 (C-1′), 67.4 (C-2′), 63.0 (C-3′), 20.7 (CH_3_). HRMS (ESI^+^) *m*/*z*: [M + H]^+^ calcd for C_17_H_18_O_4_N_5_, 356.1353; found, 356.1354.

*2-((6-Benzamido-9H-purin-9-yl)methoxy)but-3-en-1-yl acetate (***26***).* Treatment of *N*^6^-benzoyladenine (**1j**, 480 mg, 2.0 mmol) and 4-vinyl-[1,3]dioxolane (**2m**, 200 mg, 0.2 mL, 2.0 mmol) by synthetic procedure B afforded **26** (430 mg, 56%) as a colorless viscous oil. ^1^H-NMR (500 MHz, DMSO-*d*_6_): *δ* = 11.23 (bs, 1H, NH), 8.78 (s, 1H, H-2), 8.63 (s, 1H, H-8), 8.05 (m, 2H, H-2″), 7.65 (m, 1H, H-4″), 7.55 (m, 2H, H-3″), 5.76 (d, *J*(gem) = 11.2 Hz, 1H, 1′a), 5.74 (ddd, *J*(CH,CH_2_) = 17.2 and 10.6, *J*(CH,2′) = 6.3 Hz, 1H, CH=CH_2_), 5.65 (d, *J*(gem) = 11.3 Hz, 1H, H-1′b), 5.65 (dt, *J*(CH_2_,CH) = 17.3 Hz, *J*(gem) = *J*(CH_2_,2′) = 1.5 Hz, 1H, CH=CH_2_a), 5.23 (dt, *J*(CH_2_,CH) = 10.5 Hz, *J*(gem) = *J*(CH_2_,2′) = 1.5 Hz, 1H, CH=CH_2_b), 4.36 (m, 1H, H-2′) 4.00 (dd, *J*(gem) = 11.7 Hz, *J*(3′a,2′) = 3.6 Hz, 1H, 3′a), 3.39 (dd, *J*(gem) = 11.7 Hz, *J*(3′b,2′) = 7.5 Hz, 1H, H-3′b), 1.81 (s, 3H, CH_3_). ^13^C-NMR (125 MHz, DMSO-*d*_6_): *δ* = 170.2 (COO), 165.8 (CON), 152.8 (C-4), 152.1 (C-2), 150.5 (C-6), 145.0 (C-8), 133.9 (CH=CH_2_), 133.6 (C-1″), 132.6 (C-4″), 128.7 (C-2″ and C-3″), 125.4 (C-5), 119.0 (CH=CH_2_), 77.4 (C-2′), 71.2 (C-1′), 65.2 (C-3′), 20.5 (CH_3_). HRMS (ESI^+^) *m*/*z*: [M + H]^+^ calcd for C_19_H_19_N_5_O_4_, 382.1510; found: 382.1511.

*2-(2-Bromo-1-(2,6-diamino-9H-purin-9-yl)ethoxy)ethyl acetate (***29***).* Treatment of 2,6-diaminopurine (**1k**, 300 mg, 2.0 mmol) and 2-bromomethyl-1,3-dioxolane (**2a**, 334 mg, 0.21 mL, 2.0 mmol) by synthetic procedure B and modified work up (the crude reaction mixture was adsorbed on silica gel, followed by flash chromatography using gradient CHCl_3_ to 10% MeOH) afforded **29** (431 mg, 60%) as a yellow viscous oil. ^1^H-NMR (400 MHz, DMSO-*d*_6_) δ = 7.91 (s, 1H, H-8), 6.76 (bs, 2H, 6-NH_2_), 5.88 (bs, 2H, 2-NH_2_), 5.70 (dd, *J*(CH_2_-CH_2_) = 6.9 Hz, *J*(CH_2_-CH_2_) = 6.0 Hz, 1H, H-1’), 4.14 (dd, *J*(gem) = 10.7 Hz, *J*(CH_2_-CH) = 7.0 Hz, 1H, H-2’b), 4.11 (ddd, *J*(gem) = 12.3 Hz, *J*(CH_2_-CH_2_) = 6.7 Hz, *J*(CH_2_-CH_2_) = 3.0 Hz, 1H, 1’-O-CH_2_-CH_2_b), 4.03 (dd, *J*(gem) = 10.5 Hz, *J*(CH_2_-CH) = 6.0 Hz, 1H, H-2’a), 4.02 (ddd, *J*(gem) = 12.3 Hz, *J*(CH_2_-CH_2_) = 6.0 Hz, *J*(CH_2_-CH_2_) = 3.0 Hz, 1H, 1’-O-CH_2_-CH_2_a), 3.73 (ddd, *J*(gem) = 11.5 Hz, *J*(CH_2_-CH_2_) = 6.0 Hz, *J*(CH_2_-CH_2_) = 3.0 Hz, 1H, 1’-O-CH_2_b-CH_2_), 3.53 (ddd, *J*(gem) = 11.5 Hz, *J*(CH_2_-CH_2_) = 6.7 Hz, *J*(CH_2_-CH_2_) = 3.0 Hz, 1H, 1’-O-CH_2_a-CH_2_), 1.94 (s, 3H, CH_3_-OAc). ^13^C-NMR (100 MHz, DMSO-*d*_6_): δ = 170.4 (COO), 160.6 (C-2), 156.3 (C-6), 152.1 (C-4), 135.5 (C-8), 113.2 (C-5), 82.7 (C-1’), 66.9 (1’-O-CH_2_-CH_2_), 62.8 (1’-O-CH_2_-CH_2_), 32.1 (C-2’), 20.7 (CH_3_-OAc). HRMS (ESI^+^) *m*/*z*: [M + H]^+^ calcd. for C_11_H_16_O_3_N_6_Br, 359.0462; found, 359.0457.

*3-(2-Acetoxyethoxy)-3-(2,6-diacetamido-9H-purin-9-yl)propane-1,2-diyl diacetate (***30***).* Treatment of 2,6-bisacetamidopurine (**1l**, 470 mg, 2.0 mmol) and 2-(1,2-dihydroxyethyl)-1,3-dioxolane-1,3-diacetate (**2b**, 439 mg, 2.0 mmol) by synthetic procedure B afforded **30** (690 mg, 70%) as a white amorphous solid. ^1^H-NMR (500 MHz, DMSO-*d*_6_): *δ* =10.59 and 10.56 (bs, 1H, C6-NH), 10.38 (bs, 1H, C2-NH), 8.46 and 8.44 (s, 1H, H-8),5.96 and 5.90 (d, *J*(1′,2′) = 7.6 Hz and *J*(1′,2′) = 6.3 Hz, 1H, H-1′), 5.74 and 5.66 (m, 1H, H-2′), 3.42–4.98 (m, 4H, H-3′ and 1′-O-CH_2_-CH_2_), 3.78–3.57 (m, 2H, 1′-O-CH_2_), 2.35, 2.34, 2.27 and 2.26 (s, 6H, CH_3_-CON), 2.06, 2.02, 1.94, 1.93, 1,92 and 1.80 (s, 9H, CH_3_-COO). ^13^C-NMR (125 MHz, DMSO-*d*_6_): *δ* = 169.1–170.4 (COO and CON), 153.1 (C-4), 152.7 (C-4 or C-6), 152.6 (C-4 or C-6), 152.6 (C-6), 149.92 and 149.85 (C-2), 141.5 and 141.9 (C-8), 119.4 and 119.5 (C-5), 82.0 and 82.7 (C-1′), 70.3 and 70.5 (C-2′), 67.4 and 67.7 (1′-O-CH_2_), 62.5 and 62.8 (1′-O-CH_2_-CH_2_), 61.5 and 62.0 (C-3′), 24.99, 24.96 and 24.92 (CH_3_-CON), 20.4–20.7 (m, CH_3_-COO). HRMS (ESI^+^) *m*/*z*: [M + H]^+^ calcd. for C_20_H_27_O_9_N_6_, 495.1834; found, 495.1835.

*6-Chloro-9-(2-chloro-1-ethoxyethyl)-9H-purine (***31***).* Treatment of 6-chloropurine (**1a**, 310 mg, 2.0 mmol) and chloroacetaldehyde diethyl acetal (**2o**, 300 mg, 0.3mL, 2.0 mmol) by synthetic procedure B afforded **31** (428 mg, 82%) as a brownish viscous oil. ^1^H-NMR (500 MHz, DMSO-*d*_6_): *δ* = 8.75 (s, 1H, H-2 ), 8.32 (s, 1H, H-8), 6.00 (dd, *J*(1′,2′a) = 6.3 Hz, *J*(1′,2′b) = 4.8 Hz, H-1′), 4.04 (dd, *J*(gem) = 11.9 Hz, *J*(2′a,1′) = 6.3 Hz, 1H, H-2′a), 3.96 (dd, *J*(gem) = 11.9 Hz, *J*(2′b,1′) = 4.8 Hz, 1H, H-2′b), 3.52 and 3.64 (2×m, 2H, 1′-O-CH_2_), 1.22 (t, *J*(CH_3_,CH_2_) = 7.00 Hz, 3H, CH_3_). ^13^C-NMR (125 MHz, DMSO-*d*_6_): *δ* = 152.3 (C-2), 151.7 (C-4), 151.4 (C-6), 143.0 (C-8), 131.8 (C-5), 84.2 (C-1′), 66.2 (O-CH_2_), 44.4 (C-2′), 14.6 (CH_3_). HRMS (ESI^+^) *m*/*z*: [M + H]^+^ calcd. for C_9_H_10_ON_4_Cl_2_Na, 283.0124; found, 283.0124.

*6-Chloro-9-(1-ethoxyprop-2-yn-1-yl)-2-fluoro-9H-purine (***34***).* Treatment of 6-chloro-2-fluoropurine (**1b**, 345 mg, 2.0 mmol) and 3,3-diethoxyprop-1-yne (**2r**, 256 mg, 2.0 mmol) by synthetic procedure B afforded **34** (183 mg, 36%) as a brownish viscous oil. ^1^H-NMR (500 MHz, DMSO-*d*_6_): *δ* = 8.93 (s, 1H, H-8), 6.69 (d, *J*(1′,3′) = 2.1 Hz, 1H, H-1′), 4.15 (d, *J*(3′,1′) = 2.1 Hz, 1H, H-3′), 3.52–3.65 (m, 2H, O-CH_2_), 1.09 (t, *J*(CH_3_,CH_2_) = 7.0 Hz, 3H, CH_3_). ^13^C-NMR (125 MHz, DMSO-*d*_6_): *δ* = 156.6 (d, *J*(2,F) = 214.9 Hz, C-2), 153.2 (d, *J*(4,F) = 17.7 Hz, C-4), 151.2 (d, *J*(6,F) = 18.1 Hz, C-6), 146.4 (d, *J*(8,F) = 3.2 Hz, C-8), 130.4 (d, *J*(5,F) = 4.8 Hz, C-5), 80.0 (C-3′), 76.6 (C-2′), 73.3 (C-1′), 63.9 (O-CH_3_), 14.7 (CH_3_). HRMS (ESI^+^) *m*/*z*: [M + H]^+^ calcd for C_10_H_8_ClFN_4_O, 254.0371; found, 254.0373.

*4-(Adenine-9-yl)-4-methoxybutan-2-one (***35***).* Treatment of adenine (**1i**, 270 mg, 2.0 mmol) and 4,4-dimethoxy-2-butanone (**2s**, 321 mg, 0.32 mL, 2.0 mmol) by synthetic procedure B afforded **35** (109 mg, 23%) as a pinkish amorphous solid. ^1^H-NMR (500 MHz, DMSO-*d*_6_): δ = 8.34 (s, 1H, H-8), 8.16 (s, 1H, H-2), 7.30 (bs, 2H, NH_2_), 5.92 (dd, *J*(1′,2′b) = 7.4 Hz, *J*(1′,2′a) = 5.4 Hz, 1H, H-1′), 3.59 (dd, *J*(gem) = 17.3 Hz, *J*(2b′,1′) = 7.4 Hz, 1H, H-2′b), 3.38 (dd, *J*(gem) = 17.3 Hz, *J*(2a′,1′) = 5.4 Hz, 1H, H-2′a), 3.13 (s, 3H, CH_3_O), 2.12 (s, 3H, H-4′). ^13^C-NMR (125 MHz, DMSO-*d*_6_): δ = 204.6 (C-3′), 156.3 (C-6), 152.9 (C-2), 149.8 (C-4), 139.8 (C-8), 119.0 (C-5), 81.6 (C-1′), 56.0 (CH_3_O), 47.3 (C-2′), 30.4 (C-4′). HRMS (ESI^+^) *m*/*z*: [M + H]^+^ calcd for C_10_H_14_O_2_N_5_, 236.1142; found, 236.1140.

*N,N’-(9-(1-Ethoxyprop-2-yn-1-yl)-9H-purine-2,6-diyl)diacetamide (***36***).* Treatment of 2,6-bisacetamidopurine (**1l**, 470 mg, 2.0 mmol) and 3,3-diethoxyprop-1-yne (**2r**, 256 mg, 2.0 mmol) by synthetic procedure B afforded **36** (210 mg, 31%) as a light brown viscous oil. ^1^H-NMR (500 MHz, DMSO-*d*_6_): *δ* = 10.62 (bs, 1H, NH), 10.42 (bs, 1H, NH), 8.53 (s, 1H, H-8), 6.52 (d, *J*(1′,3′) = 2.1 Hz, 1H, H-1′), 4.11 (d, *J*(3′,1′) = 2.1 Hz, 1H, H-3′), 3.52–3.64 (m, 2H, O-CH_2_), 2.34 (s, 3H, CH_3_-CON), 2.27 (s, 3H, CH_3_-CON), 1.10 (t, *J*(CH_2_,CH_3_) = 7.0 Hz, 3H, O-CH_2_-CH_3_). ^13^C-NMR (125 MHz, DMSO-*d*_6_): *δ* = 169.8 (CON), 152.8 (C-2 or C-6), 152.1 (C-4), 150.1 (C-2 or C-6), 141.3 (C-8), 119.4 (C-5), 79.5 (C-3′), 77.3 (C-2′), 72.2 (C-1′), 63.7 (O-CH_3_), 25.0 (CH_3_-CON), 24.9 (CH_3_-CON), 14.8 (O-CH_2_-CH_3_). HRMS (ESI^+^) *m*/*z*: [M + H]^+^ calcd. for C_14_H_16_O_3_N_6_Na, 339.1176; found, 339.1176.

*2-(4-Chloro-1H-pyrazolo[4,3-c]pyridin-1-yl)-2-ethoxyethyl acetate (***37***).* Treatment of 4-chloro-1*H*-pyrazolo[4,3-*c*]pyridine (**1m**, 306 mg, 2.0 mmol) and acetoxyacetaldehyde dimethyl acetate (**2t**, 355 mg, 2.0 mmol) by synthetic procedure B afforded **37** (370 mg, 68%) as a brownish viscous oil. ^1^H-NMR (500 MHz, DMSO-*d*_6_): *δ* = 8.44 (d, *J*(7,3) = 0.9 Hz, 1H, H-7), 8.24 (d, *J*(2,3) = 6.0 Hz, 1H, H-2), 7.93 (dd, *J*(3,2) = 6.0 Hz, *J*(3,7) = 1.0 Hz, 1H, H-3), 6.18 (dd, *J*(1′,2′a) = 6.3 Hz, *J*(1′,2′b) = 5.5 Hz, 1H, H-1′), 4.58 (dd, *J*(gem) = 11.4 Hz, *J*(2′a,1′) = 5.5 Hz, 1H, H-2′a), 4.49 (dd, *J*(gem) = 11.4 Hz, *J*(2′b,1′) = 6.3 Hz, 1H, H-2′b ), 3.56 (dq, *J*(gem) = 9.5 Hz, *J*(CH_2_,CH_3_) = 7.0 Hz, 1H, CH_3_,CH_2_a), 3.21 (dq, *J*(gem) = 9.5 Hz, *J*(CH_2_,CH_3_) = 7.1 Hz, 1H, CH_3_,CH_2_b), 1.89 (s, 3H, CH_3_-COO), 1.03 (t, *J*(CH_3_,CH_2_) = 7.0 Hz, 3H, CH_3_-CH_2_). ^13^C-NMR (125 MHz, DMSO-*d*_6_): *δ* = 169.9 (COO), 144.5 (C-4), 144.0 (C-2), 143.9 (C-6), 134.4 (C-7), 119.9 (C-5), 105.7 (C-3), 85.6 (C-1′), 64.3 (CH_2_-CH_3_), 63.3 (C-2′), 20.5 (CH_3_-COO), 14.8 (CH_2_-CH_3_). HRMS (ESI^+^) *m*/*z*: [M + H]^+^ calcd. for C_12_H_15_O_3_N_3_Cl, 284.0797; found, 284.0797.

*Ethyl 4-(4-chloro-1H-imidazo[4,5-c]pyridin-1-yl)-2-cyano-4-ethoxybutanoate (***38***).* Treatment of 4-chloro-1*H*-imidazo[4,5-*c*]pyridine (**1n**, 310 mg, 2.0 mmol) and ethyl 2-cyano-4,4-diethoxybutyrate (**2u**, 462 mg, 2.0 mmol) by synthetic procedure B afforded **38** (595 mg, 88%) as a yellow viscous oil. ^1^H-NMR (500 MHz, DMSO-*d*_6_): *δ* = 8.68 and 8.67 (s, 1H, H-8), 8.19–8.21 (m, 1H, H-2), 7.77 and 7.72 (d, *J*(3,2) = 5.6 Hz, 1H, H-3), 5.97–5.93 (m, 1H, H-1′), 4.37–4.46 (m, 1H, H-3′), 4.06–4.25 (m, 2H, COO-CH_2_), 3.48 (m, 1H, 1′-O-CH_2_), 3.23 (m, 1H, 1′-O-CH_2_), 2.55–2.87 (m, 2H, H-2′), 1.02–1.28 (m, 6H, CH_3_). ^13^C-NMR (125 MHz, DMSO-*d*_6_): *δ* = 166.1 (COO), 165.6 (COO), 145.4 and 145.3 (C-8), 141.6 and 141.5 (C-2), 141.5 (C-6), 139.0 and 138.8 (C-4), 137.8 and 137.7 (C-5), 116.7 and 116.6 (CN), 107.8 and 107.7 (C-3), 84.3 and 84.1 (C-1′), 64.7 and 64.4 (1′-O-CH_2_), 62.7 and 62.6 (COO-CH_2_), 34.0 and 33.7 (C-3′), 33.6 and 33.2 (C-2′), 14.6, 14.5, 14.1 and 13.9 (CH_3_). HRMS (ESI^+^) *m*/*z*: [M + H]^+^ calcd. for C_15_H_18_O_3_N_4_Cl, 337.1062; found, 337.1062.

*2-((1H-1,2,4-Triazol-1-yl)methoxy)ethyl acetate (***39***).* Treatment of 1,2,4-triazole (**1o**, 138 mg, 2.0 mmol) and 1,3-dioxolane (**2g**, 148 mg, 0.14 mL, 2.0 mmol) by synthetic procedure B afforded **39** (290 mg, 78%) as a colorless oil. ^1^H-NMR (500 MHz, DMSO-*d*_6_): *δ* = 8.72 (s, 1H, H-5), 8.06 (s, 1H, H-3), 5.57 (s, 2H, N-CH_2_-O), 4.08 (m, 2H, CH_2_-OAc), 3.69 (m, 2H, N-CH_2_-O-CH_2_), 1.98 (s, 3H, CH_3_). ^13^C-NMR (125 MHz, DMSO-*d*_6_): *δ* = 170.5 (COO), 152.2 (C-3), 145.4 (C-5), 77.4 (N-CH_2_-O), 67.1 (N-CH_2_-O-CH_2_), 62.9 (CH_2_-OAc), 20.81 (CH_3_). HRMS (ESI^+^) *m*/*z*: [M + H]^+^ calcd. for C_7_H_12_O_3_N_3_, 186.0873; found, 186.0873.

*3-((2-Acetoxyethoxy)methyl)thiazol-3-ium trifluoromethanesulfonate (***40***).* Treatment of thiazole (**1p**, 170 mg, 2.0 mmol) and 1,3-dioxolane (**2g**, 148 mg, 0.14 mL, 2.0 mmol) by synthetic procedure B and by modified work up (the crude product was purified using C18-silica gel flash chromatography in water to methanol, gradient 0–100%) afforded **40** (330 mg, 47%) as a colorless oil. ^1^H-NMR (500 MHz, D_2_O): *δ* = 10.18 (m, 1H, H-2), 8.47 (dd, *J*(4,5) = 3.8 Hz, *J*(4,2) = 1.4 Hz, 1H, H-4), 8.29 (dd, *J*(5,4) = 3.8 Hz, *J*(5,2) = 2.5 Hz, 1H, H-5), 5.95 (s, 2H, N-CH_2_-O), 4.26 (m, 2H, CH_2_-OAc), 3.94 (m, 2H, CH_2_-CH_2_-OAc), 2.08 (s, 3H, CH_3_). ^13^C-NMR (125 MHz, D_2_O): δ = 174.7 (COO), 159.1 (C-2), 136.3 (C-4), 127.2 (C-5), 84.3 (N-CH_2_-O), 69.6 (CH_2_-CH_2_-OAc), 64.1 (CH_2_-OAc), 20.9 (CH_3_). HRMS (ESI^+^) *m*/*z*: [M]^+^ calcd. for C_8_H_12_O_3_NS, 202.0532; found, 202.0533. 

*1-(((4-Acetoxytetrahydrofuran-3-yl)oxy)methyl)-4-nitro-1H-pyrazole-3-carboxylic acid (***41***).* Treatment of 4-nitro-1*H*-pyrazole-3-carboxylic acid (**1q**, 314 mg, 2.0 mmol) and 1,4-anhydro-2,3-*O*-methylenerythritol (**2v**, 232 mg, 2.0 mmol) by synthetic procedure B afforded **41** (252 mg, 40%) as a brownish oil. ^1^H-NMR (500 MHz, DMSO-*d*_6_): *δ* = 9.13 (s, 1H, H-5), 5.10 (td, *J*(4′,3′) = *J*(4′,5′a) = 5.4 Hz, *J*(4′,5′b) = 3.8 Hz, 1H, H-4′), 4.47 (q, *J*(3′,4′) = *J*(3′,2′a) = *J*(3′,2′b) = 5.6 Hz, 1H, H-3′), 3.92 (dd, *J*(gem) = 10.0 Hz, *J*(5′a,4′) = 5.5 Hz, 1H, H-5′a), 3.82 (dd, *J*(gem) = 9.3 Hz, *J*(2′a,3′) = 6.0 Hz, 1H, H-2′a), 3.64 (dd, *J*(gem) = 10.0 Hz, *J*(5′b,4′) = 3.9 Hz, 1H, H-5′b), 3.51 (dd, *J*(gem) = 9.3 Hz, *J*(2′b,3′) = 5.5 Hz, 1H, H-2′b), 1.99 (s, 3H, CH_3_). ^13^C-NMR (125 MHz, DMSO-*d*_6_): *δ* =170.1 (CH_3_-COO), 161.7 (COOH), 140.6 (C-3 or C-4), 133.3 (C-3 or C-4), 132.8 (C-5), 81.0 (N-CH_2_-O), 77.2 (C-3′), 71.8 (C-4′), 70.2 (C-5′), 69.6 (C-2′). HRMS (ESI^–^) *m*/*z*: [M − H]^–^ calcd. for C_11_H_12_O_8_N_3_, 314.0630; found, 314.0625.

*2-((5-Phenyl-1H-tetrazol-1-yl)methoxy)but-3-en-1-yl acetate (***42***) and 2-((5-phenyl-2H-tetrazol-2-yl)methoxy)but-3-en-1-yl acetate (***43***).* Treatment of 5-phenyl-1*H*-tetrazole (**1r**, 292 mg, 2.0 mmol) and 4-vinyl-[1,3]dioxolane (**2m**, 200 mg, 0.2 mL, 2.0 mmol) by synthetic procedure B afforded **42** (150 mg, 26%) and **43** (121 mg, 21%) as white amorphous solids. Compound **42**: ^1^H-NMR (500 MHz, DMSO-d_6_): δ = 7.95 (m, 2H, H-2), 7.64–7.70 (m, 3H, H-3 and H-4), 5.92 and 5.85 (2×d, J(gem) = 11.9 Hz, 2×1H, N-CH_2_-O), 5.75 (ddd, J(CH,CH_2_) = 17.7 Hz and 10.2 Hz, J(CH,CH) = 6.7 Hz, 1H, CH_2_ = CH), 5.29–5.33 (m, 2H, CH_2_=CH), 4.29 (m, 1H,CH-O), 3.97–4.04 (m, 2H, CH_2_-O), 1.80 (s, 3H, CH_3_); ^13^C-NMR (125 MHz, DMSO-d_6_): δ = 170.3 (COO), 155.2 (N-C-N), 133.2 (CH_2_ = CH), 132.1 (C-4), 129.8 (C-3), 129.3 (C-2), 123.8 (C-1), 120.4 (CH_2_= CH), 77.8 (CH-O), 75.0 (N-CH_2_-O), 65.2 (CH_2_-O), 20.8 (CH_3_). HRMS (ESI^+^) m/z: [M + Na]^+^ calcd. for C_14_H_16_O_3_N_4_Na, 311.1115; found, 311.1113. Compound **43**: ^1^H-NMR (500 MHz, DMSO-d_6_): δ = 8.10 (m, 2H, H-2), 7.55–7.61 (m, 3H, H-3 and H-4), 6.16 and 6.01 (2×d, J(gem) = 11.4 Hz, 2×1H, N-CH_2_-O), 5.75 (ddd, J(CH_2,_CH) = 17.1 Hz and 10.5 Hz, J(CH,CH) = 6.5 Hz, 1H, CH_2_ = CH), 5.35 (dt, J(CH_2_b_,_CH) = 17.3 Hz, J(gem) = ^4^J(H,H) = 1.4 Hz, 1H, CH_2_b = CH), 5.27 (dt, J(CH_2_a_,_CH) = 10.5 Hz, J(gem) = ^4^J(H,H) = 1.4 Hz, 1H, CH_2_a = CH), 4.39 (m, 1H, CH-O), 4.05 (dd, J(gem) = 11.8 Hz, J(CH_2,_CH) = 3.6 Hz, 1H, COO-CH_2_b), 3.98 (dd, J(gem) = 11.8 Hz, J(CH_2_,CH) = 7.2 Hz, 1H, COO-CH_2_a), 1.85 (s, 3H, CH_3_); ^13^C-NMR (125 MHz, DMSO-d_6_): δ = 170.1 (COO), 164.7 (N-C-N), 133.2 (CH_2_=CH), 131.0 (C-4), 129.5 (C-3), 126.8 (C-1), 126.7 (C-2), 119.7 (CH_2_= CH), 79.3 (N-CH_2_-O), 78.3 (CH-O), 65.0 (COO-CH_2_), 20.6 (CH_3_). HRMS (ESI^+^) m/z: [M + Na]^+^ calcd. for C_14_H_16_O_3_N_4_Na, 311.1115; found, 311.1115.

*4-(1H-Benzo[d][1,2,3]triazol-1-yl)-4-methoxybutanenitrile (***45***).* Treatment of benzotriazole (**1t**, 240 mg, 2.0 mmol) and 3-cyanopropionaldehyde dimethyl acetal (**2w**, 260 mg, 0.26 mL, 2.0 mmol) by synthetic procedure B afforded **45** (330 mg, 76%) as a brownish oil. ^1^H-NMR (500 MHz, DMSO-*d*_6_): *δ* = 8.11 (dt, *J*(3′,4′) = 8.4 Hz, *J*(3′,5′) = *J*(3′,6′) = 1.0 Hz, 1H, H-3′), 7.95 (dt, *J*(6′,5′) = 8.4 Hz, *J*(6′,4′) = *J*(6′,3′) = 1.0 Hz, 1H, H-6′), 7.60 (ddd, *J*(5′,6′) = 8.2 Hz, *J*(5′,4′) = 5.9 Hz, *J*(5′,3′) = 1.1 Hz, 1H, H-5′), 7.46 (ddd, *J*(4′,3′) = 8.4 Hz, *J*(4′,5′) = 6.9 Hz, *J*(4′,6′) = 1.0 Hz, 1H, H-4′), 6.16 (m, 1H, H-4), 3.15 (s, 3H, OCH_3_), 2.60–2.69 (m, 3H, H-2 and H-3a), 2.40–2.48 (m, 1H, H-3b). ^13^C-NMR (125 MHz, DMSO-*d*_6_): *δ* = 145.9 (C-2′), 132.0 (C-1′), 128.2 (C-5′), 124.7 (C-4′), 119.8 (C-3′), 119.7 (CN), 111.3 (C-6′), 88.9 (C-4), 56.3 (OCH_3_), 29.6 (C-3), 12.9 (C-2). HRMS (ESI^+^) *m*/*z*: [M + Na]^+^ calcd. for C_11_H_12_ON_4_Na, 239.0903; found, 239.0904.

*2-(2-Bromo-1-(6-chloro-9H-purin-9-yl)ethoxy)ethyl pivalate (***46***).* Treatment of 6-chloropurine (**1a**, 310 mg, 2.0 mmol), 2-bromomethyl-1,3-dioxolane (**2a**, 334 mg, 0.21 mL, 2.0 mmol) and pivalic anhydride (**3b**, 372 mg, 0.41 mL, 2.0 mmol) by synthetic procedure B afforded **46** (414 mg, 51%) as yellowish viscous oil. ^1^H-NMR (500 MHz, DMSO-*d*_6_): *δ* = 8.93 (s, 1H, 8), 8.84 (s, 1H, H-2), 6.13 (dd, *J*(1′,2′a) = 6.9 Hz, *J*(1′,2′b) = 6.0 Hz, 1H, H-1′), 4.23 (dd, *J*(gem) = 10.8 Hz, *J*(2′a,1′) = 6.9 Hz, 1H, H-2′a), 4.16 (dd, *J*(gem) = 10.8 Hz, *J*(2′b,1′) = 6.0 Hz, 1H, H-2′b), 4.04–4.14 (m, 2H, CH_2_-O-CO), 3.86 (ddd, *J*(gem) = 11.5 Hz, *J*(CH_2_a,CH_2_) = 6.1 Hz and 2.8 Hz, 1H, 1′-O-CH_2_a), 3.63 (ddd, *J*(gem) = 11.6 Hz, *J*(CH_2_b,CH_2_) = 6.3 Hz and 2.9 Hz, 1H, 1′-O-CH_2_b), 1.03 (s, 9H, CH_3_). ^13^C-NMR (125 MHz, DMSO-*d*_6_): *δ* = 177.4 (COO), 152.2 (C-2), 152.0 (C-4), 149.7 (C-6), 145.8 (C-8), 131.3 (C-5), 84.2 (C-1′), 67.9 (1′-O-CH_2_), 62.7 (CH_2_-O-CO), 31.7 (C-2′), 26.9 (CH_3_). HRMS (ESI^+^) *m*/*z*: [M + H]^+^ calcd. for C_14_H_19_O_3_N_4_BrCl, 405.0324; found, 405.0325.

*2-(2-Bromo-1-(6-chloro-9H-purin-9-yl)ethoxy)ethyl benzoate (***47***).* Treatment of 6-chloropurine (**1a**, 310 mg, 2.0 mmol), 2-bromomethyl-1,3-dioxolane (**2a**, 334 mg, 0.21 mL, 2.0 mmol) and benzoic anhydride (**3c**, 452 mg, 0.38 mL, 2.0 mmol) by synthetic procedure B afforded **47** (332 mg, 39%) as yellowish viscous oil. ^1^H-NMR (500 MHz, DMSO-*d*_6_): *δ* = 8.95 (s, 1H, H-8), 8.79 (s, 1H, H-2), 7.77 (m, 2H, H-2″), 7.65 (m, 1H, H-4″), 7.48 (m, 2H, H-3″), 6.19 (dd, *J*(1′,2′a) = 6.9 Hz, *J*(1′,2′b) = 6.0 Hz, 1H, H-1′), 4.39 (ddd, *J*(gem) = 12.3 Hz, *J*(CH_2_a,CH_2_b) = 6.7 Hz, *J*(CH_2_a,CH_2_a) = 2.6 Hz, 1H, 1′-O-CH_2_-CH_2_a), 4.30 (ddd, *J*(gem) = 12.3 Hz, *J*(CH_2_b,CH_2_a) = 5.9 Hz, *J*(CH_2_b,CH_2_b) = 2.6 Hz, 1H, 1′-O-CH_2_,CH_2_b), 4.24 (dd, *J*(gem) = 10.8 Hz, *J*(2′a,1′) = 6.9 Hz, 1H, H-2′a), 4.03 (dd, *J*(gem) = 10.8 Hz, *J*(2′b,1′) = 6.0 Hz, 1H, H-2′b), 4.03 (ddd, *J*(gem) = 11.7 Hz, *J*(CH_2_a,CH_2_b) = 5.9 Hz, *J*(CH_2_a,CH_2_a) = 2.6 Hz, 1H, 1′-O-CH_2_a), 3.81 (ddd, *J*(gem) = 11.7 Hz, *J*(CH_2_b,CH_2_a) = 6.7 Hz, *J*(CH_2_b,CH_2_b) = 2.6 Hz, 1H, 1′-O-CH_2_b). ^13^C-NMR (125 MHz, DMSO-*d*_6_): *δ* = 165.7 (COO), 152.2 (C-2), 151.9 (C-4), 149.6 (C-6), 145.8 (C-8), 133.6 (C-4″), 131.3 (C-5), 129.5 (C-1″), 129.2 (C-2″), 128.9 (C-3″), 84.5 (C-1′), 68.0 (1′-O-CH_2_), 63.6 (1′-O-CH_2_-CH_2_), 31.7 (C-2’). HRMS (ESI^+^) *m*/*z*: [M + Na]^+^ calcd. for C_16_H_14_O_3_N_4_BrClNa, 446.9830; found, 446.9833.

*2-(2-Bromo-1-(6-chloro-9H-purin-9-yl)ethoxy)ethyl(2S)-2-(6-methoxynaphthalen-2-yl)propanoate (***49***).* Treatment of 6-chloropurine (**1a**, 310 mg, 2.0 mmol), 2-bromomethyl-1,3-dioxolane (**2a**, 334 mg, 0.21 mL, 2.0 mmol) and (*S*,*S*)-2-(6-methoxynaphthalene-2-yl)propionic anhydride (**3e**, 890 mg, 2.0 mmol) by synthetic procedure B afforded **49** (214 mg, 20%) as yellowish viscous oil. ^1^H-NMR (500 MHz, DMSO-*d*_6_): *δ* = 8.87 and 8.91 (s, 1H, H-8), 8.83 and 8.83 (s, 1H, H-2), 7.73–7.80 (m, 2H, H-4″ and H-8″), 7.64–7.67 (m, 1H, H-1″), 7.30–7.34 (m, 1H, H-3″), 7.26–7.28 (m, 1H, H-5″), 7.13–7.16 (m, 1H, H-7″), 6.06–6.10 (m, 1H, H-1″), 3.98–4.21 (m, 4H, CH_2_Br, COO-CH_2_), 3.58–3.85 (m, 3H, CH_3_-CH and 1′-O-CH_2_), 1.41 and 1.40 (2 × d, *J*(CH_3_,CH) = 7.1 Hz, 2 × 3H, CH_3_). ^13^C-NMR (125 MHz, DMSO-*d*_6_) *δ* = 173.9 (COO), 157.4 (C-6″), 152.3 (C-2), 152.00 and 151.98 (C-4), 149.7 (C-6), 145.8 and 145.7 (C-8), 135.64 and 135.56 (C-2″), 133.5 (C-4″a), 131.3 (C-5), 129.3 (C-8″), 128.58 and 128.56 (C-8″a), 127.20 and 127.17 (C-4″), 126.4 and 126.3 (C-3″), 125.82 and 125.77 (C-1″), 119.0 (C-7″), 105.9 (C-5″), 84.2 and 84.1 (C-1′), 67.7 (1′-O-CH_2_), 63.2 and 63.1 (COO-CH_2_), 55.4 (O-CH_3_), 44.6 and 44.5 (CH_3_-CH), 31.6 (CH_2_Br), 18.6 and 18.5 (CH_3_). HRMS (ESI^+^) *m*/*z*: [M + H]^+^ calcd. for C_23_H_23_O_4_N_4_BrCl, 533.0586; found, 533.0587.

*2-(2-Bromo-1-(6-chloro-9H-purin-9-yl)ethoxy)ethyl decanoate (***51***).* Treatment of 6-chloropurine (**1a**, 310 mg, 2.0 mmol), 2-bromomethyl-1,3-dioxolane (**2a**, 334 mg, 0.21 mL, 2.0 mmol) and decanoyl chloride (**3h**, 381 mg, 0.42 mL, 2.0 mmol) by synthetic procedure B afforded **51** (332 mg, 35%) as yellow viscous oil. ^1^H-NMR (500 MHz, DMSO-*d*_6_): *δ* = 8.93 (s, 1H, 8), 8.83 (s, 1H, H-2), 6.13 (dd, *J*(1′,2′a) = 7.0 Hz, *J*(1′,2′b) = 5.8 Hz, 1H, H-1′), 4.23 (dd, *J*(gem) = 10.8 Hz, *J*(2′a,1′) = 7.0 Hz, 1H, H-2′a), 4.14 (dd, *J*(gem) = 10.8 Hz, *J*(2′b,1′) = 5.8 Hz, 1H, H-2′b), 4.09 (ddd, *J*(gem) = 12.4 Hz, *J*(CH_2_a,CH_2_b) = 6.6 Hz, *J*(CH_2_a,CH_2_a) = 2.8 Hz, 1H, 1′-O-CH_2_-CH_2_a), 4.04 (ddd, *J*(gem) = 12.4 Hz, *J*(CH_2_b,CH_2_a) = 5.7 Hz, *J*(CH_2_b,CH_2_b) = 2.9 Hz, 1H, 1′-O-CH_2_-CH_2_b), 3.88 (ddd, *J*(gem) = 11.7 Hz, *J*(CH_2_a,CH_2_b) = 5.7 Hz, *J*(CH_2_a,CH_2_a) = 2.8 Hz, 1H, 1′-O-CH_2_a), 3.64 (ddd, *J*(gem) = 11.7 Hz, *J*(CH_2_b,CH_2_a) = 6.6 Hz, *J*(CH_2_b,CH_2_b) = 2.9 Hz, 1H, 1′-O-CH_2_b), 2.08 (m, 2H, H-2″), 1.15–1.28 (m, 12H, H-4″, H-5″, H-6″, H-7″, H-8″, H-9″), 1.38 (m, 2H, H-3″), 0.84 (t, *J*(10″,9″) = 7.0 Hz, 3H, H-10″), ^13^C-NMR (125 MHz, DMSO-*d*_6_): *δ* =172.8 (C-1″), 152.2 (C-2), 152.0 (C-4), 149.6 (C-6), 145.8 (C-8), 131.3 (C-5), 84.3 (C-1′), 68.0 (1′-O-CH_2_), 62.6 (1′-O-CH_2_-CH_2_), 33.4 (C-2″), 31.7 (C-2′), 31.5 (C-8″), 29.0, 28.9 and 28.6 (C-4″, C-5″, C-6″, C-7″), 24.5 (C-3″), 22.3 (C-9″), 14.2 (C-10″). HRMS (ESI^+^) *m*/*z*: [M + H]^+^ calcd. for C_19_H_29_O_3_N_4_BrCl, 475.1106; found, 475.1109.

*2-(1-Acetoxy-2-bromoethoxy)ethyl acetate (***52***).* A mixture of 2-bromomethyl-1,3-dioxolane (**2a**, 1.00 g, 6.25 mmol), acetic anhydride (**3a**, 0.96 g, 0.89 mL, 9.41 mmol) and concentrated H_2_SO_4_ (1 drop) was stirred at room temperature overnight. Water (30 mL) was added and the mixture was extracted with chloroform (3 × 30 mL). Combined organic layers were washed with brine (30 mL), dried over MgSO_4_, and evaporated to give crude **52** (1.2 g, 71%, GC purity 91%) as brown viscous oil. Purification of the crude product using column chromatography on silica gel (DCM/1% methanol, isocratic) afforded pure **52** (0.94 g, 56%) as brownish oil. ^1^H-NMR (400 MHz, CDCl_3_) δ = 5.94 (t, *J*(CH-CH_2_Br) = 5.3 Hz, 1H, O-CH-O), 4.29–4.16 (m, 2H, CH_2_-OAc), 3.93 (ddd, *J* (gem) = 11.4 Hz, *J*(CH_2_-CH_2_) = 5.3 Hz, *J*(CH_2_-CH_2_) = 3.8 Hz, 1H, O-CH_2_a-CH_2_-OAc), 3.93 (ddd, *J* (gem) = 11.5 Hz, *J*(CH_2_-CH_2_) = 5.9 Hz, *J*(CH_2_-CH_2_) = 4.1 Hz, 1H, O-CH_2_b-CH_2_-OAc), 3.51–3.34 (m, 2H, CH_2_-Br), 2.12 (s, 3H, CH_3_AcO-CH_2_-CH_2_), 2.08 (s, 3H, CH_3_AcO-CH-O). ^13^C-NMR (100 MHz, CDCl_3_): δ = 171.0(COO-CH_2_-CH_2_), 170.5 (COO-CH-O), 96.2 (O-CH-O), 68.3 (O-CH_2_-CH_2_-OAc), 63.2 (O-CH_2_-CH_2_-OAc), 31.1 (CH_2_-Br), 21.1 (CH_3_AcO), 21.0 (CH_3_AcO). HRMS (ESI^+^) *m*/*z*: [M + Na]^+^ calcd. for C_8_H_13_O_5_BrNa, 290.9839; found, 290.9836.

## 4. Conclusions

In summary, we have developed an efficient strategy for the preparation of acylated purine-based acyclonucleosides branched at the hemiaminal ether carbon atom. The three-component reaction takes advantage of cheap and easily available starting material, namely acetals, acetic anhydride and trimethylsilyl trifluoromethanesulfonate (TMSOTf), and of simple procedure (one-pot reaction, room temperature, short reaction time). The general procedure is based on equimolar reaction mixture of *N*-heterocycle, acetal, acetic anhydride, and TMSOTf (1.5 equivalent is preferable) in MeCN and offers the target acyclonucleosides in moderate to high yields (up to 88%). The substrate scope is relatively broad, both as for the *N*-heterocycle and as for the acetal (cyclic or acyclic) and many functional groups are tolerated (e.g., double and triple bonds, halogen, tosyl, cyano, keto, and ester groups). It was shown that starting 1,3-dioxolanes can be branched either at C-2 or at C-4/C-5 positions; however, no reaction was observed with acetals bearing azido group and free hydroxyl or amino groups. The *N*-heterocycles usually react with a great regioselectivity and also the unprotected exocyclic amino group is mostly well-tolerated. Although other acid anhydrides (e.g., pivalic anhydride) can be alternatively used, the best yields were obtained with acetic anhydride.

This efficient, simple and fast alkoxyalkylation methodology offers an efficient approach to diverse protected (acylated) acyclic nucleosides which can serve as convenient intermediates for subsequent synthesis of various target compounds (e.g., acyclonucleosides and acyclic nucleoside phosphonates). The methodology is currently being exploited in our laboratory in order to prepare variously branched acyclonucleosides and acyclic nucleoside phosphonates, which are further studied as potential inhibitors of enzymes of purine metabolism (e.g., adenylate cyclases, purine nucleoside phosphorylases, and 6-oxopurine phosphoribosyltransferase) with the aim to develop potential therapeutic agents.

## Figures and Tables

**Table 1:** Preparation of acyclonucleoside **4** by multi-component reaction—Lewis acid optimization ^a^.

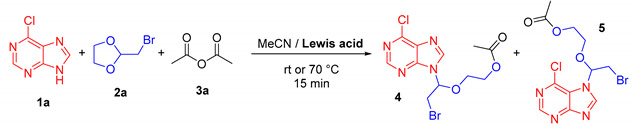		
Lewis Acid	rt	70 °C
	Yield *^b^* (%) of 4/5	Yield *^b^* (%) of 4/5
SnCl_4_	60/-	70/-
TMSOTf	83/-	70/-
FeCl_3_	7/3	38/-
AlCl_3_	11/-	21/-
ZnCl_2_	5/2	0.6/traces ^c^
TiCl_4_	11/traces ^c^	25/5
BF_3_·Et_2_O	30/15	27/9

^a^ General procedure A. ^b^ Isolated yields. ^c^ **5** detected by UPLC-MS, not isolated.

**Table 2:** Preparation of acyclonucleoside **4** by multi-component reaction—solvent optimization ^a^.

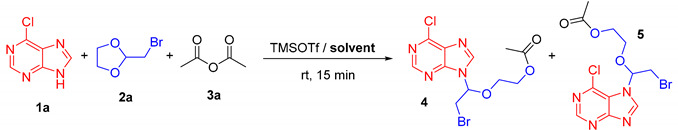			
Solvent	Yield *^b^* (%) of 4/5	Solvent	Yield *^b^* (%) of 4/5
DMSO	-/- ^c^	acetic acid	3/-
DMF	-/-	acetone	14/-
NMP	-/-	DCM	39/-
THF	-/-	nitromethane	62/5
cyclohexane	-/-	EtOAc	56/4
methanol	-/-	dioxane	48/6
pyridine	-/-	MeCN	83/-
toluene	3/-		

^a^ General procedure A in various solvents. ^b^ Isolated yields. ^c^ No reaction.

## Supplementary Materials

The following are available online. Copies of ^1^H and ^13^C NMR spectra of prepared compounds (pages S2–S42). Figures S1 and S2 (pages S43 and S44): HMBC spectra of compounds **22** and **42**.
