# Supplementary material for: Efficient Synthesis of α-Branched Purine-Based Acyclic Nucleosides: Scopes and Limitations of the Method
Source: Molecules. 2020 Sep 19;25(18):4307. doi: 10.3390/molecules25184307 (PMC7571146; doi:10.3390/molecules25184307)
Supplement: Supplementary file 1 [file molecules-25-04307-s001.pdf]

Supporting Information

Efficient Synthesis of α-Branched Purine-Based Acyclic Nucleosides: Scopes and Limitations of the Method

*Jan Frydrych, Lenka Poštová Slavětínská, Martin Dračínský, Zlatko Janeba**

Institute of Organic Chemistry and Biochemistry of the Czech Academy of Sciences, Flemingovo nám. 2, CZ-16610, Prague 6, Czech Republic

e-mail: janeba@uochb.cas.cz

1. Copies of ^{1}H and ^{13}C NMR spectra …………………………………………..…. S2-S42

2. HMBC spectra of compounds 22 and 42 ………..……………………..… S43-S44

2-(1,2-Dihydroxyethyl)-1,3-dioxolane-1,3-diacetate (**2b**)

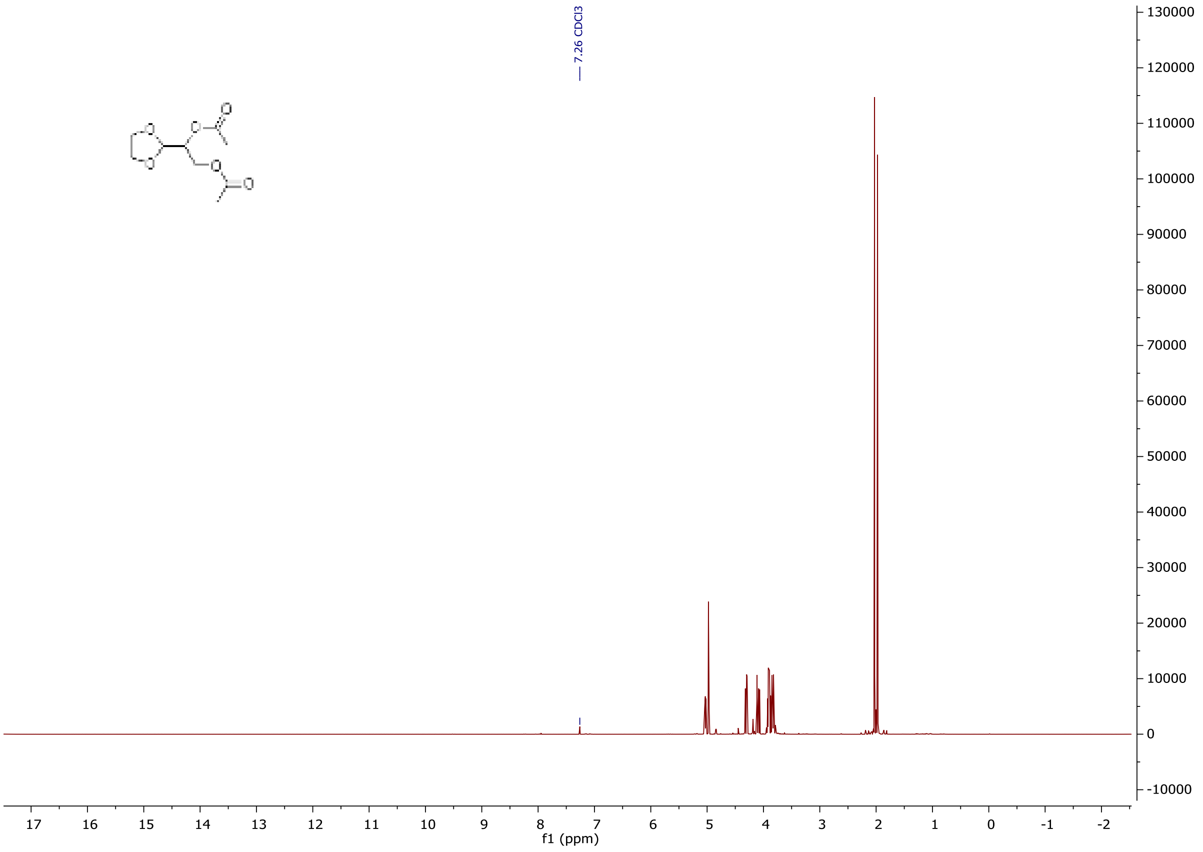

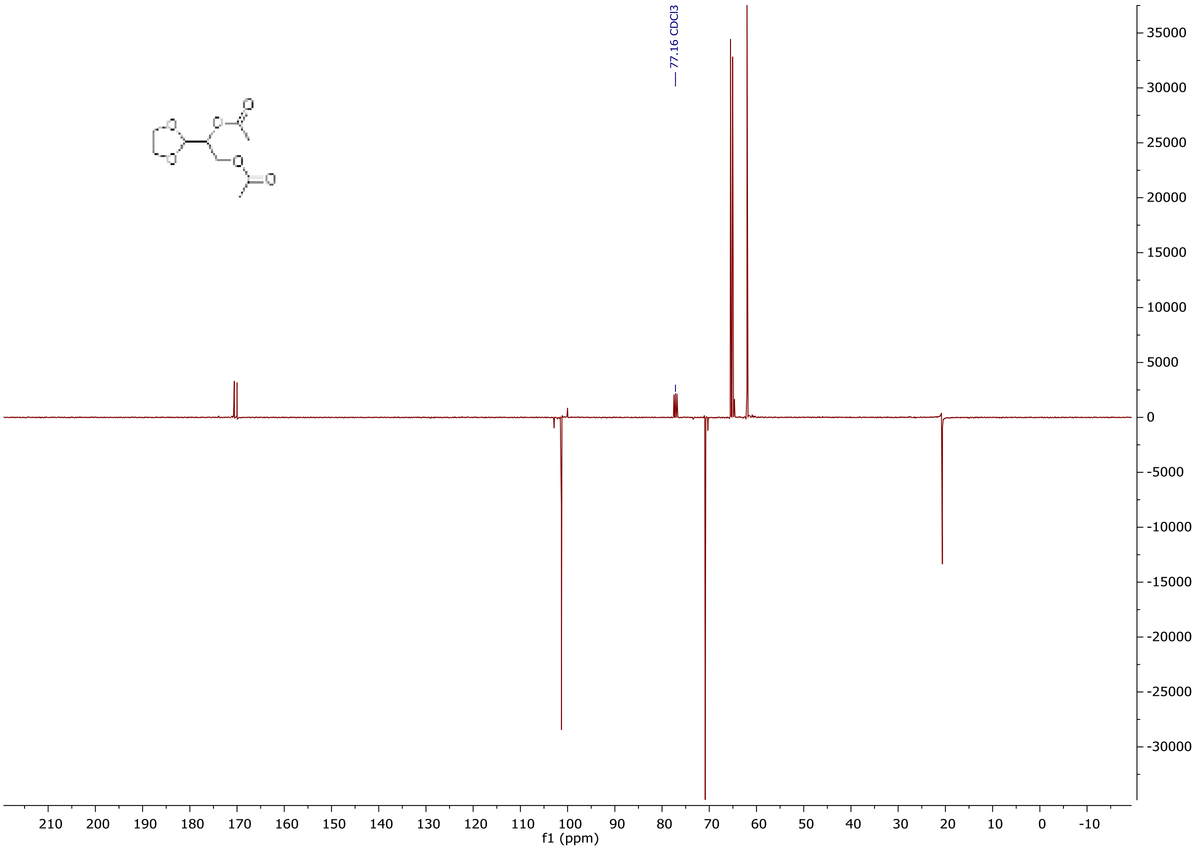

Phenylmethyl *syn*-tetrahydro-5*H*-[1,3]dioxolo[4,5-*c*]pyrrole-5-carboxylate (**2k**)

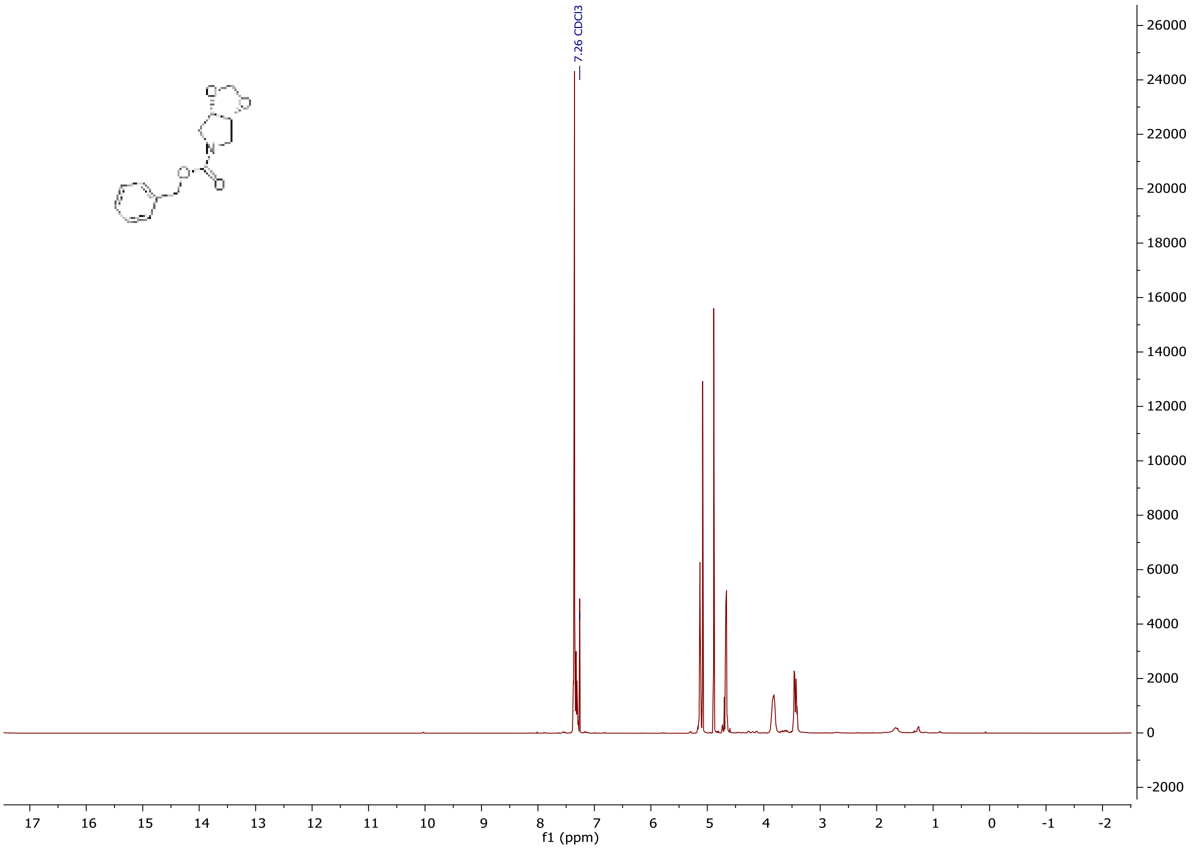

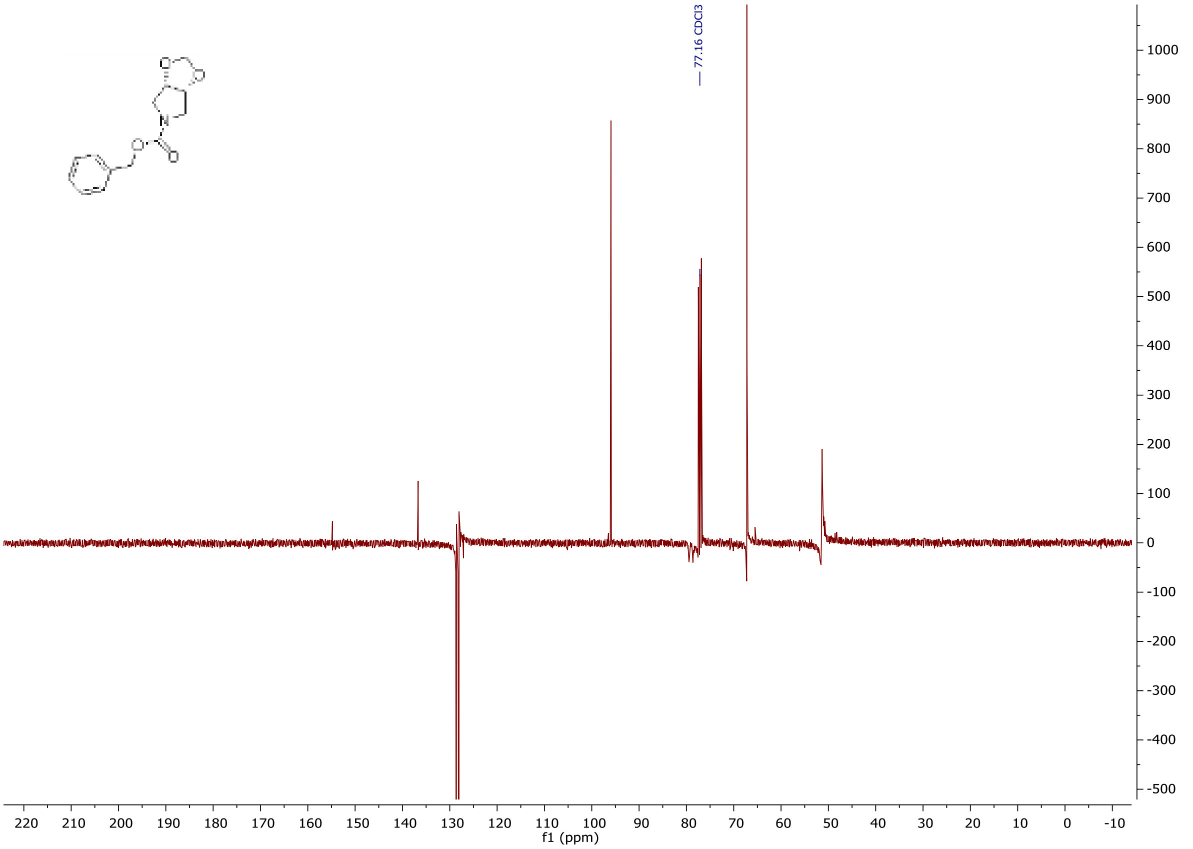

4-(Azidomethyl)-1,3-dioxolane (**2n**)

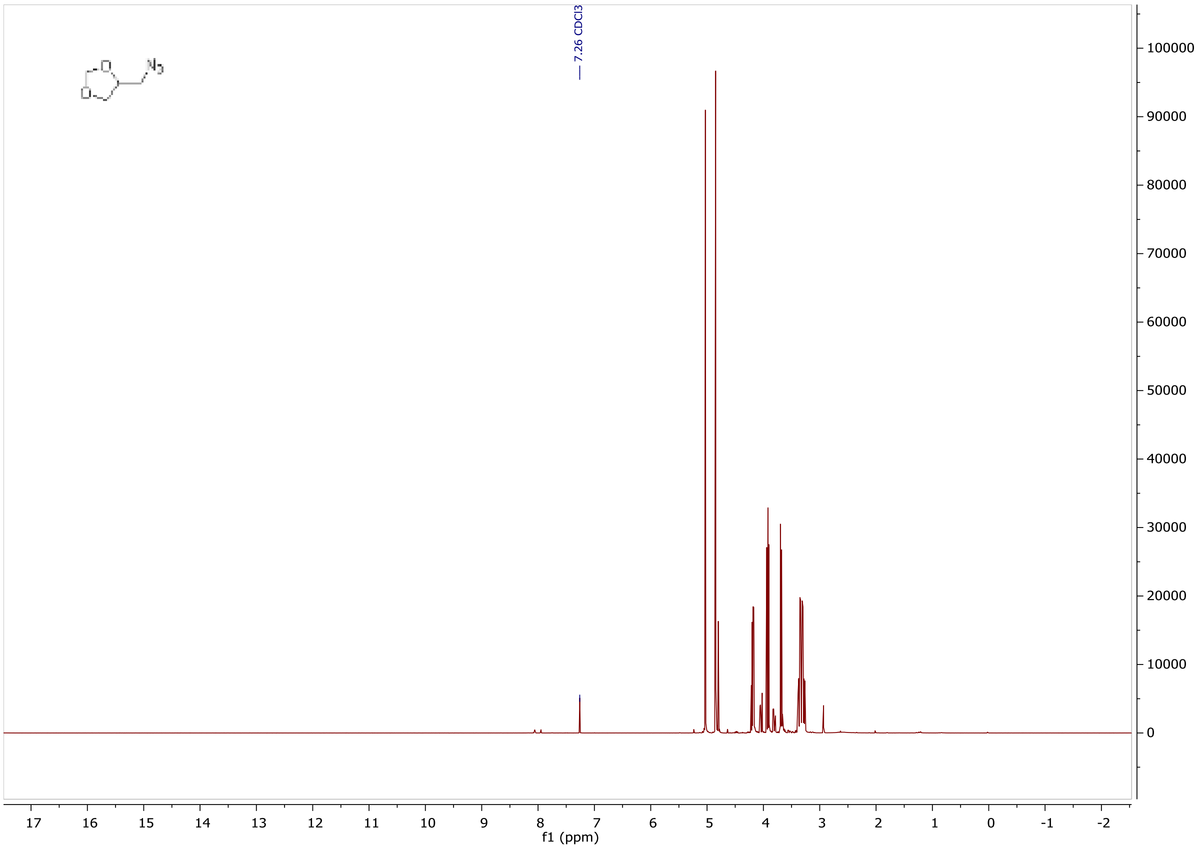

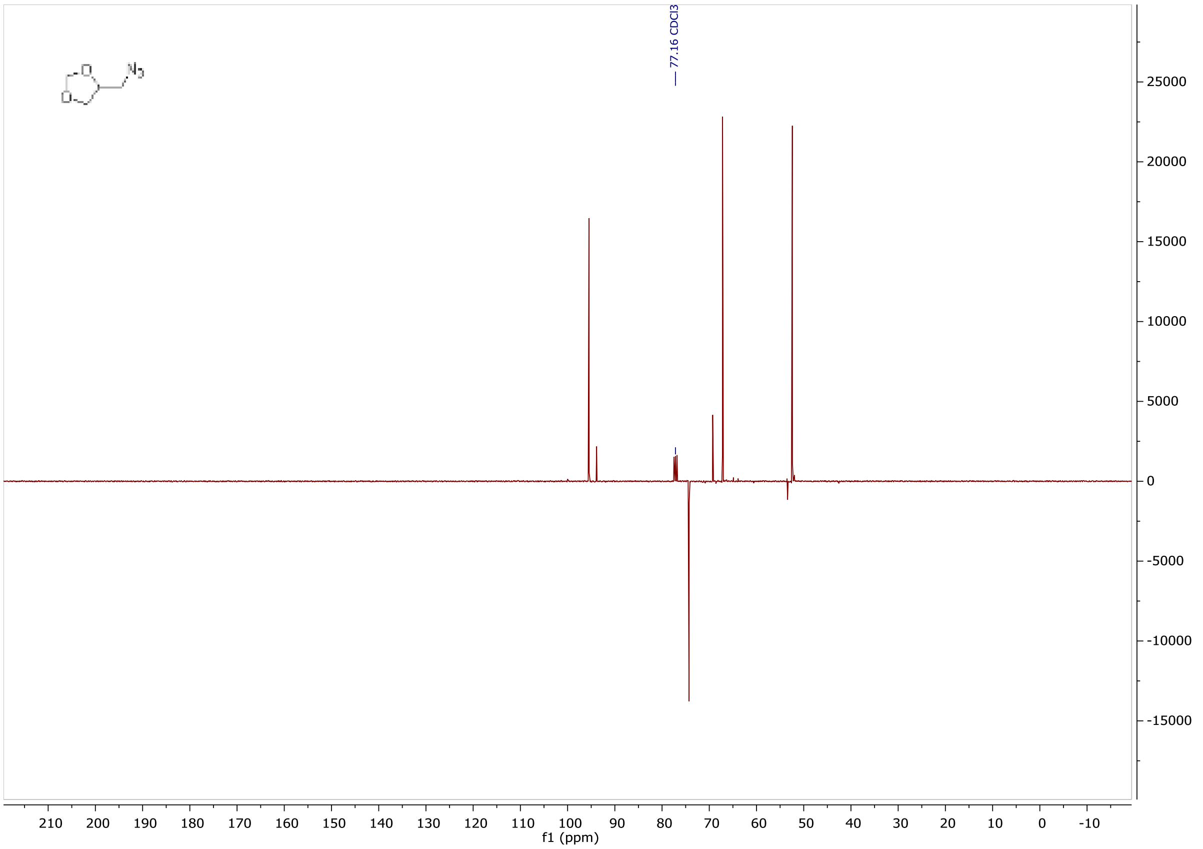

2,2-Diethoxyethan-1-ol (**2q**)

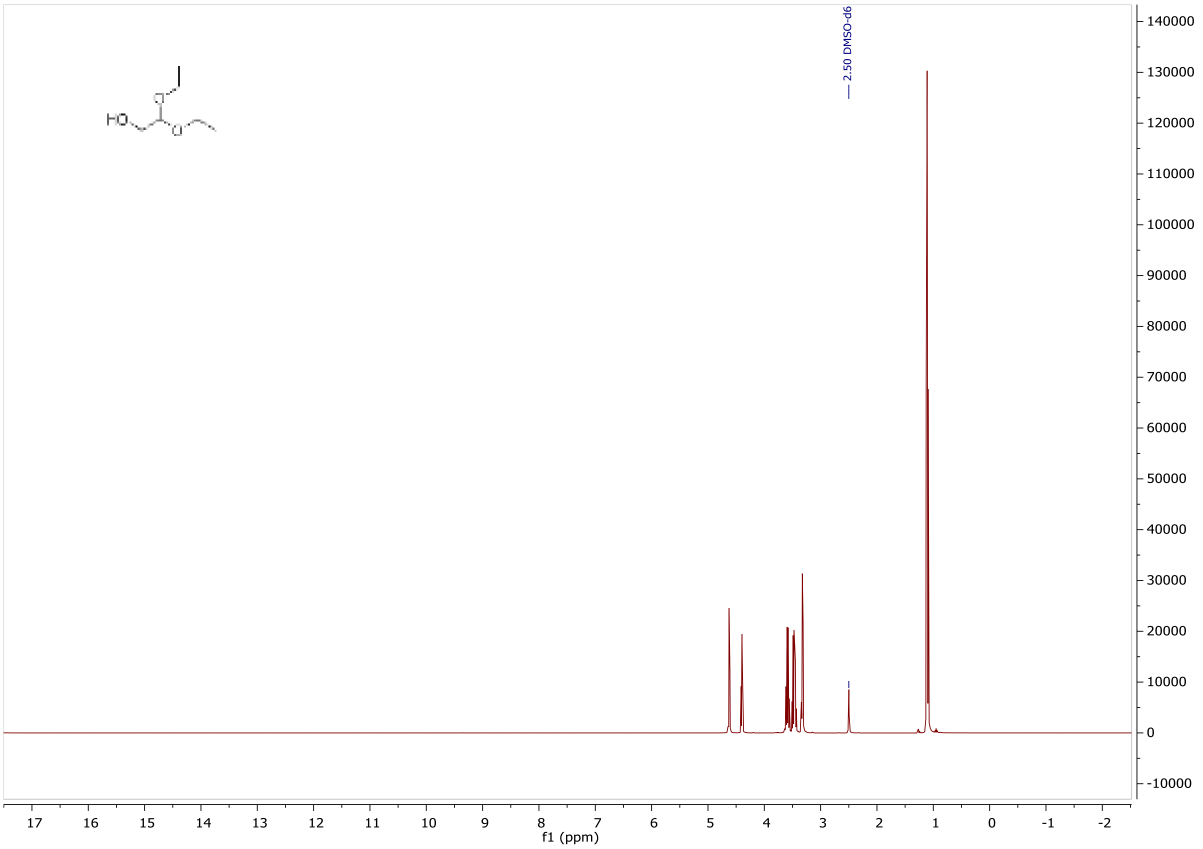

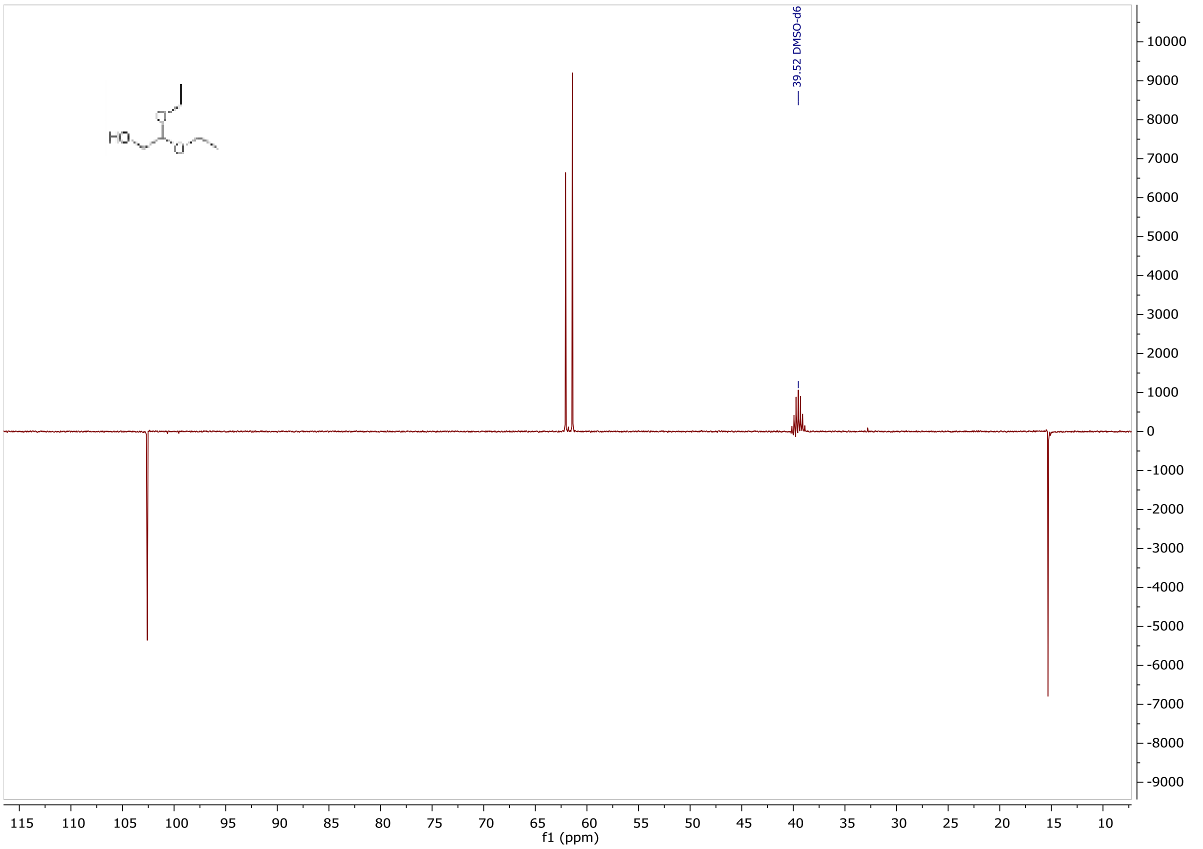

2,2-Diethoxyethyl acetate (**2t**)

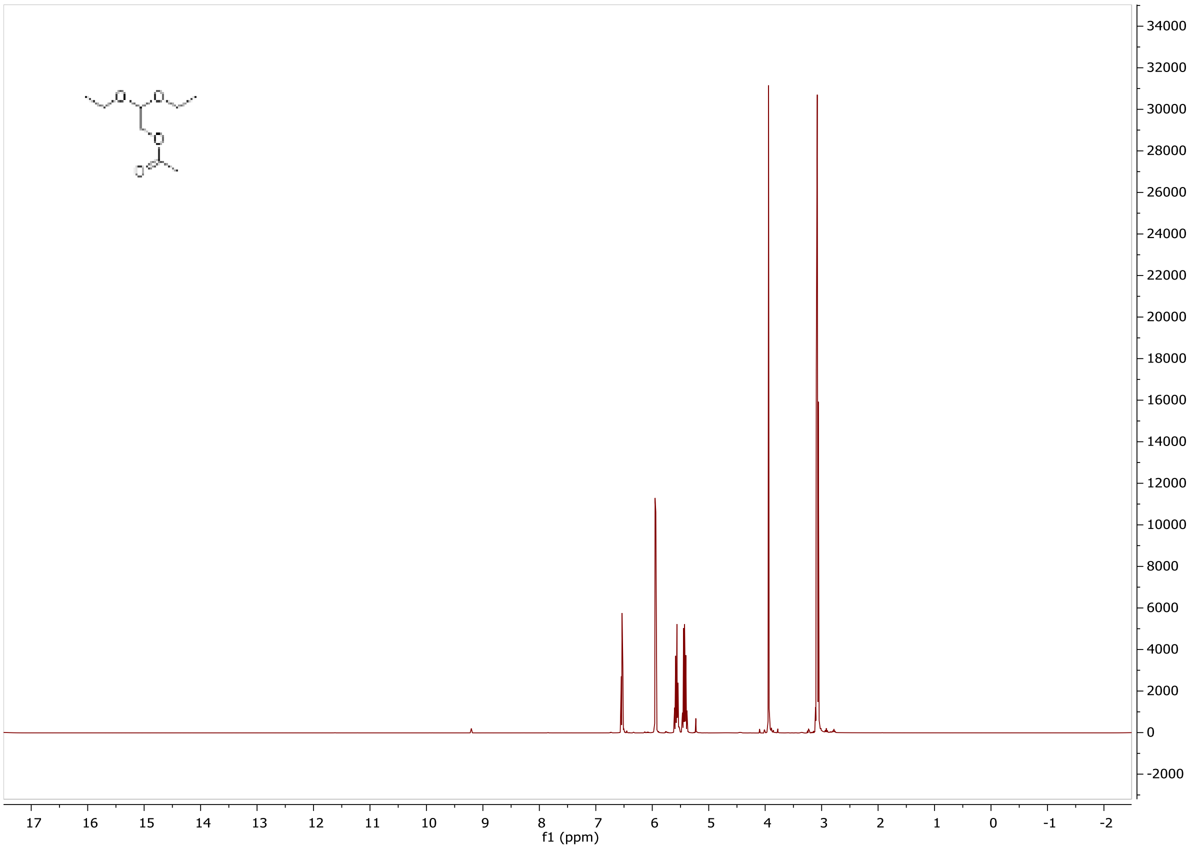

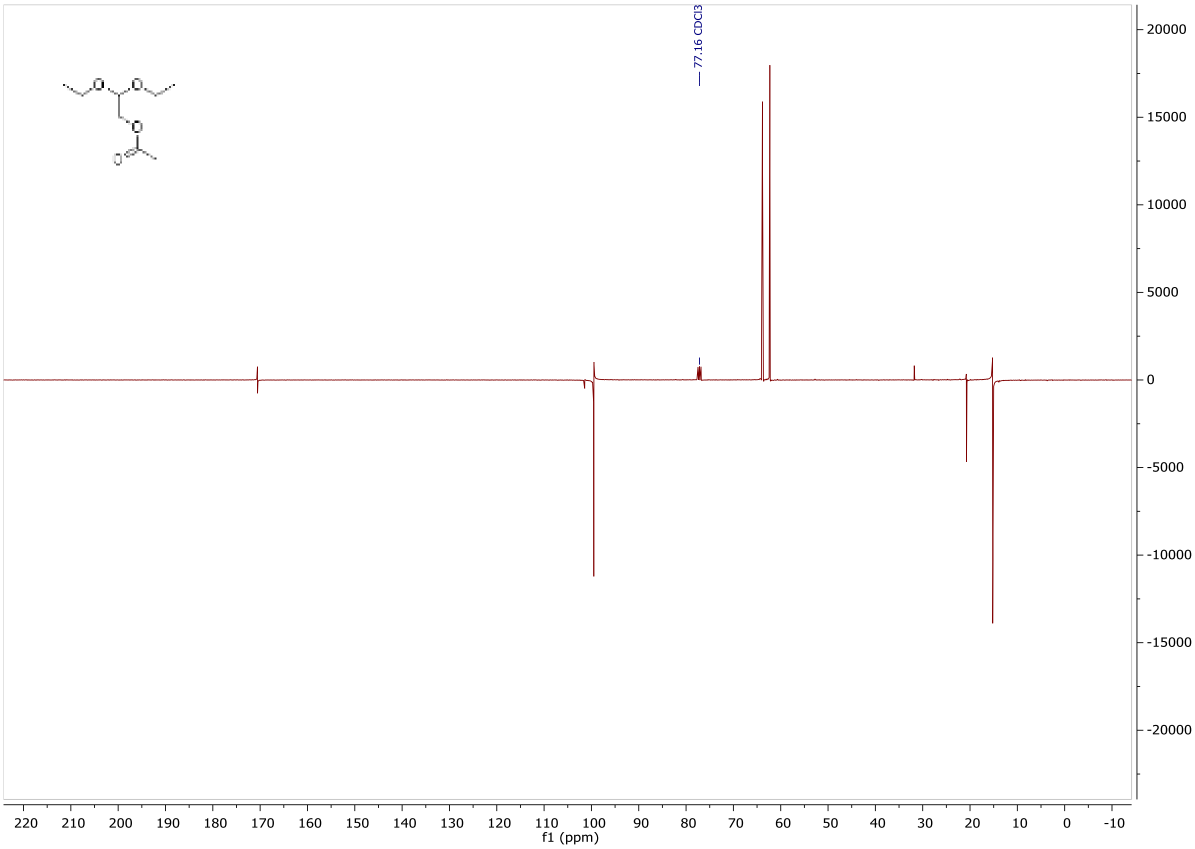

Tetrahydrofuro[3,4-*d*][1,3]dioxole (**2v**)

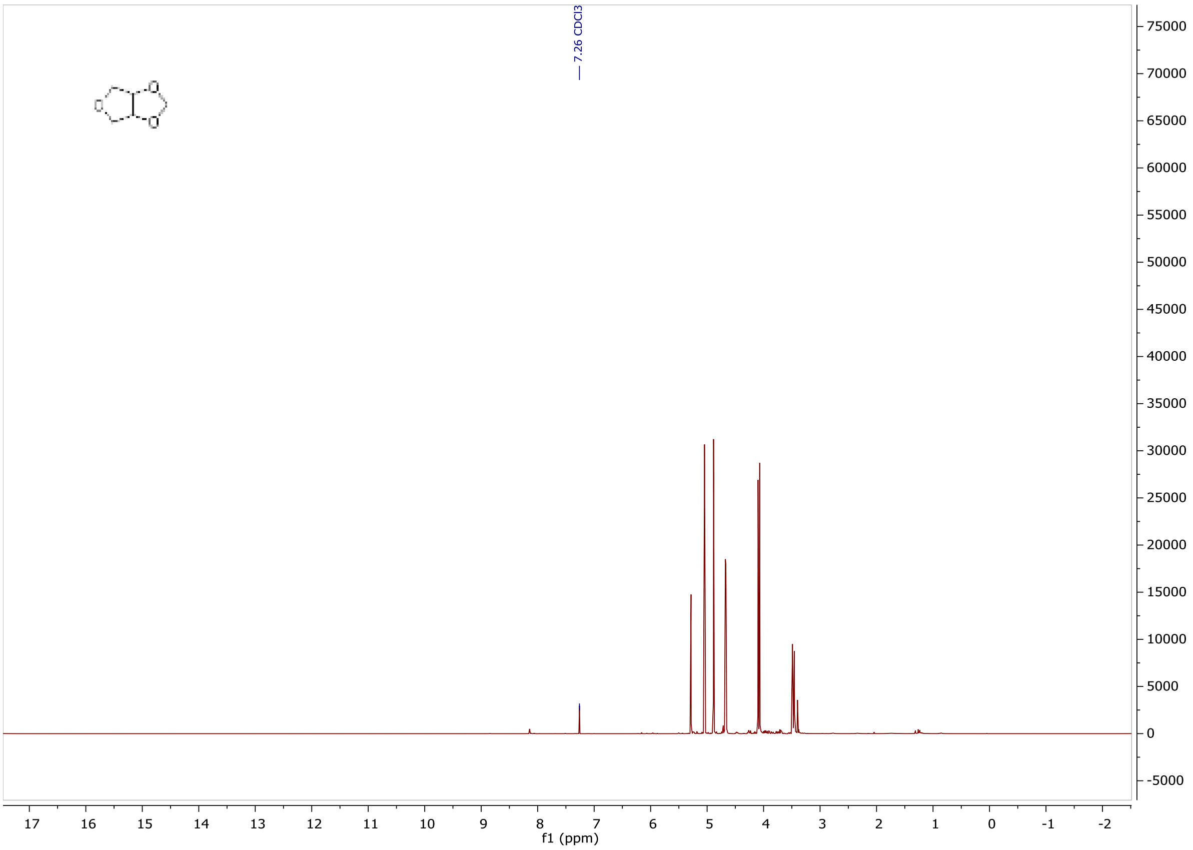

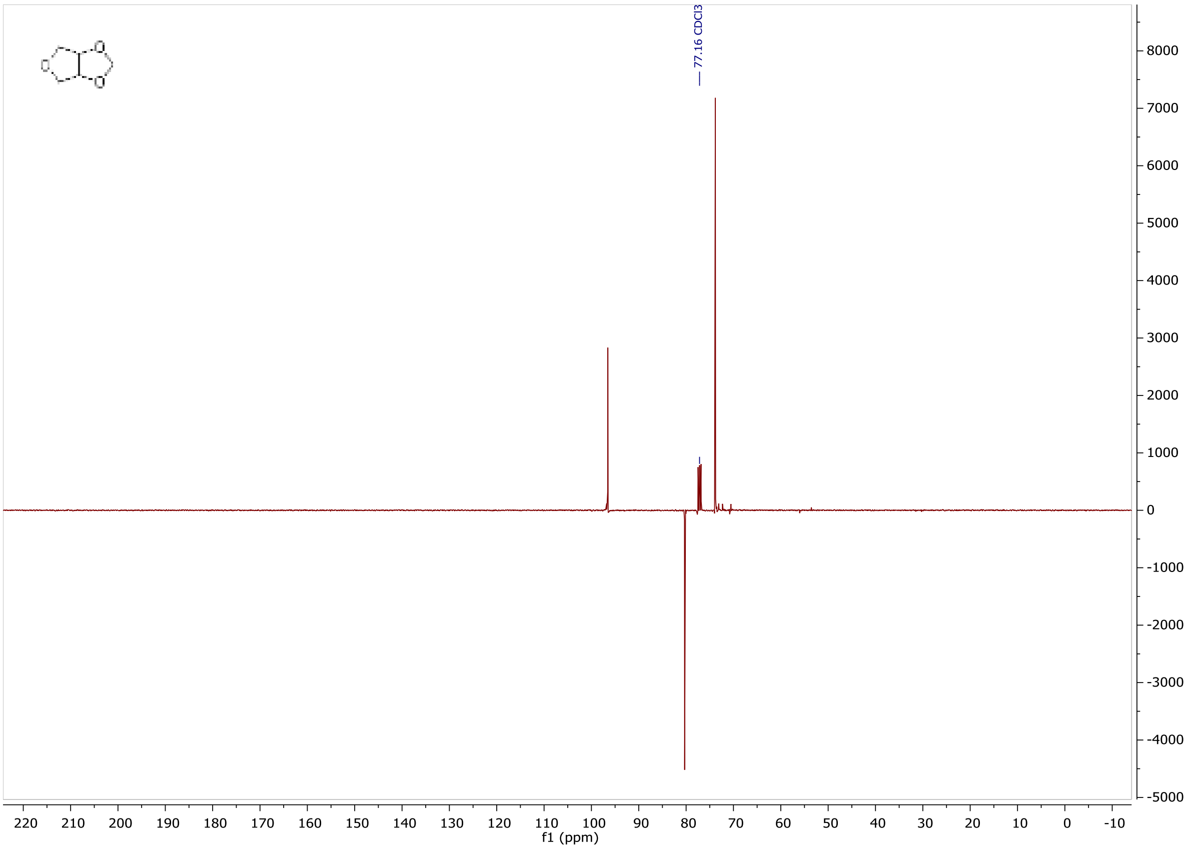

(*S*,*S*)-2-(6-Methoxynaphthalene-2-yl)propionic anhydride (**3e**)

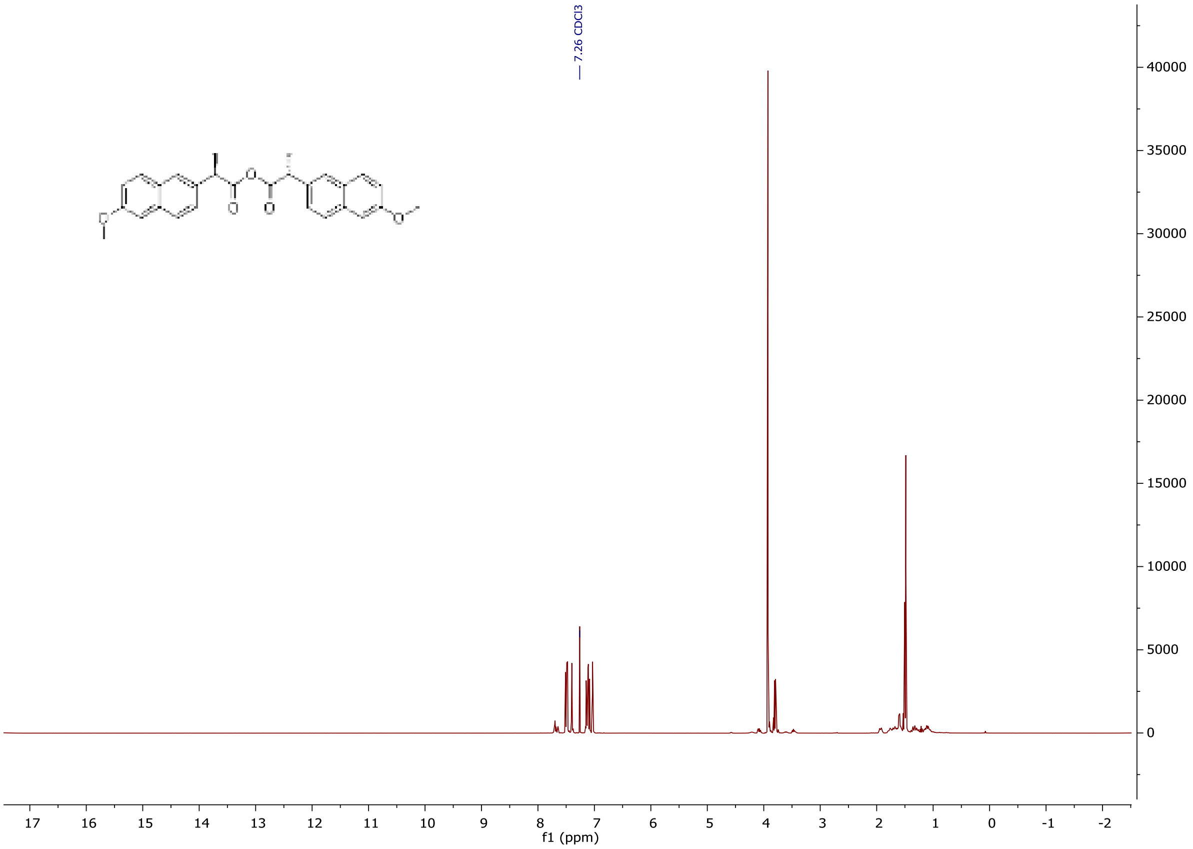

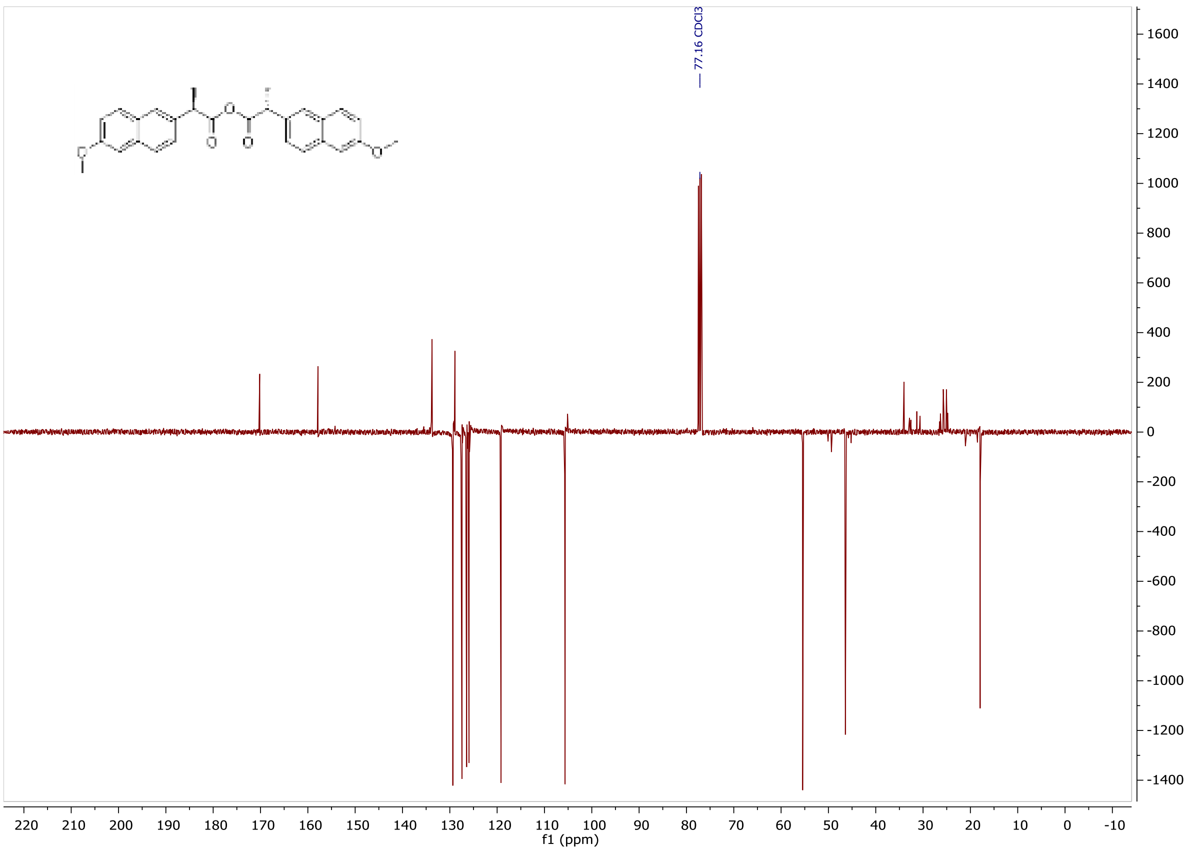

2-(2-Bromo-1-(6-chloro-9*H*-purin-7-yl)ethoxy)ethyl acetate (**4**)

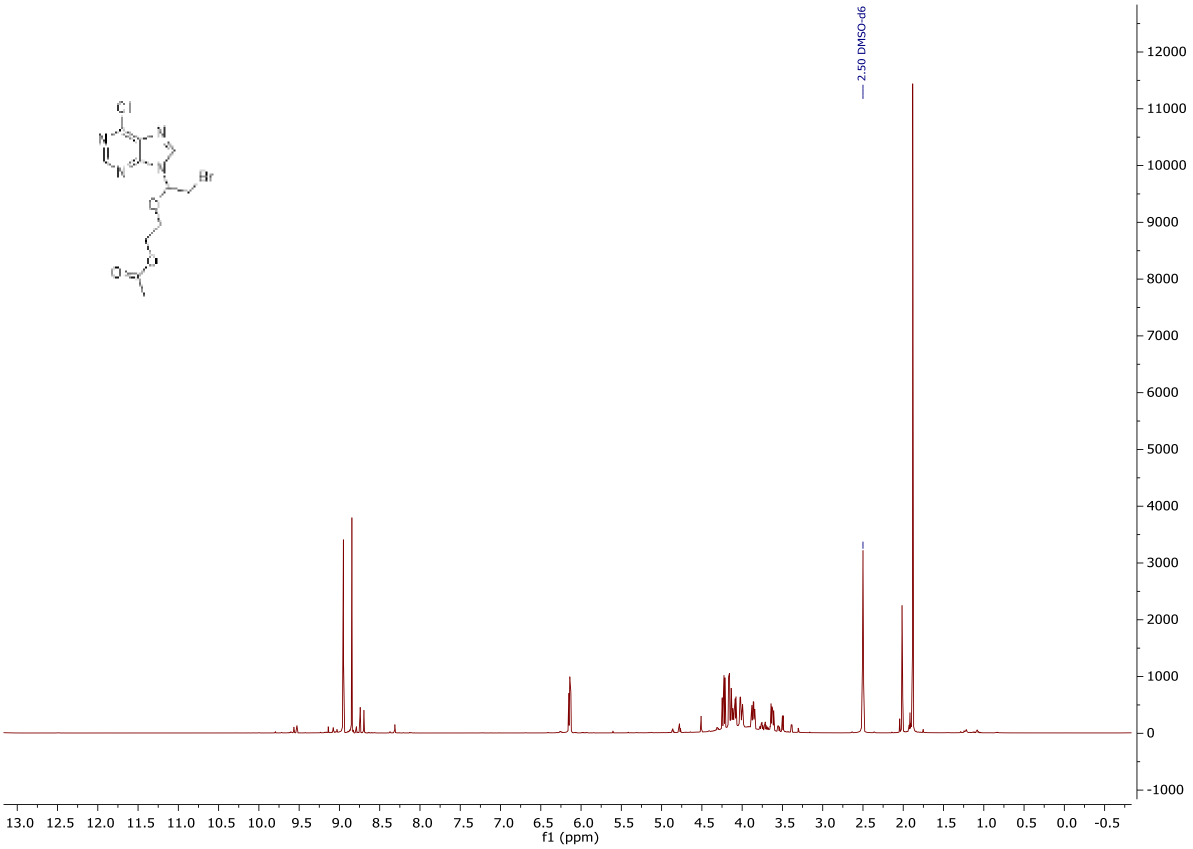

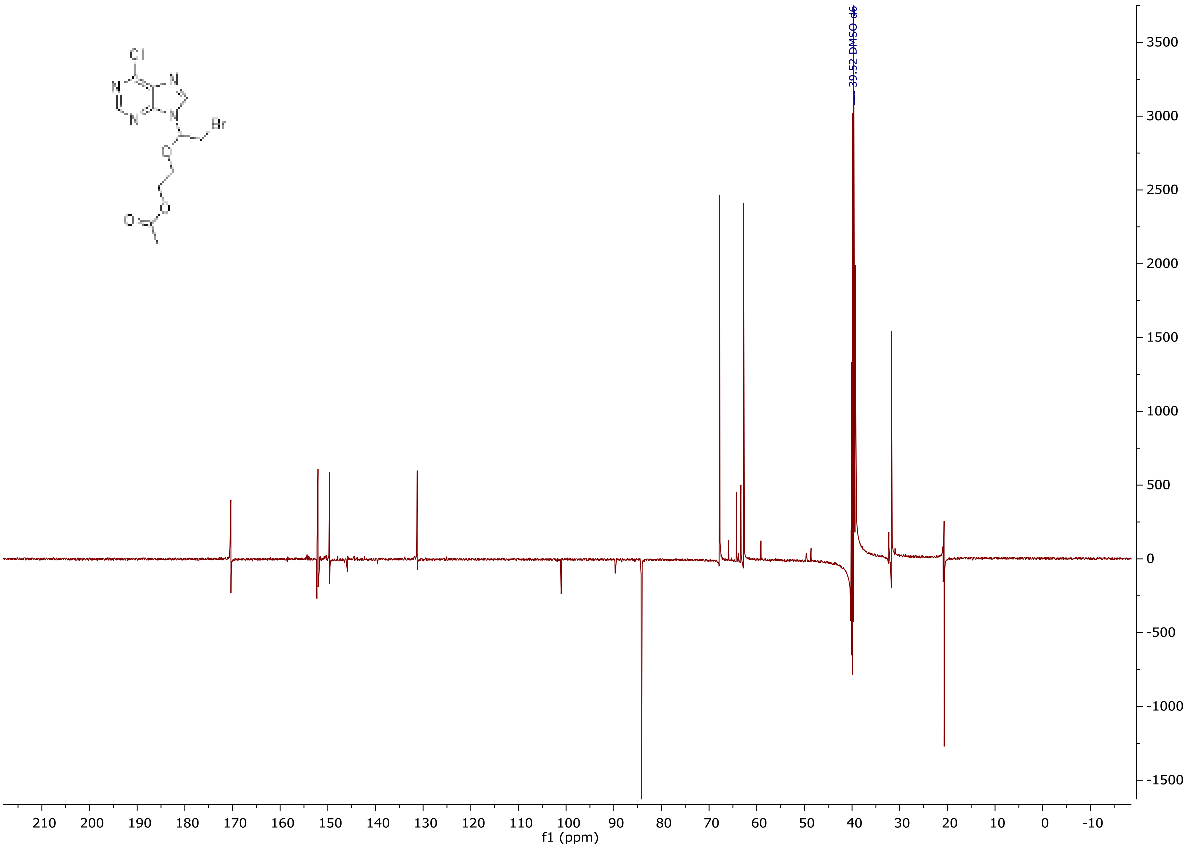

2-(2-Bromo-1-(6-chloro-7*H*-purin-7-yl)ethoxy)ethyl acetate (**5**)

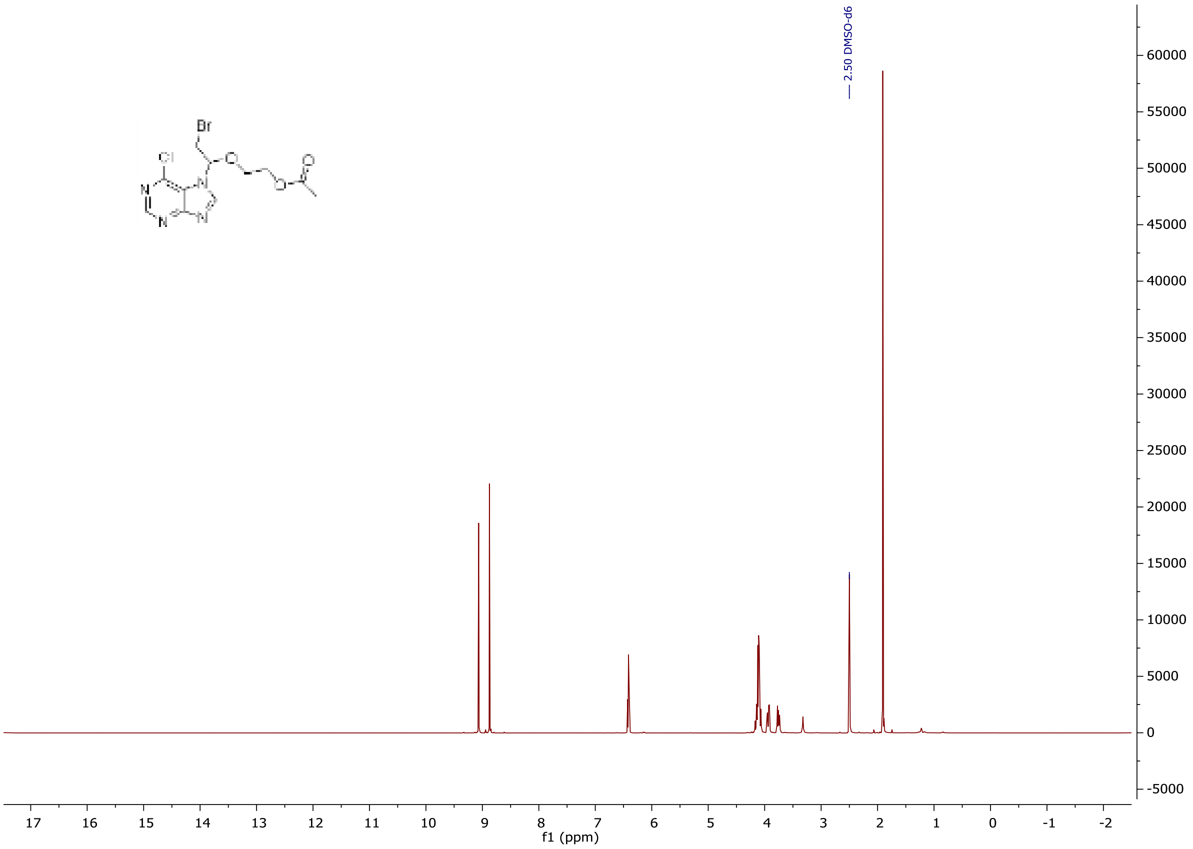

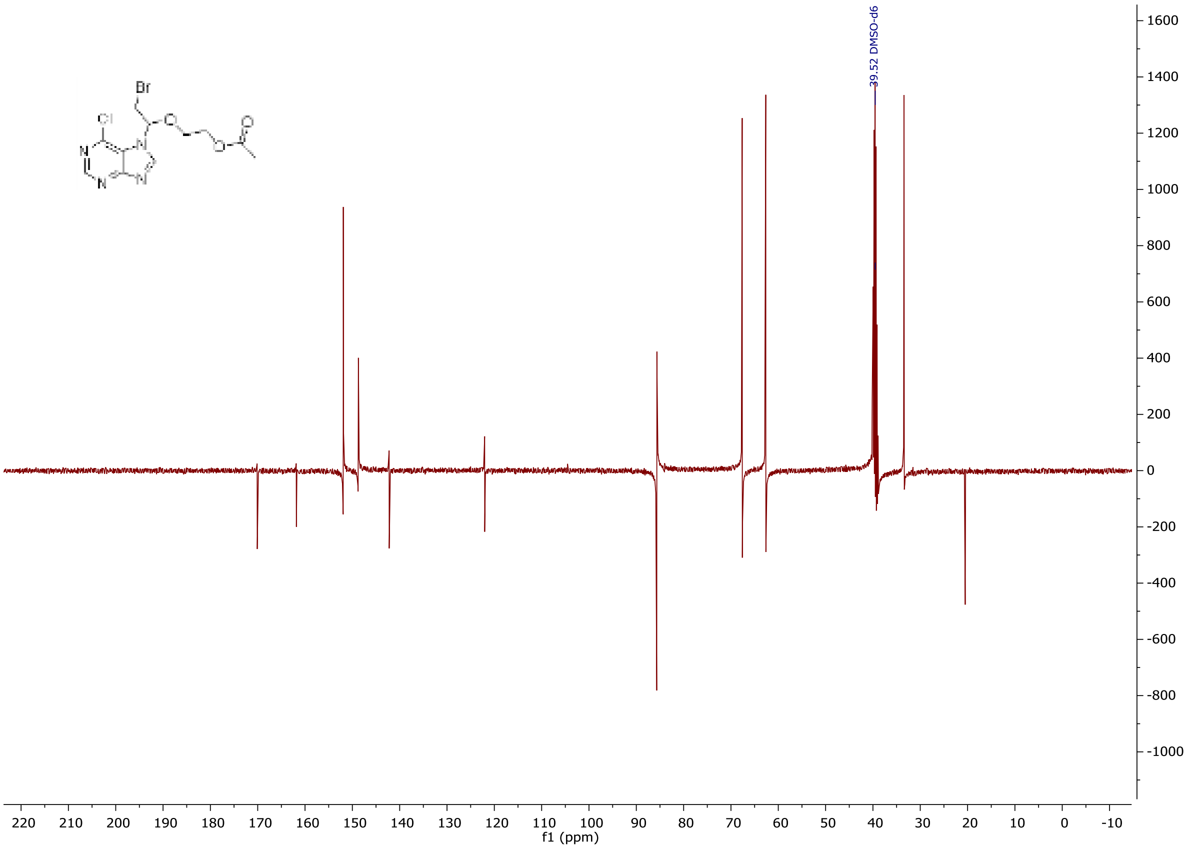

3-(2-Acetoxyethoxy)-3-(6-chloro-9*H*-purin-9-yl)propane-1,2-diyl diacetate (**6**)

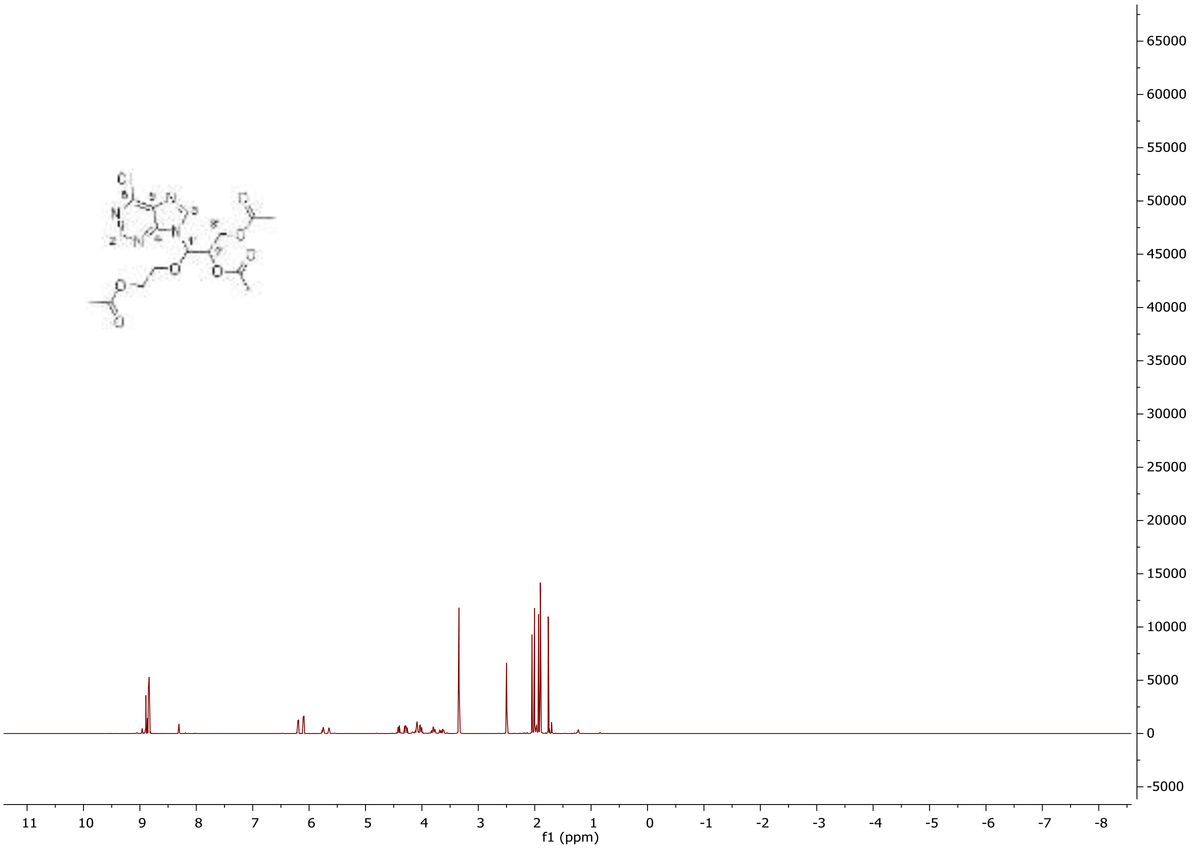

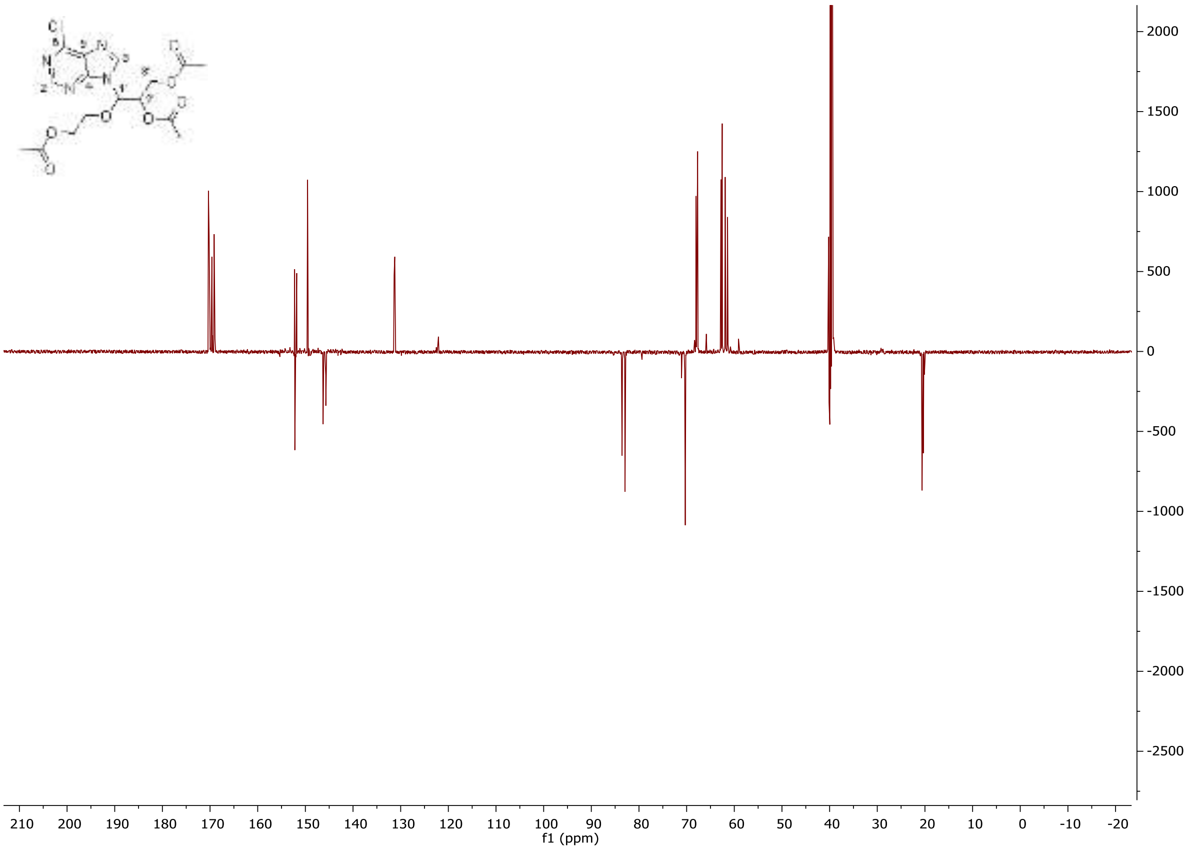

3-((6-Chloro-9*H*-purin-9-yl)methoxy)propyl acetate (**7**)

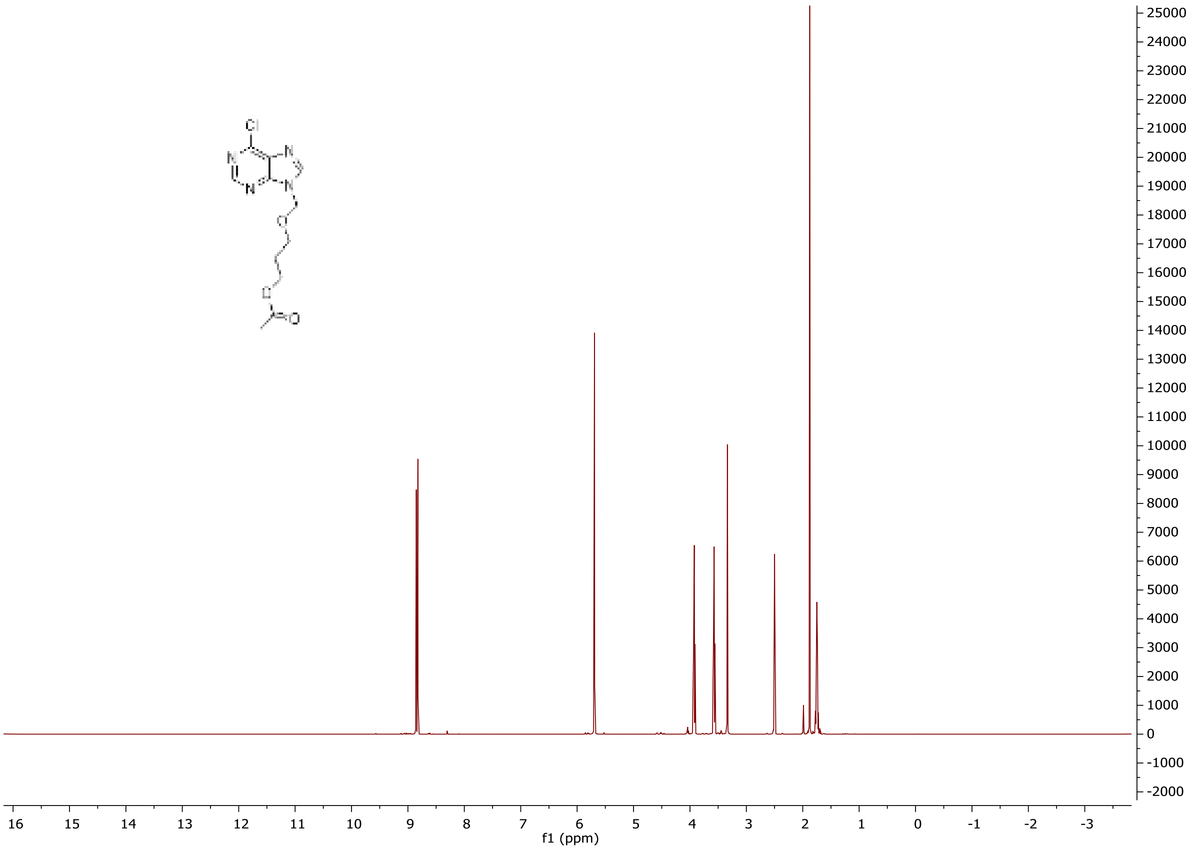

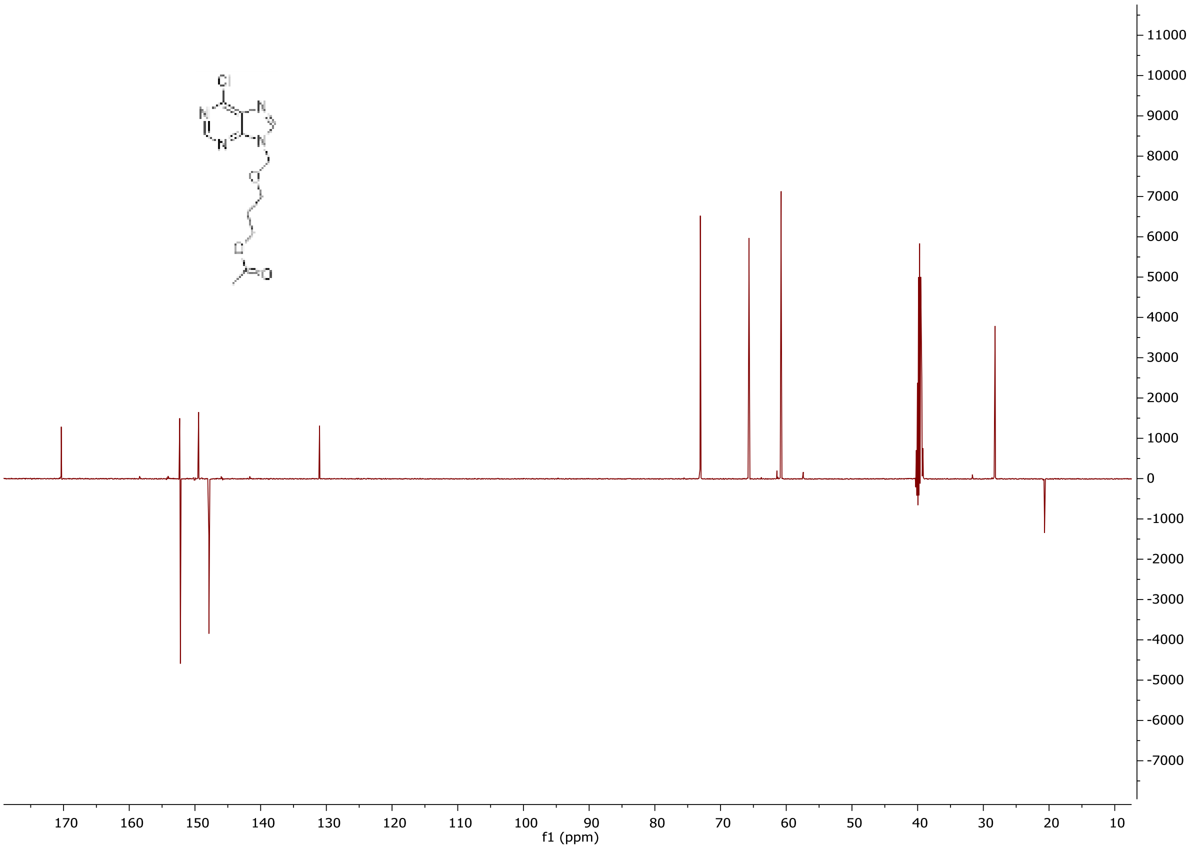

S-(2-((6-Chloro-9*H*-purin-9-yl)methoxy)ethyl) ethanethioate (**8**)

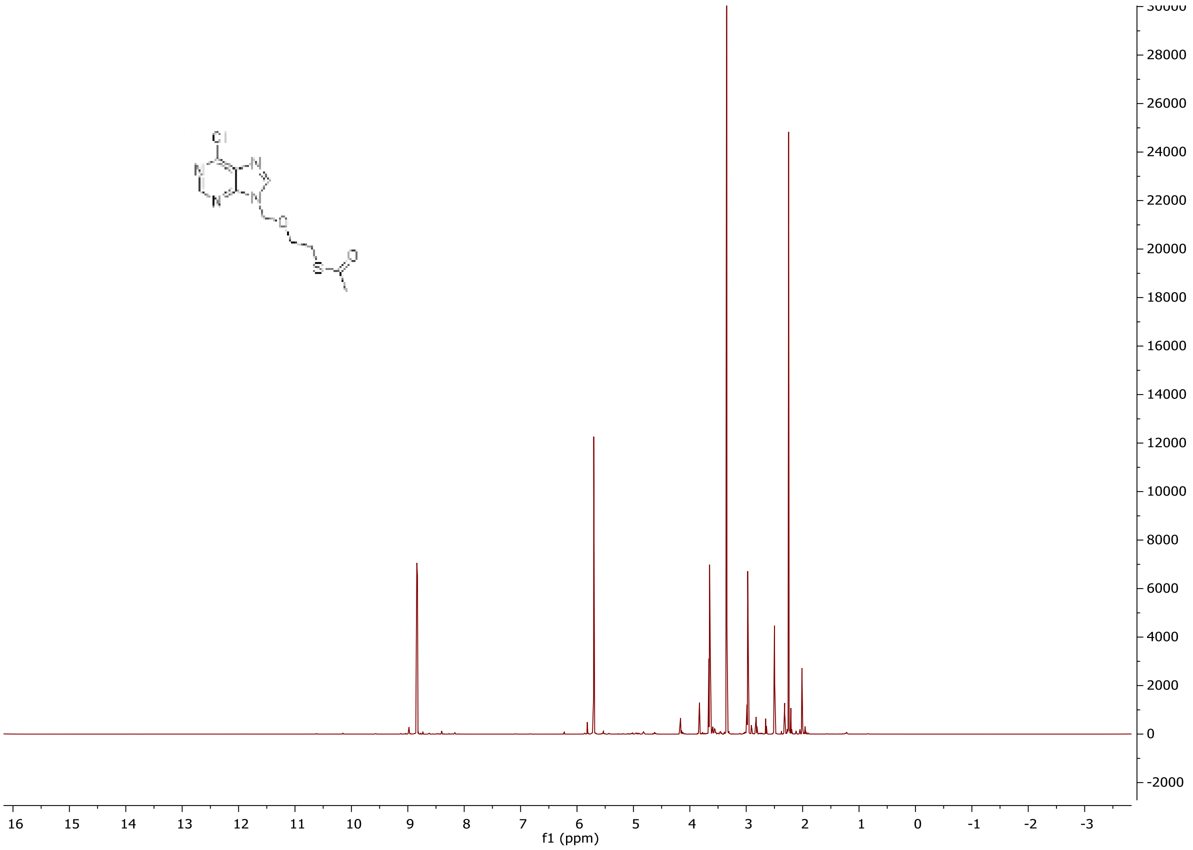

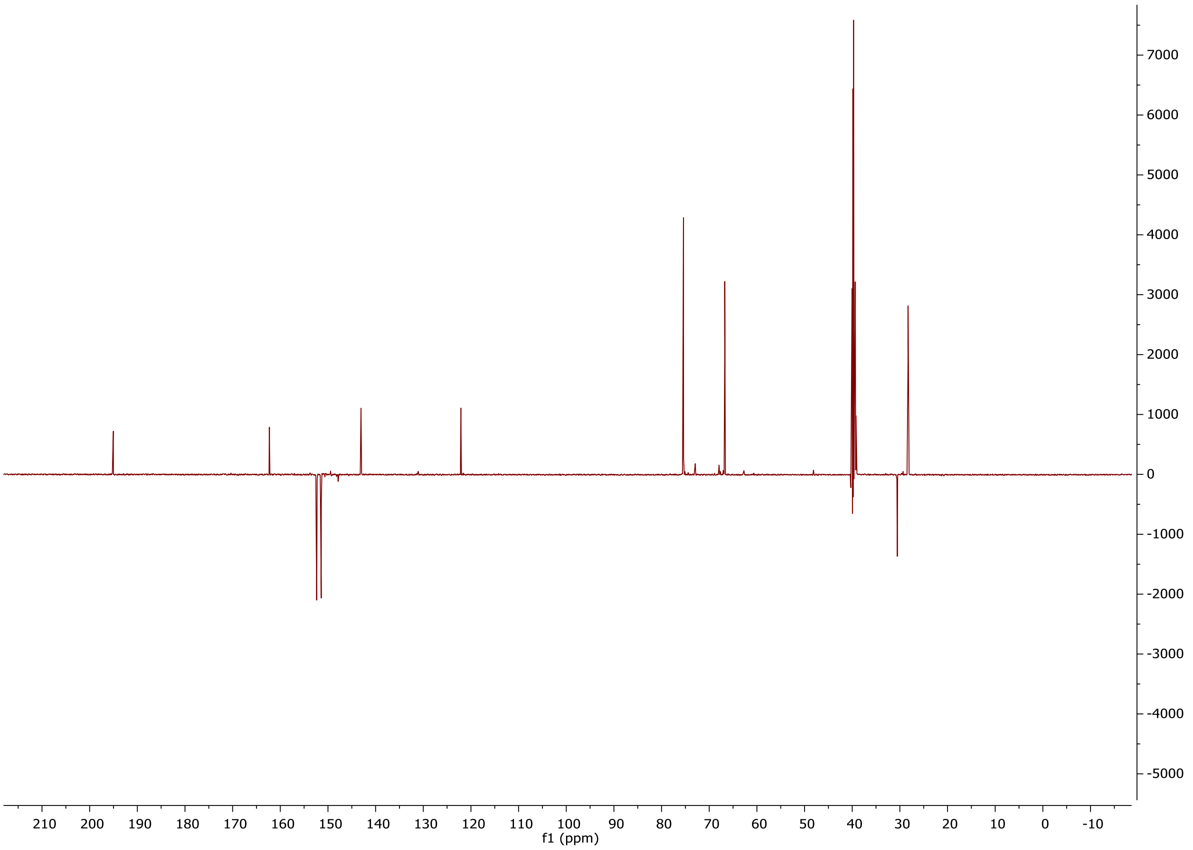

S-(2-((6-Chloro-7*H*-purin-7-yl)methoxy)ethyl) ethanethioate (**9**)

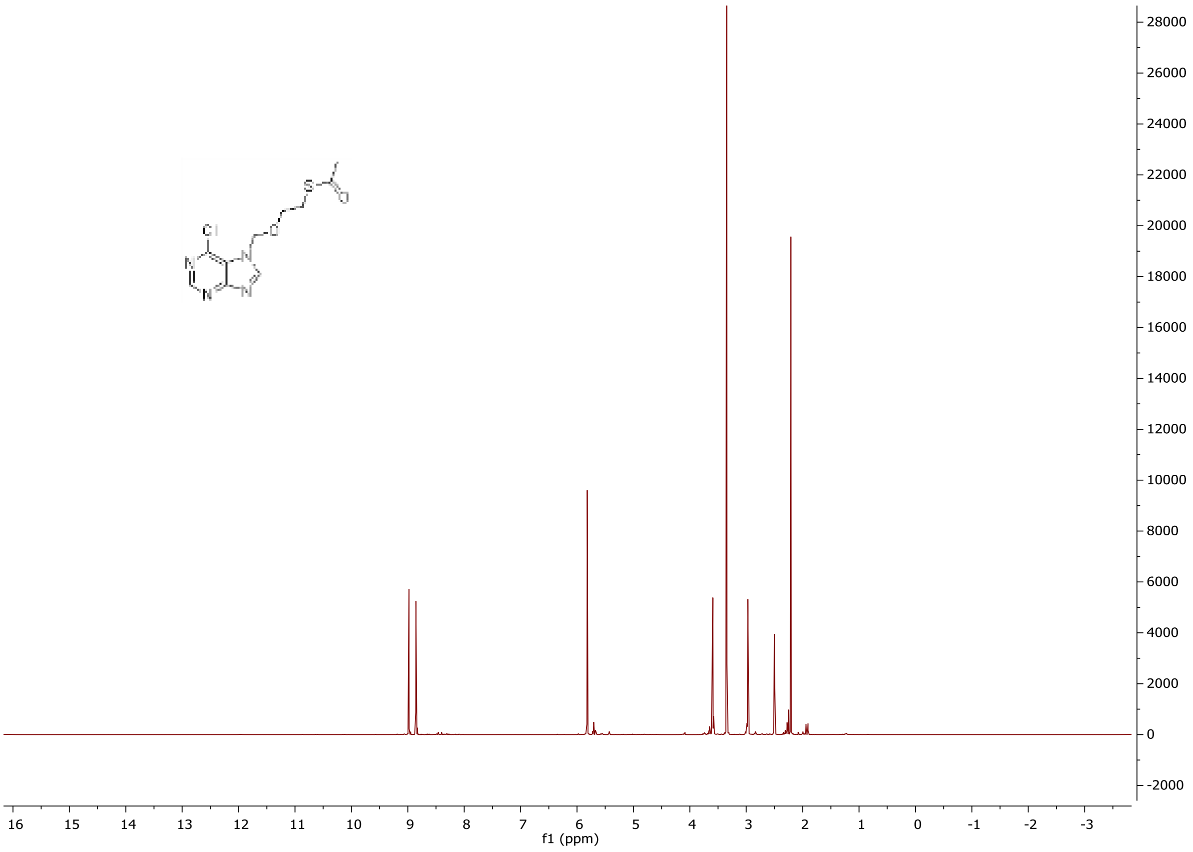

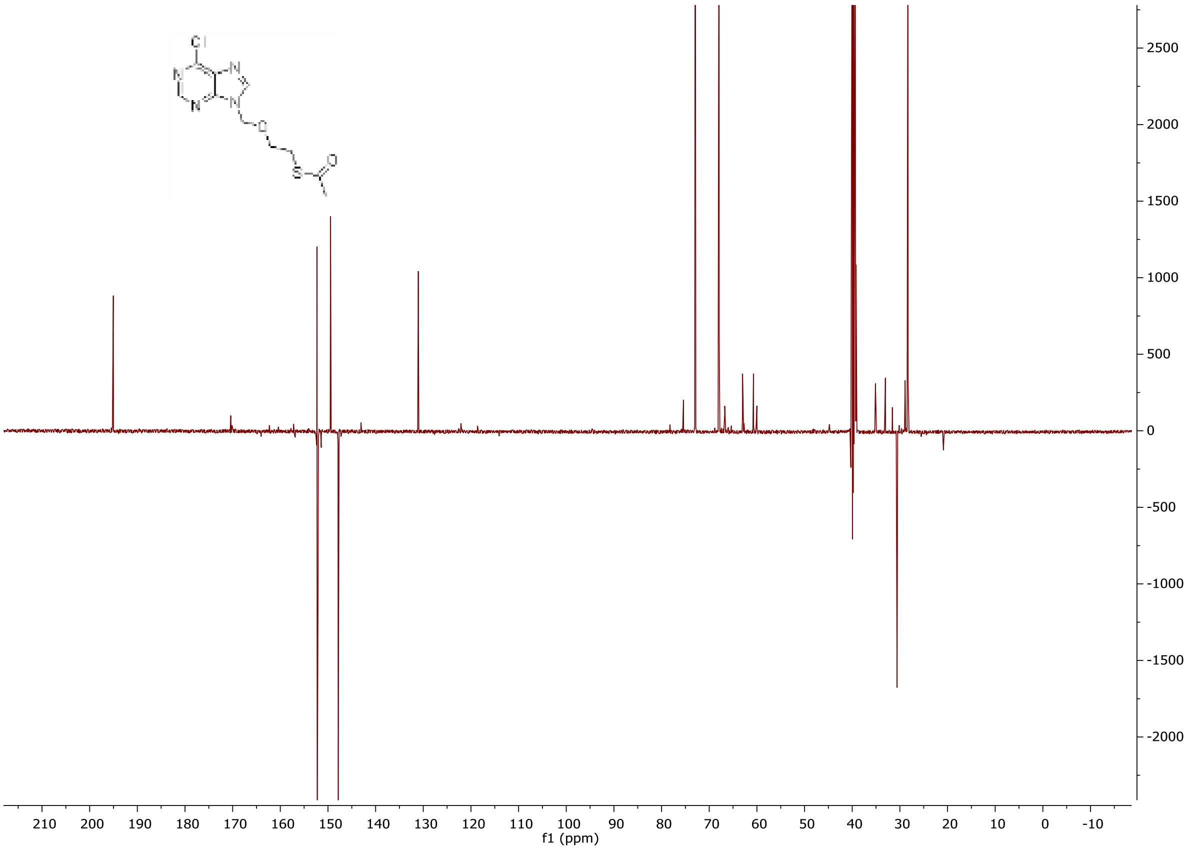

2-(2-Bromo-1-(6-chloro-2-fluoro-9*H*-purin-9-yl)ethoxy)ethyl acetate (**12**)

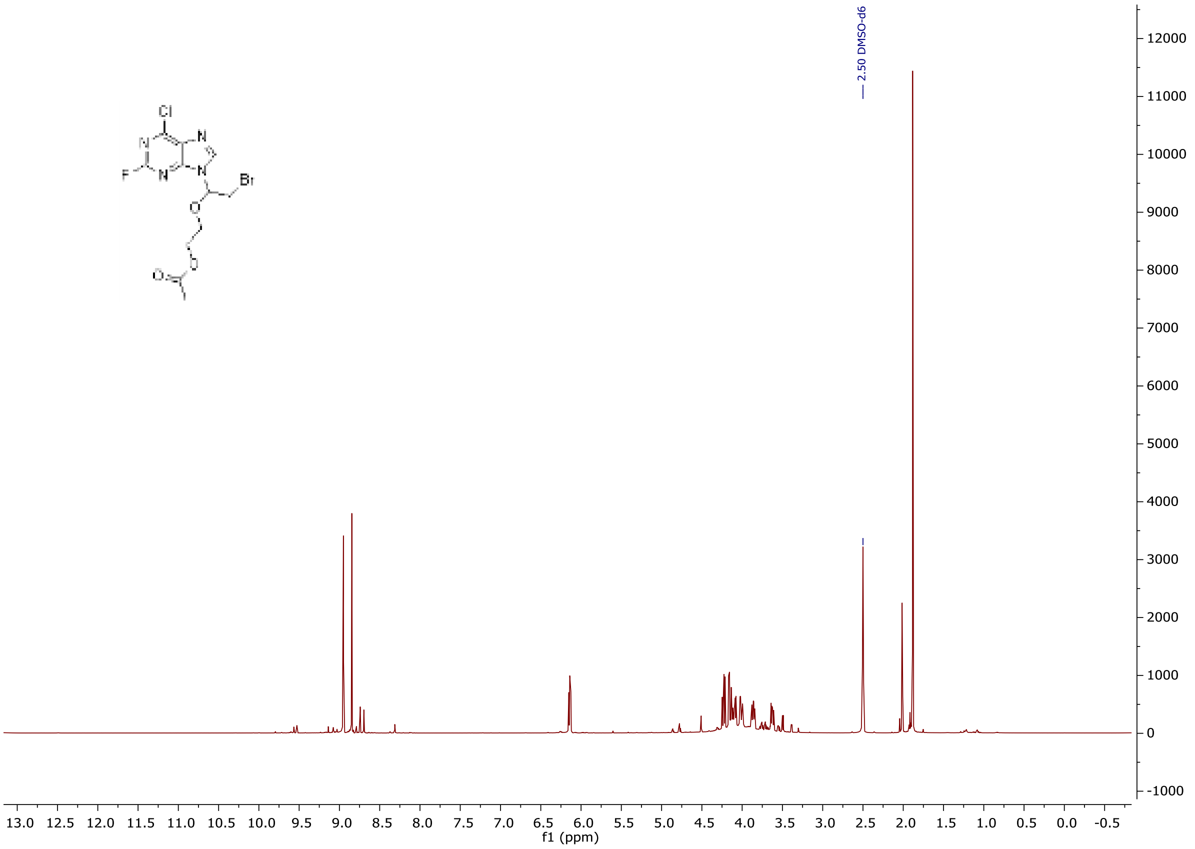

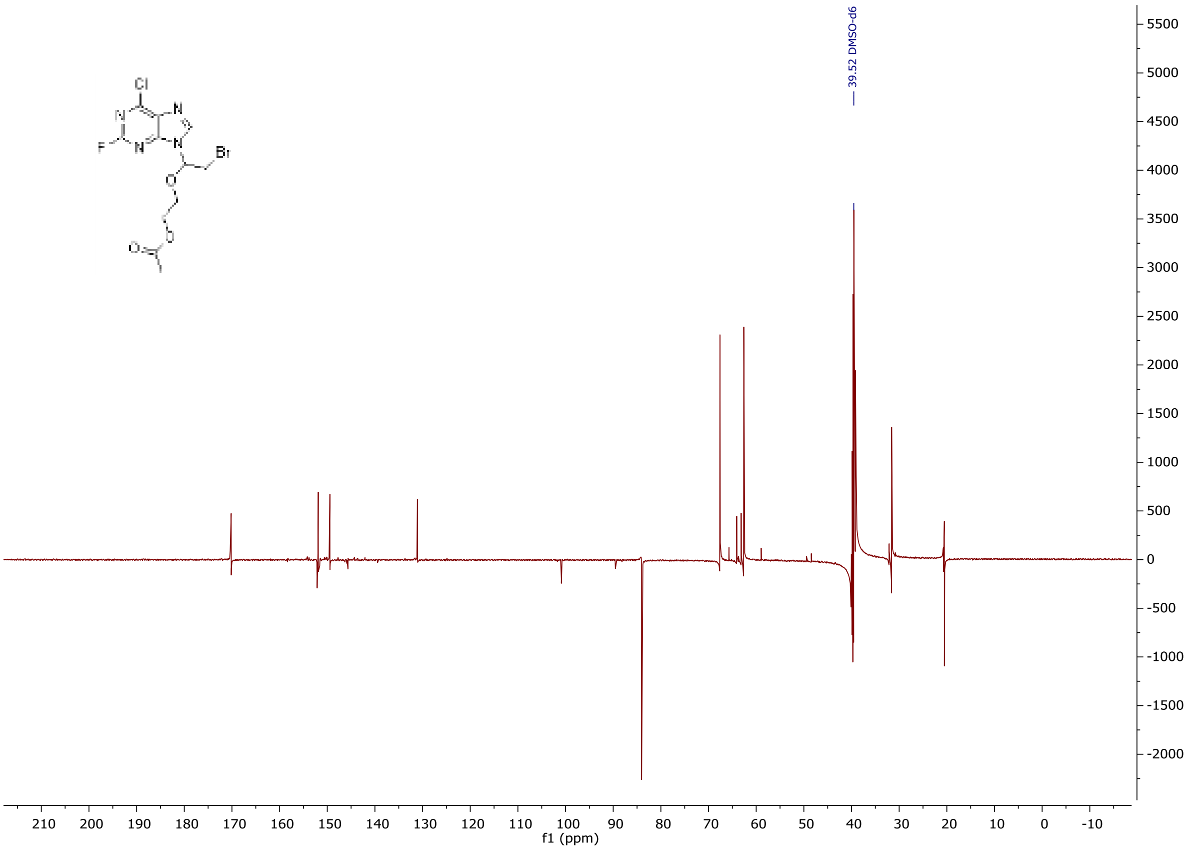

2-(2-Bromo-1-(2,6-dichloro-9*H*-purin-9-yl)ethoxy)ethyl acetate (**13**)

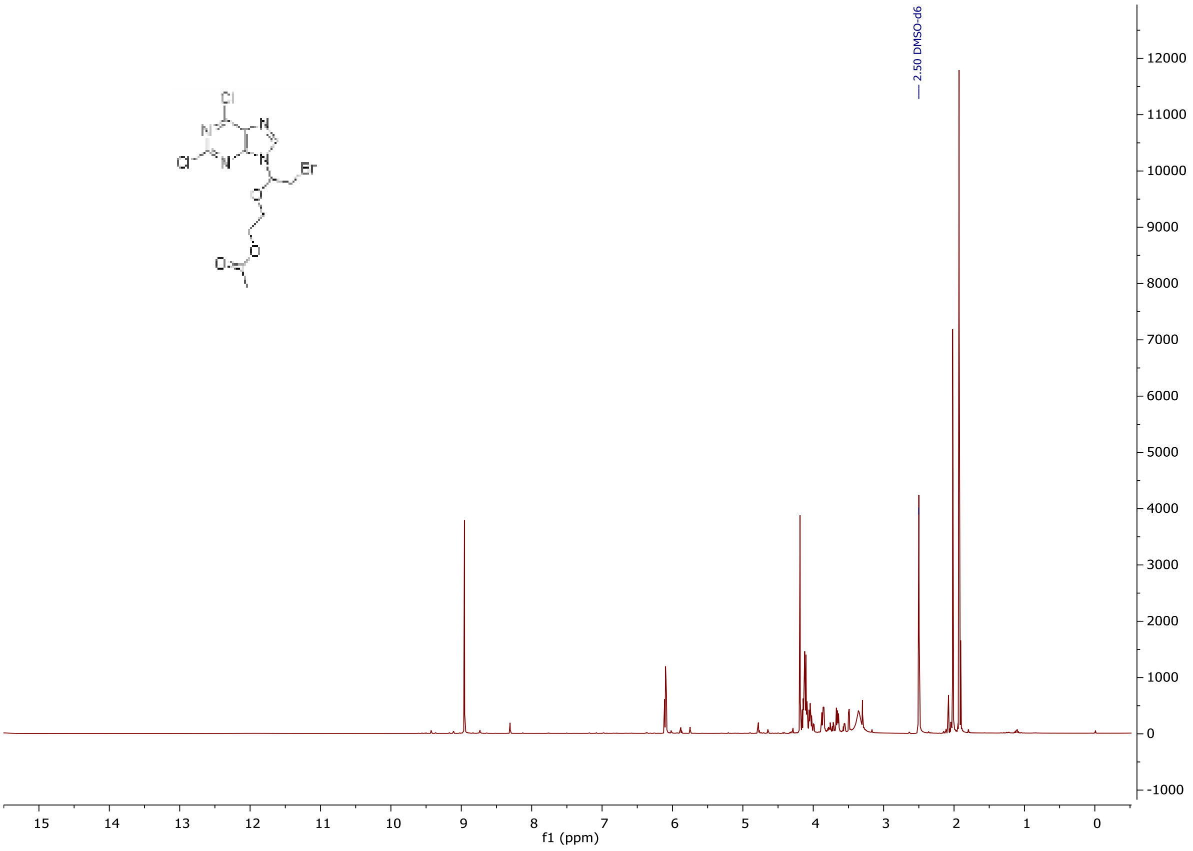

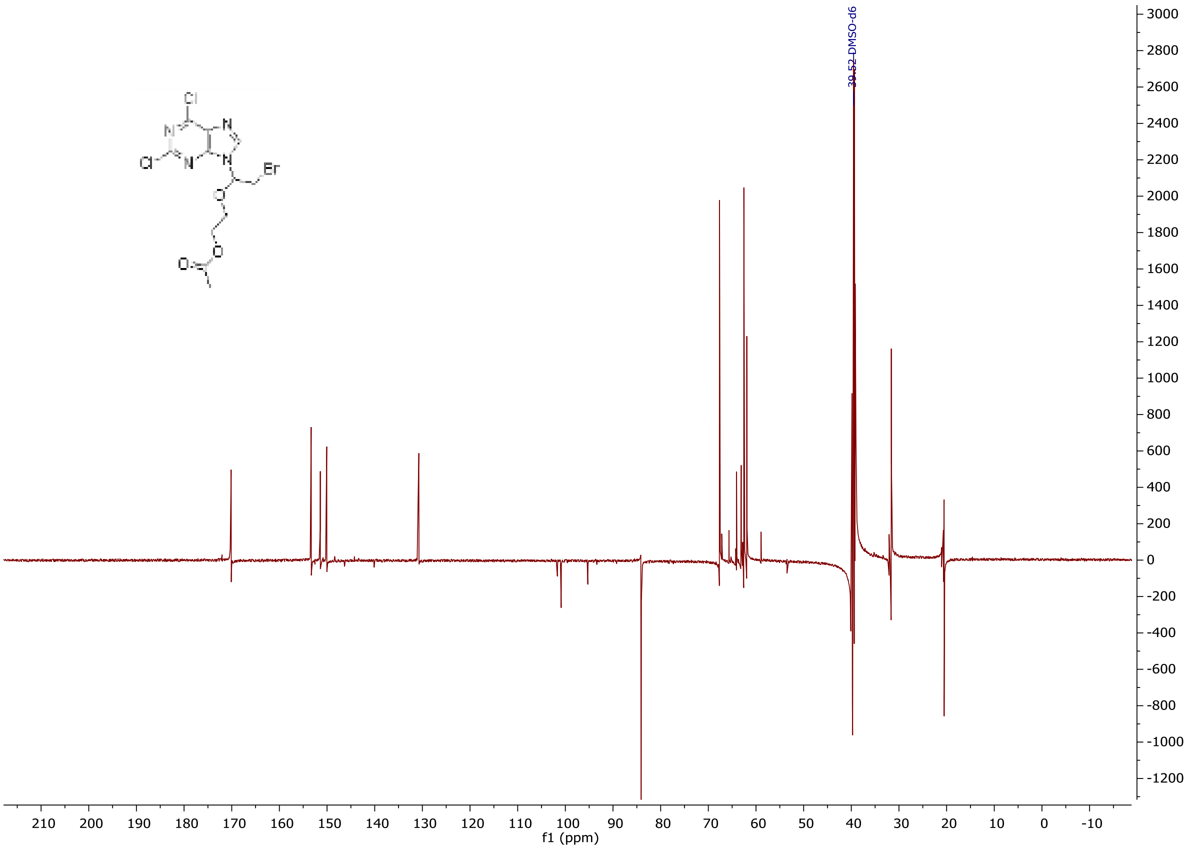

3-((2-Acetamido-6-chloro-9*H*-purin-9-yl)methoxy)-2-(tosyloxy)propyl acetate (**17**)

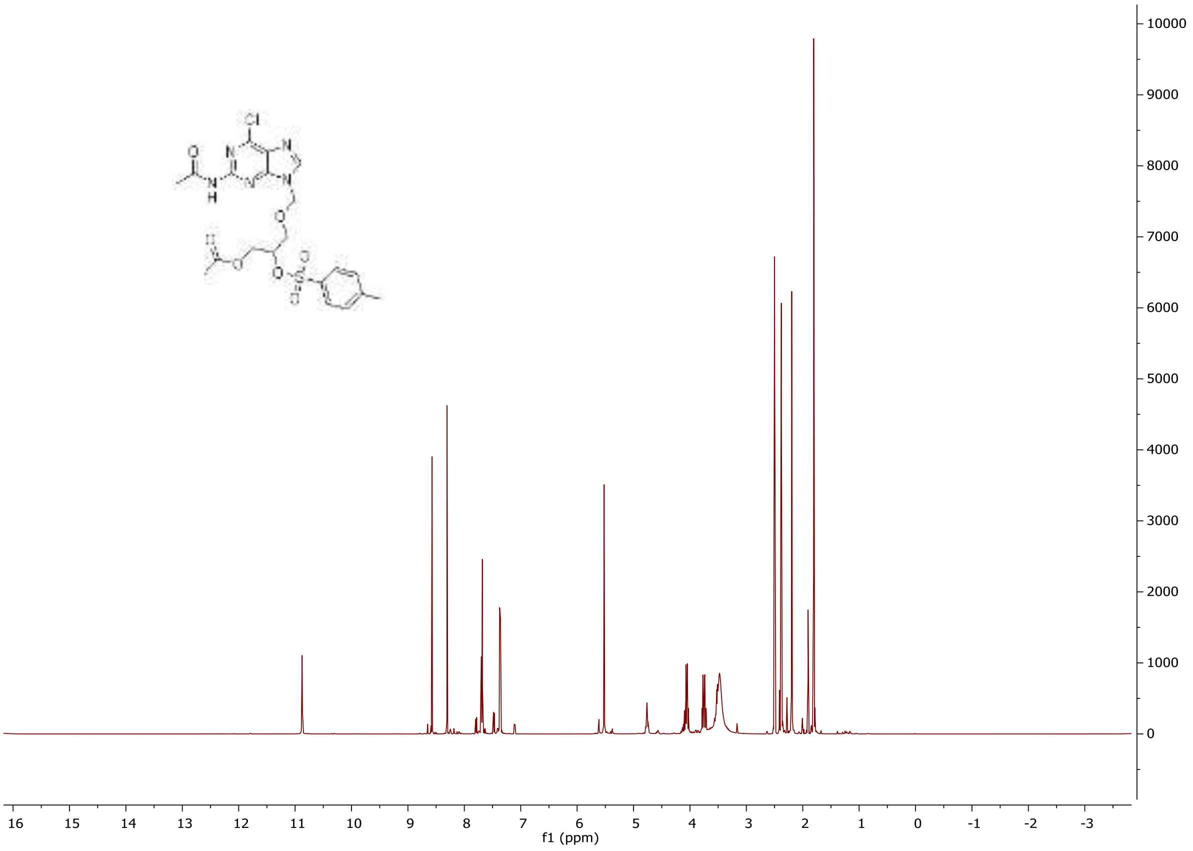

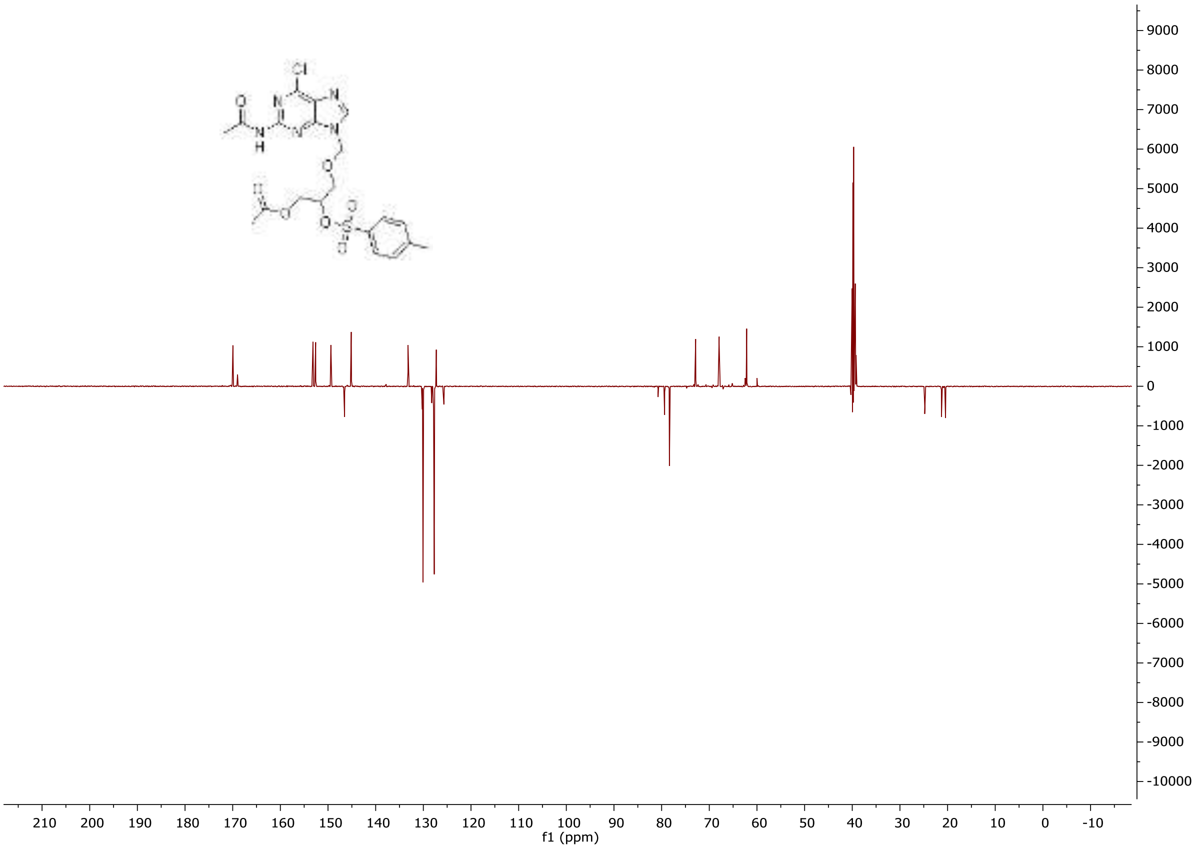

Benzyl 3-((2-acetamido-6-chloro-9*H*-purin-9-yl)methoxy)-4-acetoxypyrrolidine-1-carboxylate (**20**)

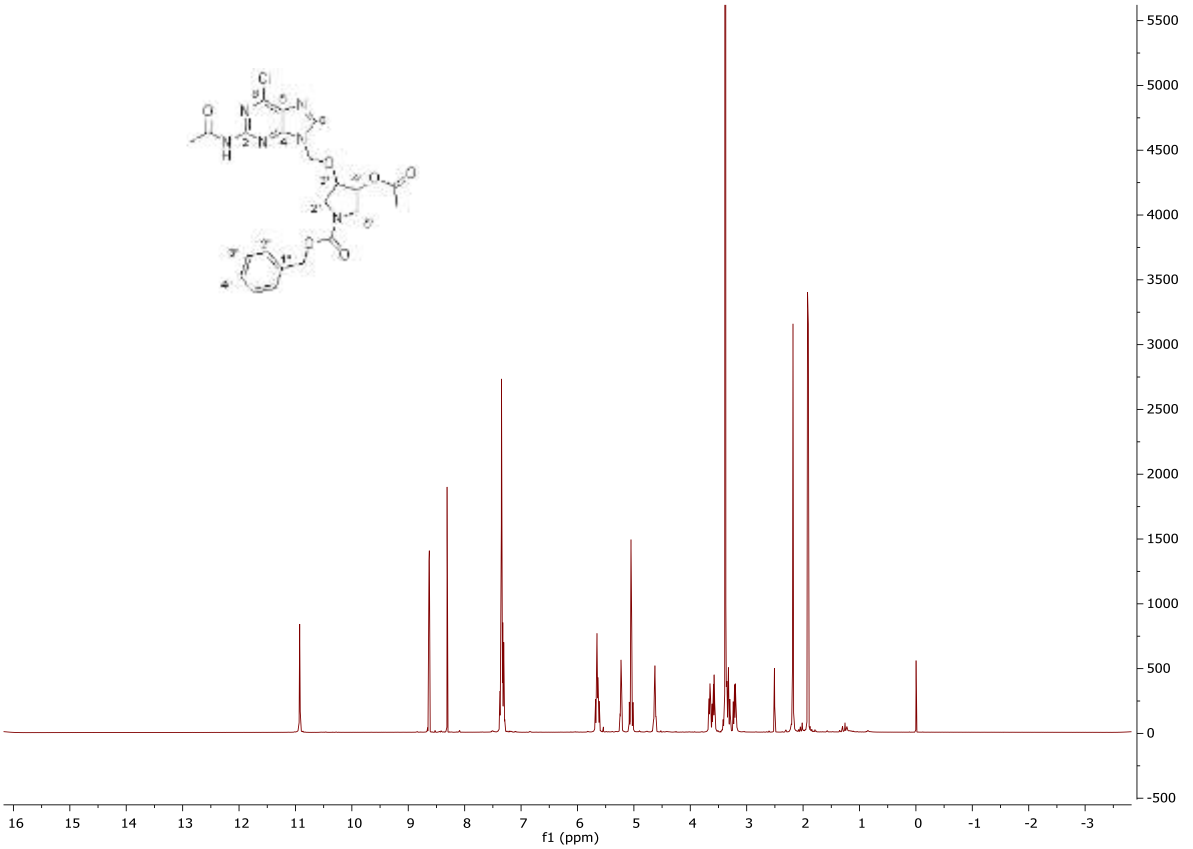

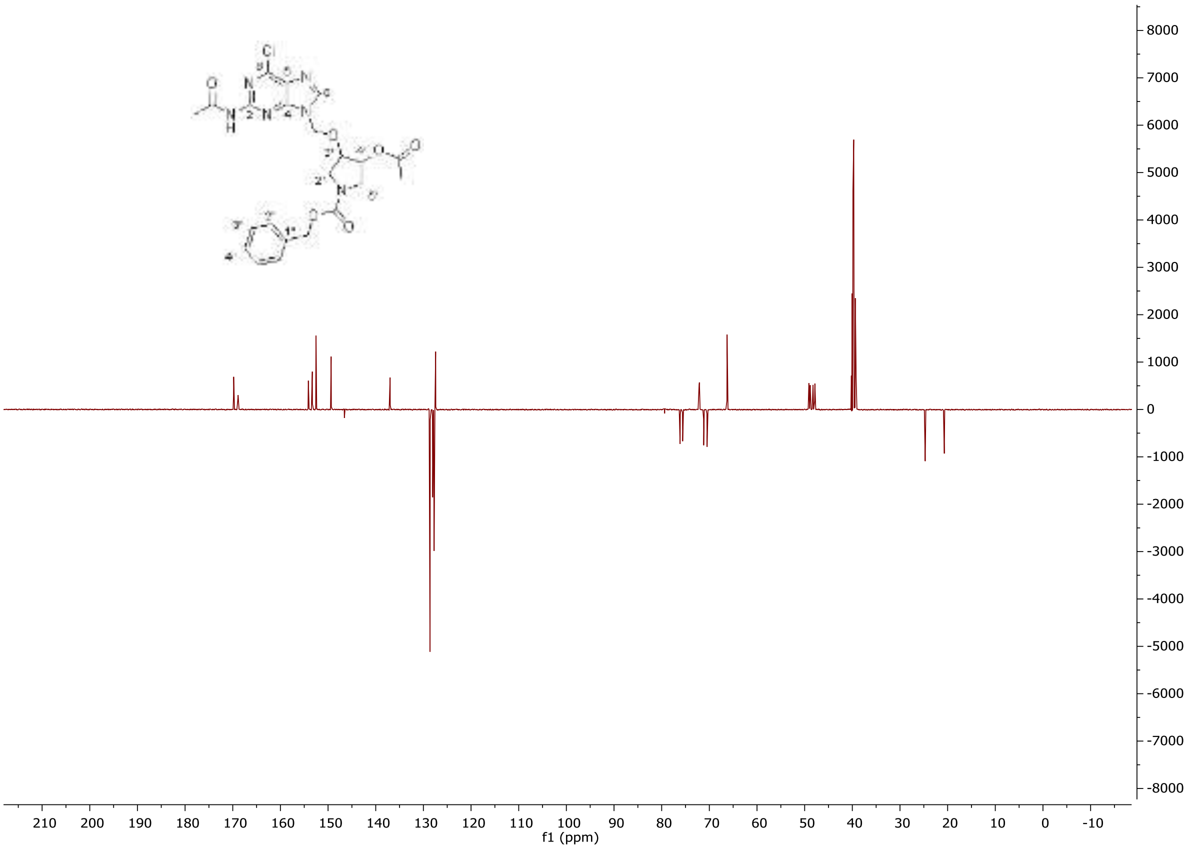

2-(1-(6-Amino-4-chloro-2*H*-pyrazolo[3,4-*d*]pyrimidin-2-yl)-2-bromoethoxy)ethyl acetate (**22**)

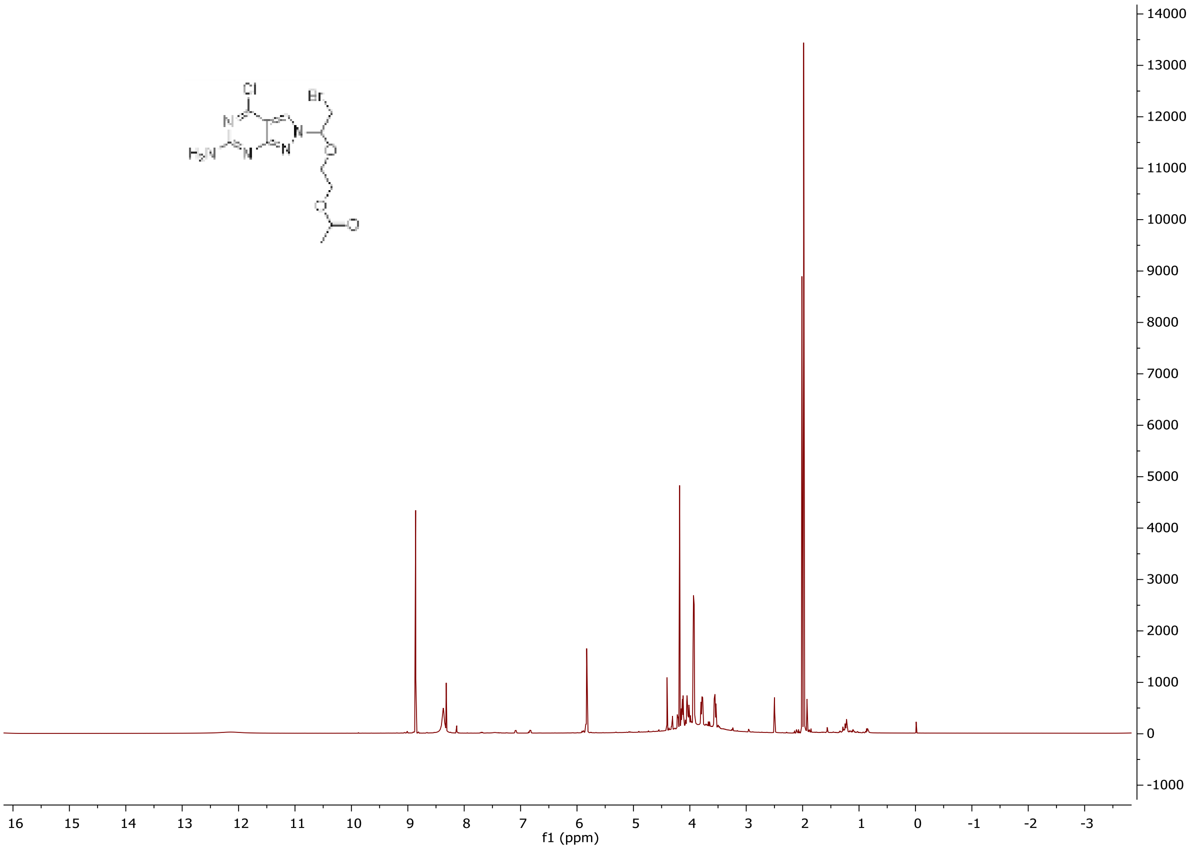

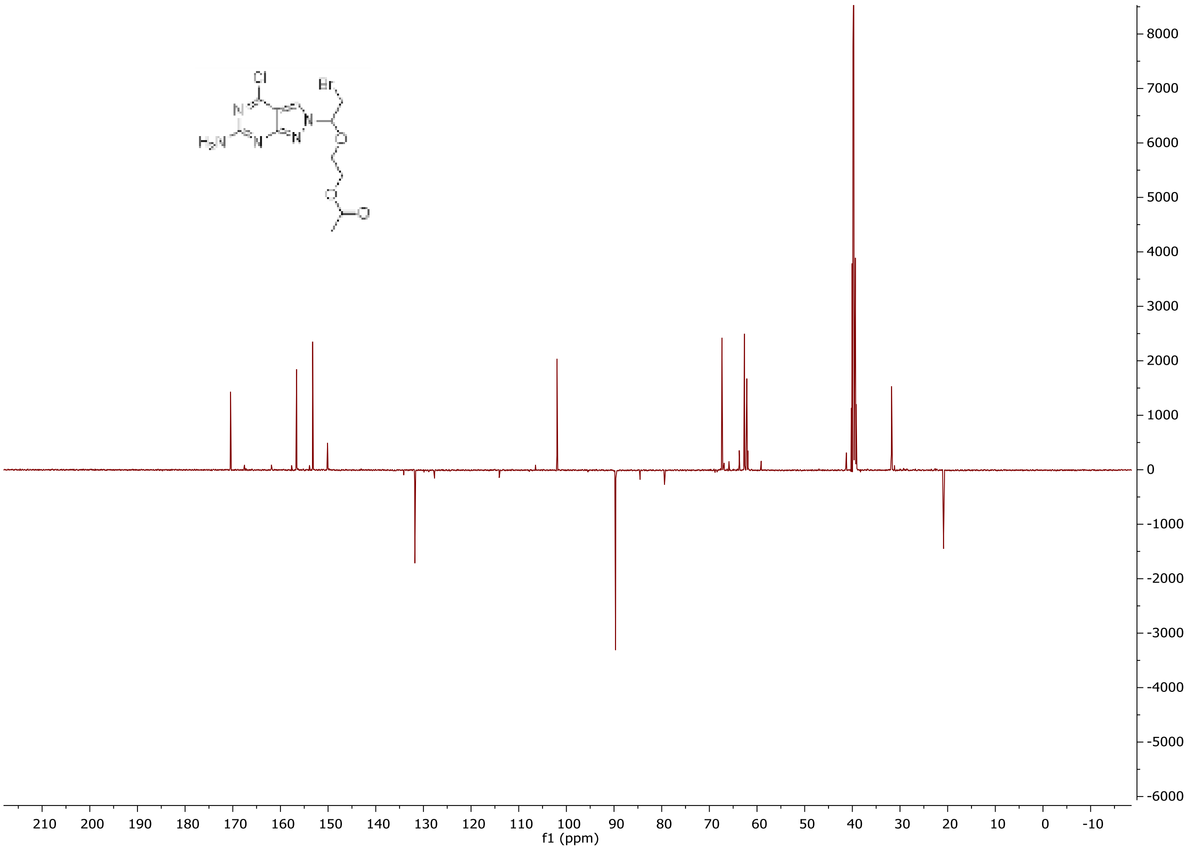

2-(1-(Guanine-7-yl)-2-bromoethoxy)ethyl acetate (**23**)

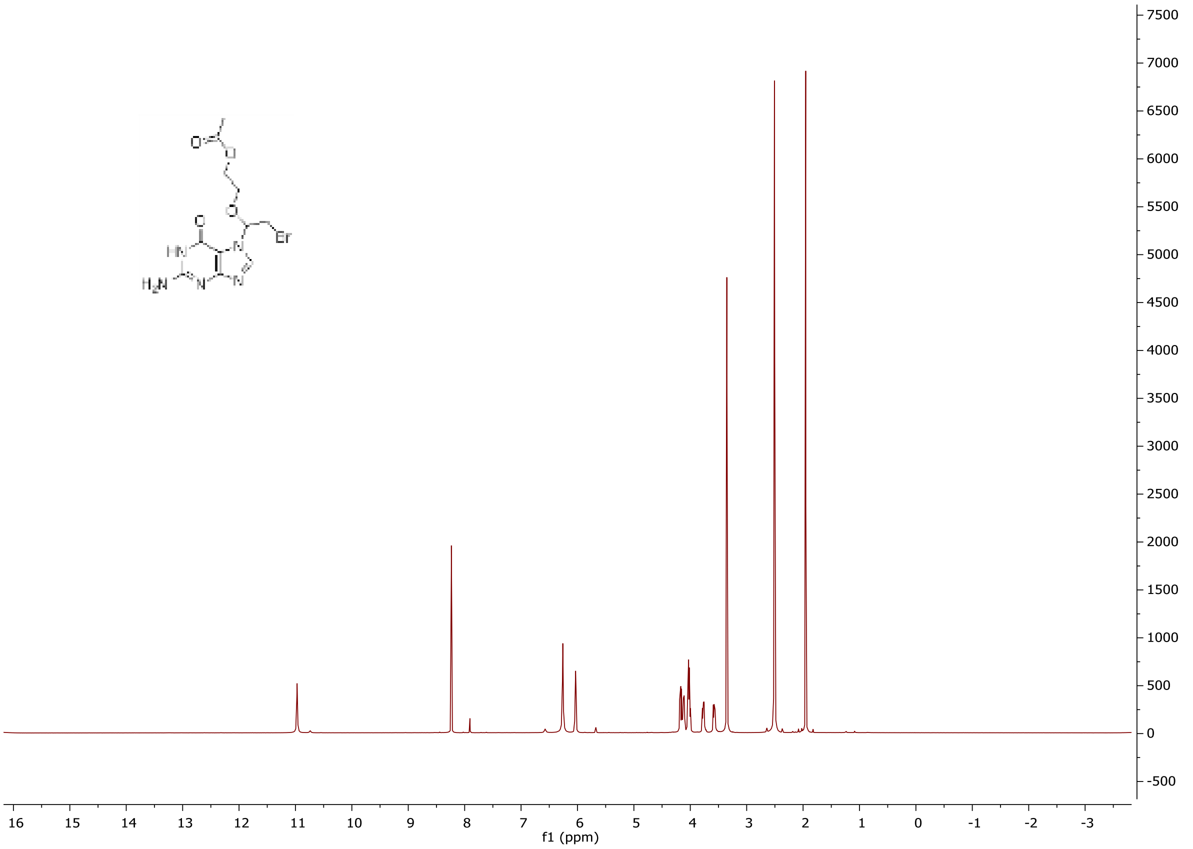

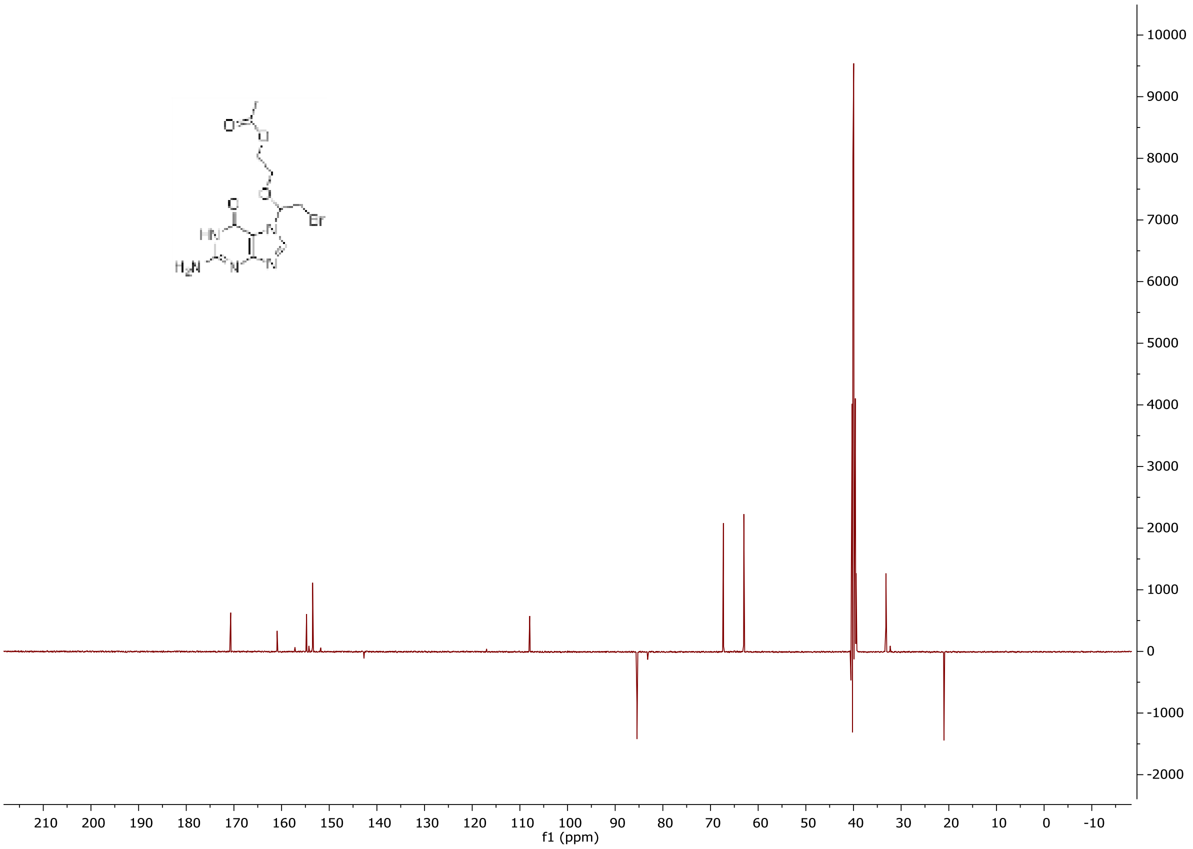

2-(1-(Adenin-9-yl)-2-bromoethoxy)ethyl acetate (**24**)

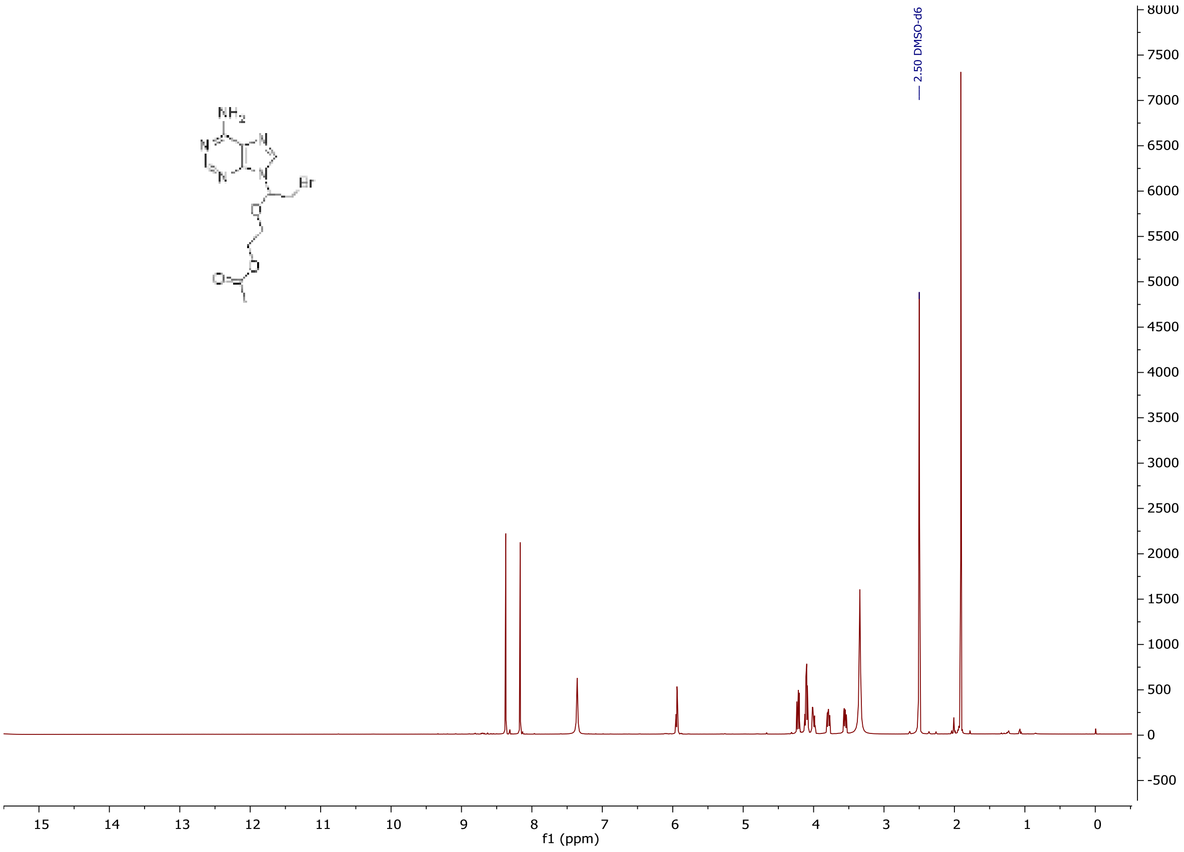

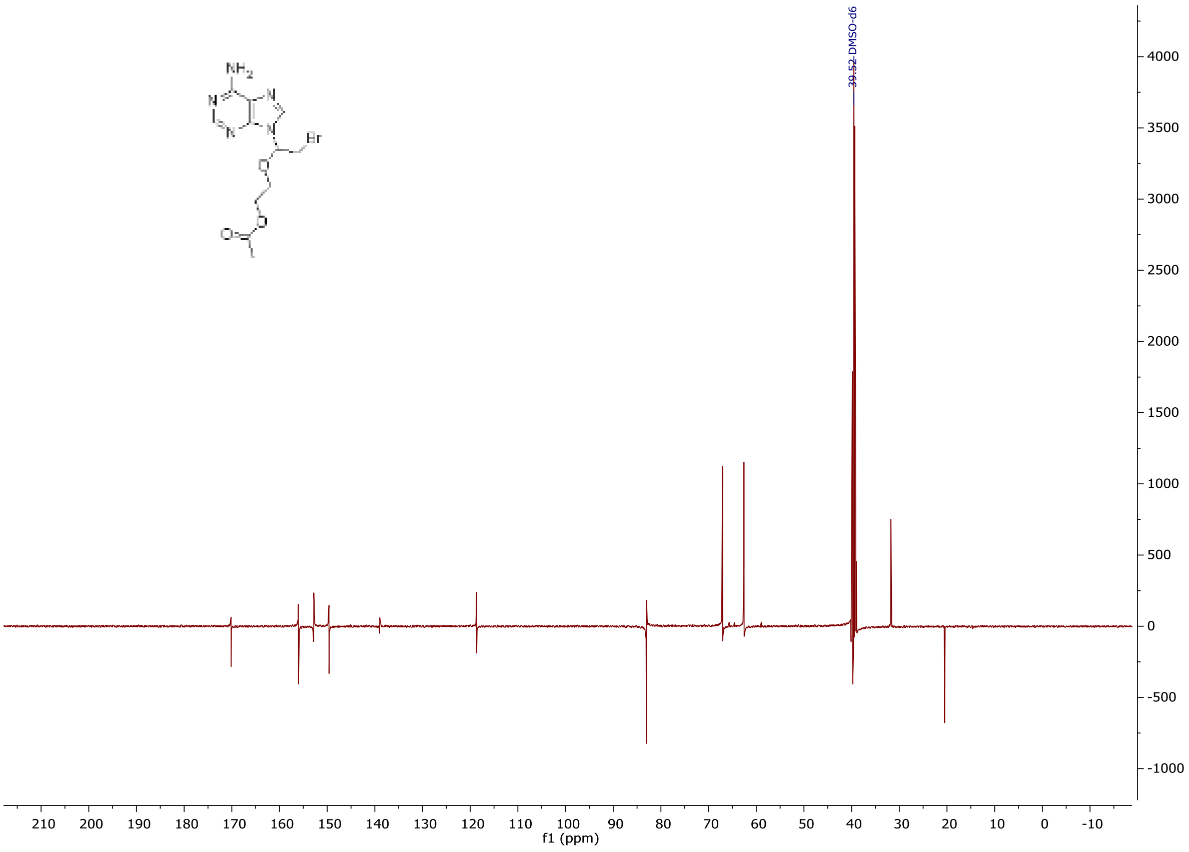

2-((6-Benzamido-9*H*-purin-9-yl)methoxy)ethyl acetate (**25**)

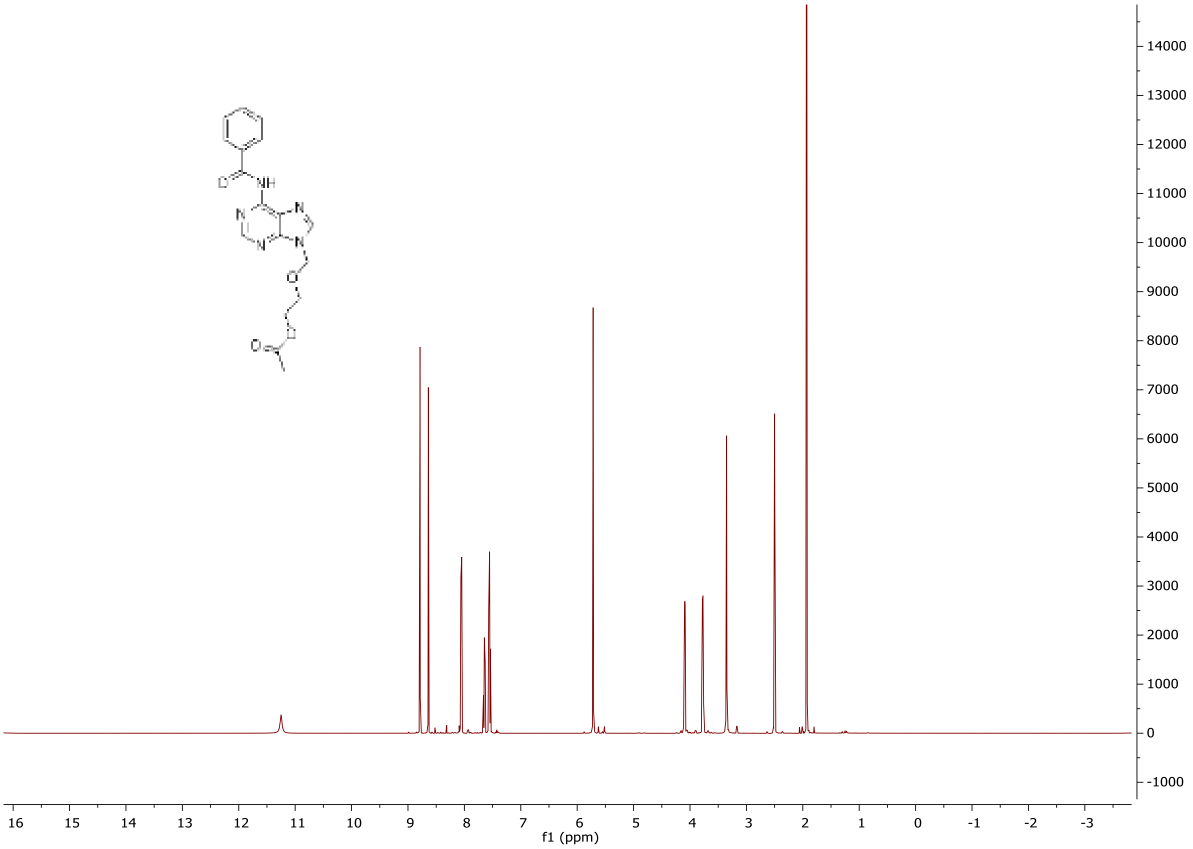

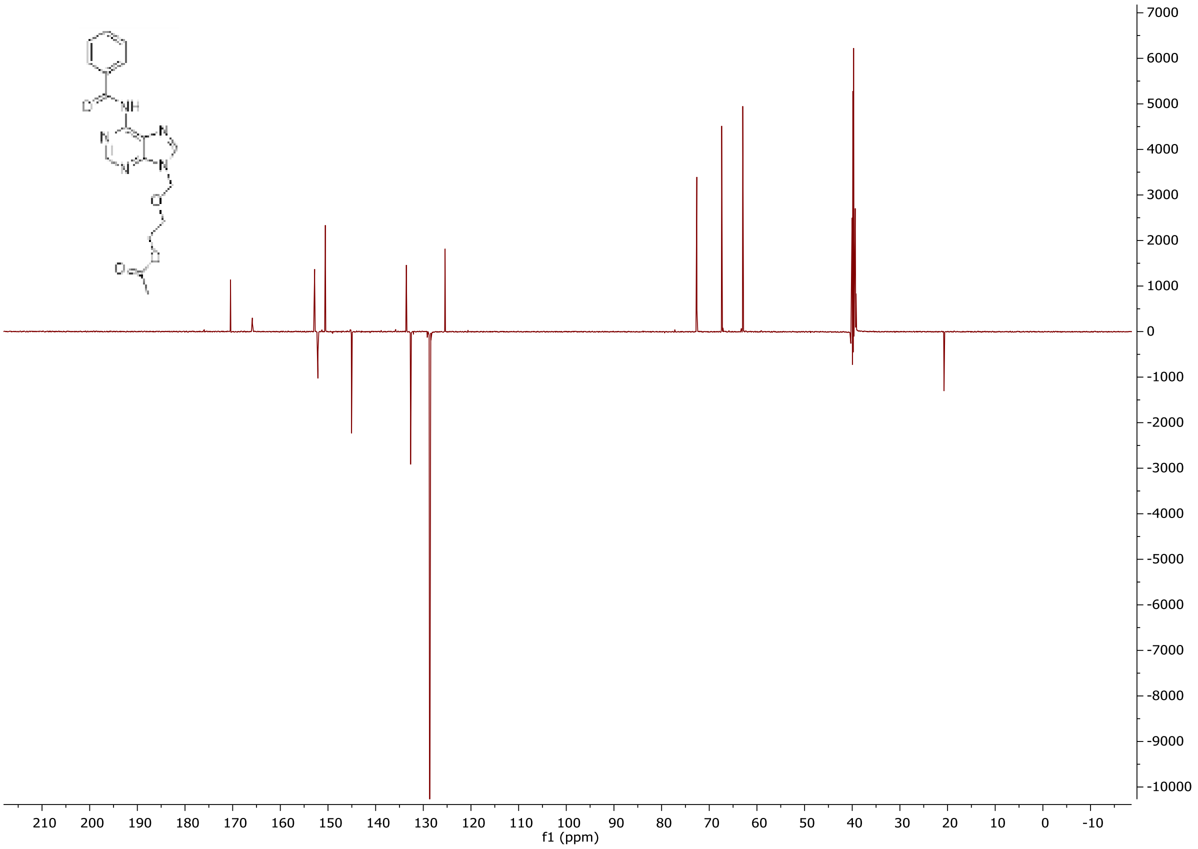

2-((6-Benzamido-9*H*-purin-9-yl)methoxy)but-3-en-1-yl acetate (**26**)

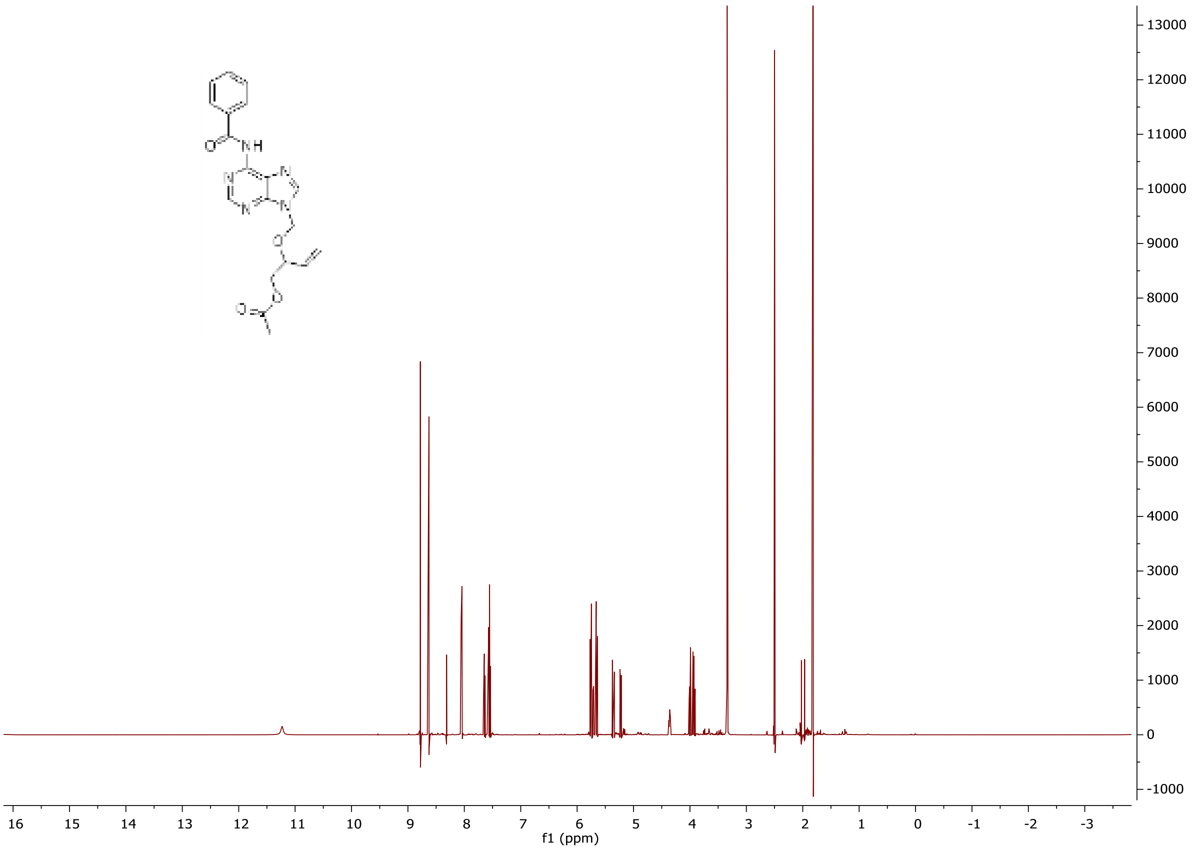

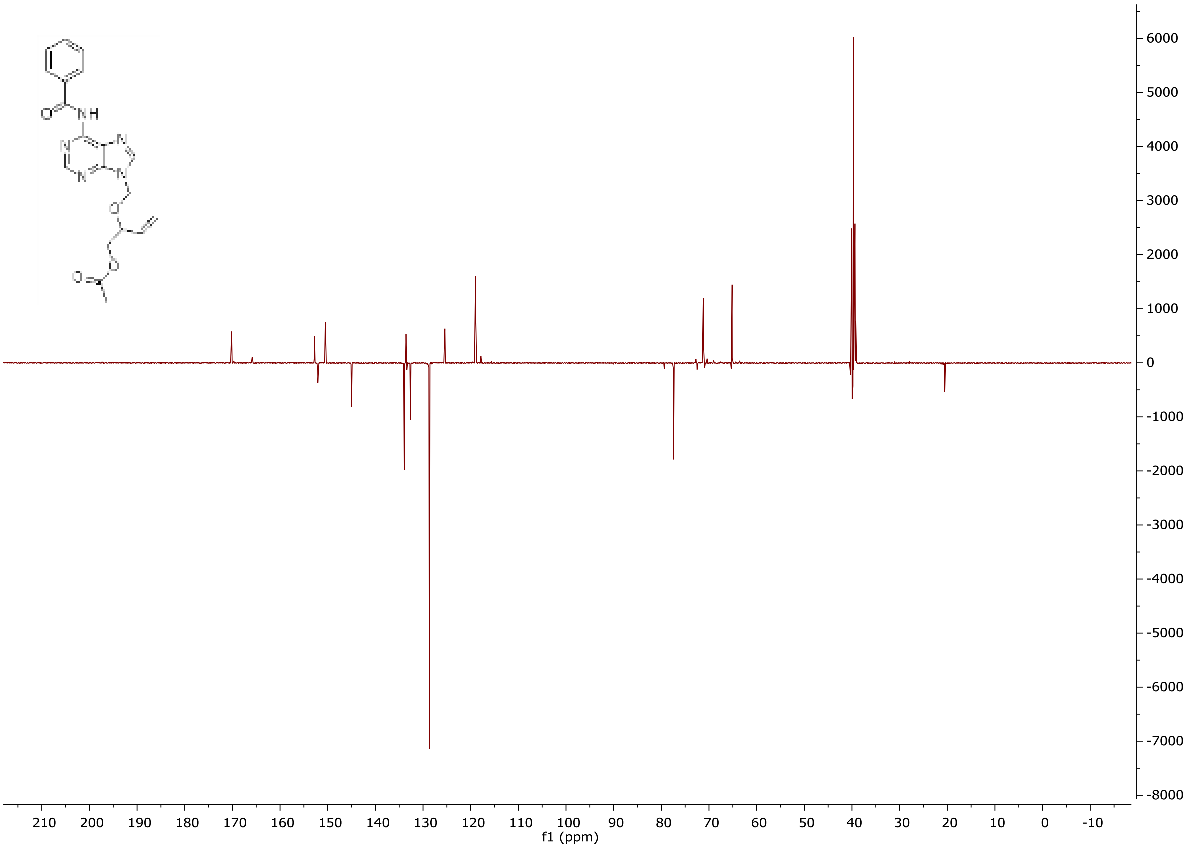

2-(2-Bromo-1-(2,6-diamino-9*H*-purin-9-yl)ethoxy)ethyl acetate (**29**)

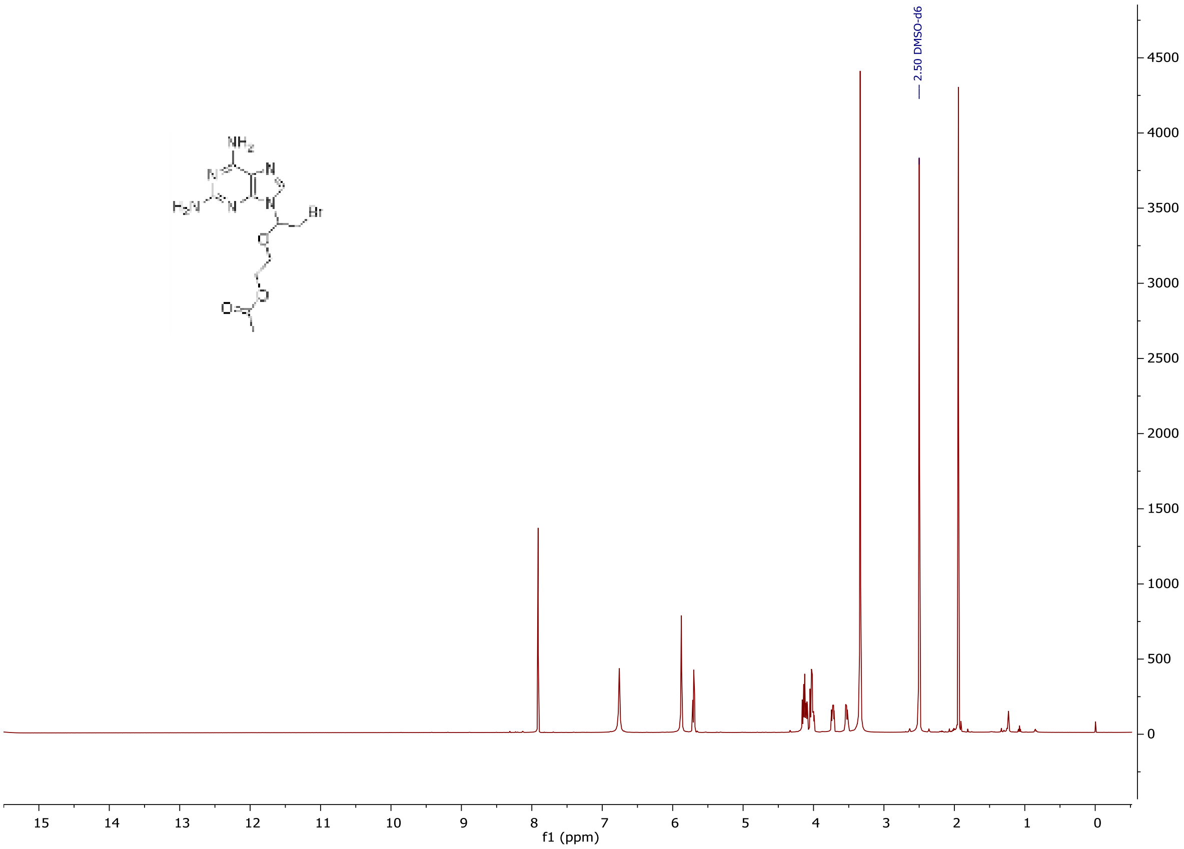

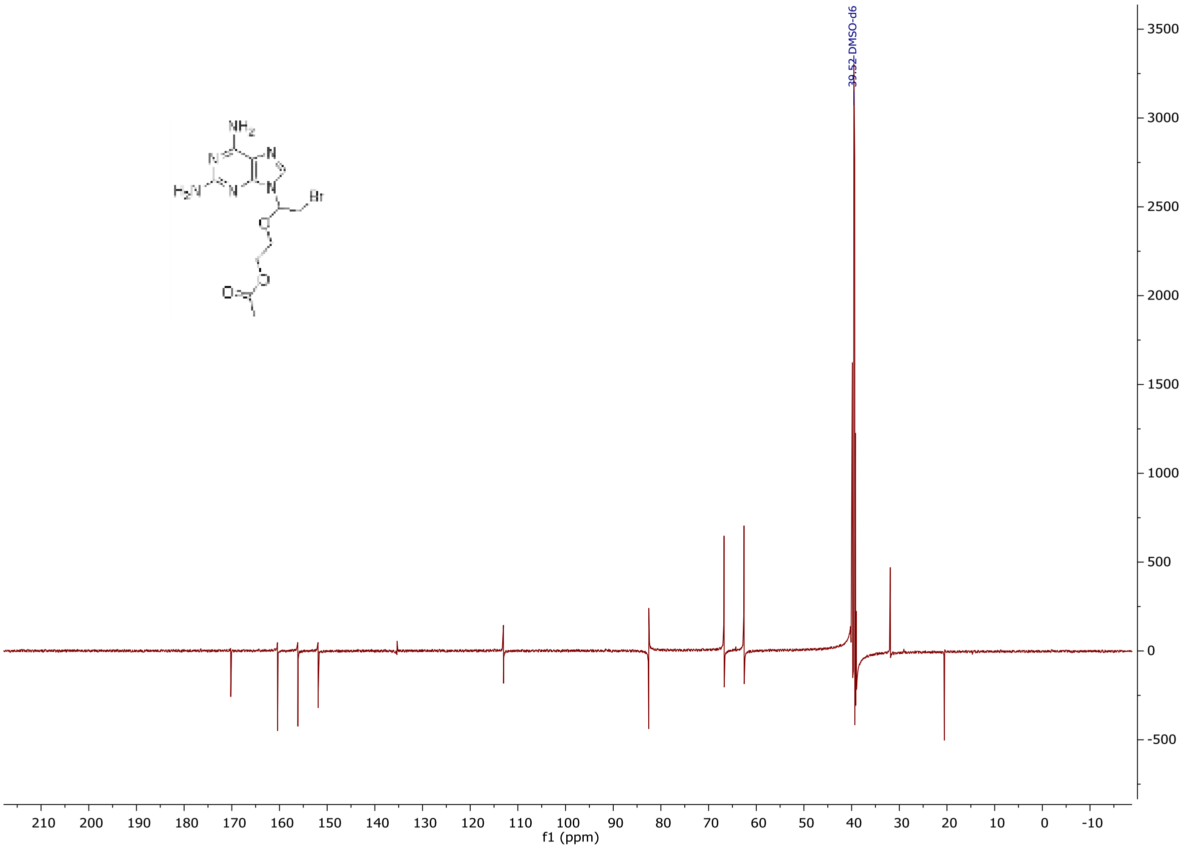

3-(2-Acetoxyethoxy)-3-(2,6-diacetamido-9*H*-purin-9-yl)propane-1,2-diyl diacetate (**30**)

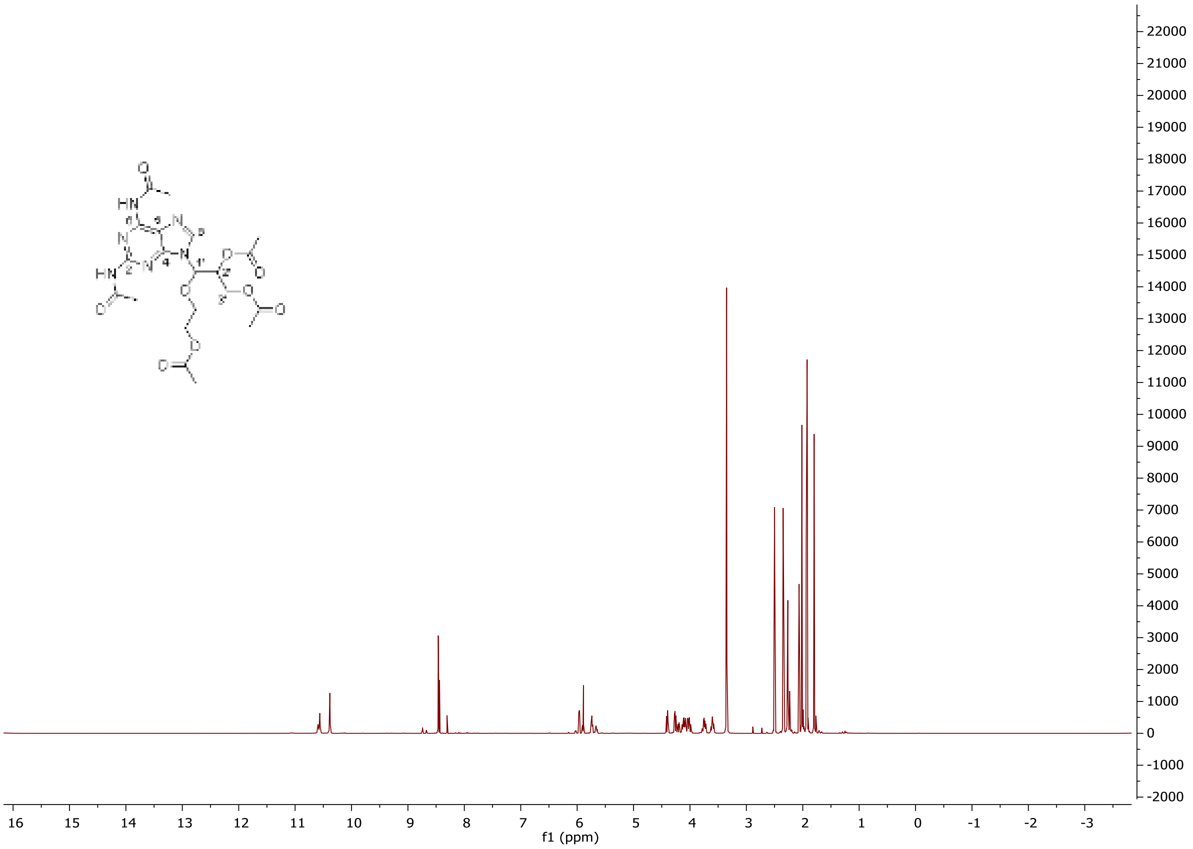

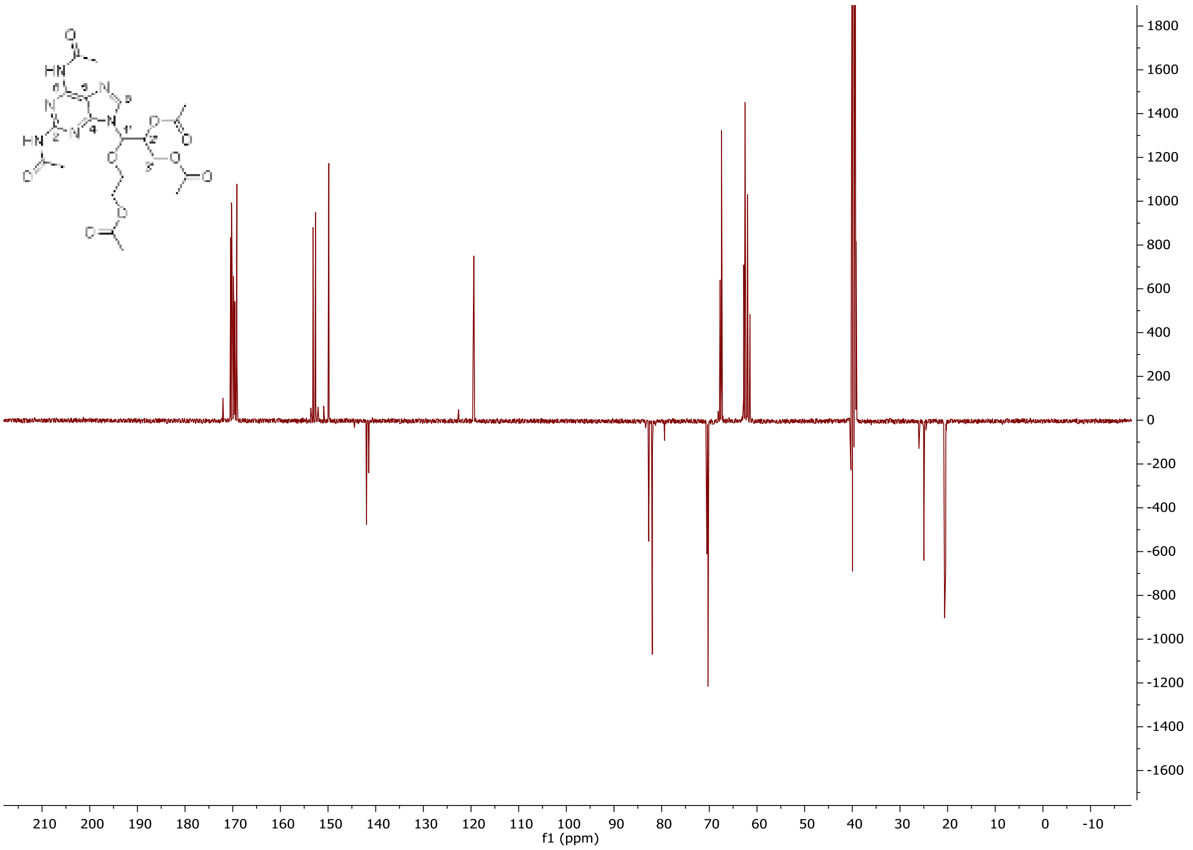

6-Chloro-9-(2-chloro-1-ethoxyethyl)-9*H*-purine (**31**)

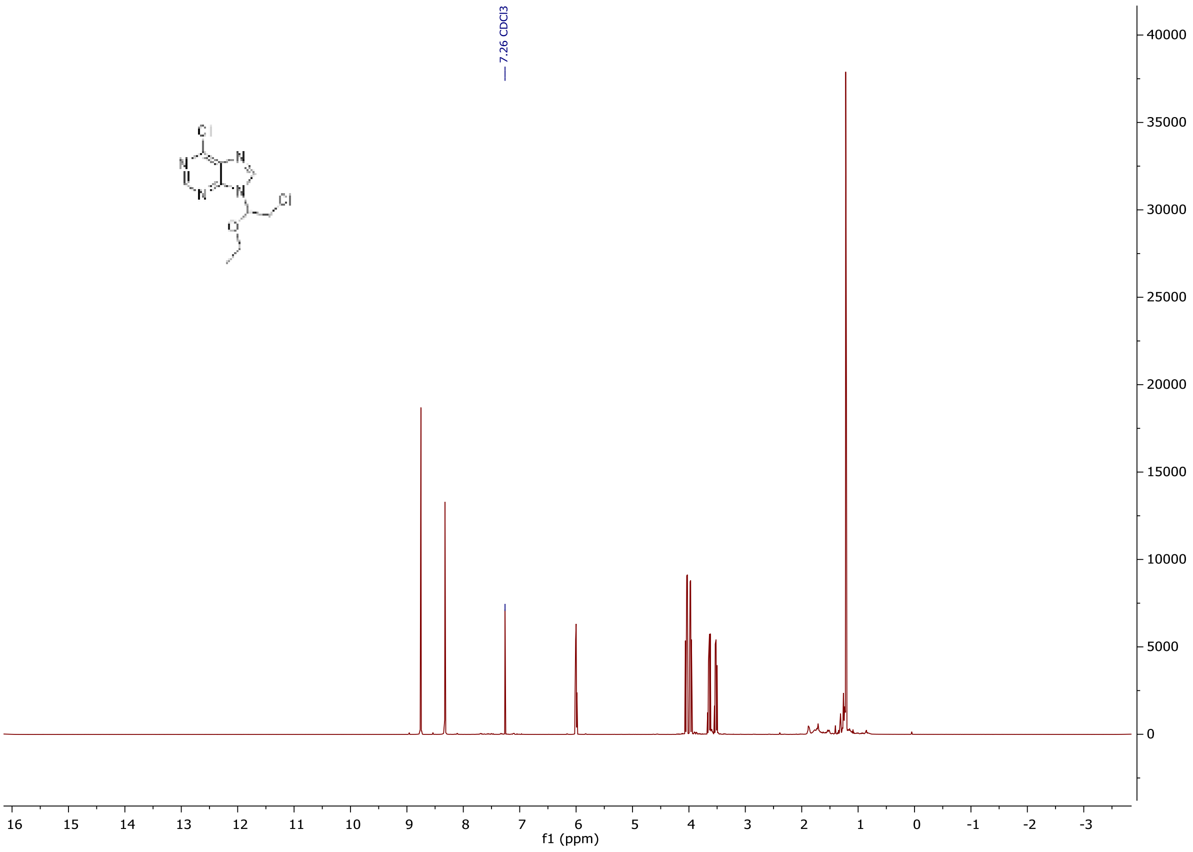

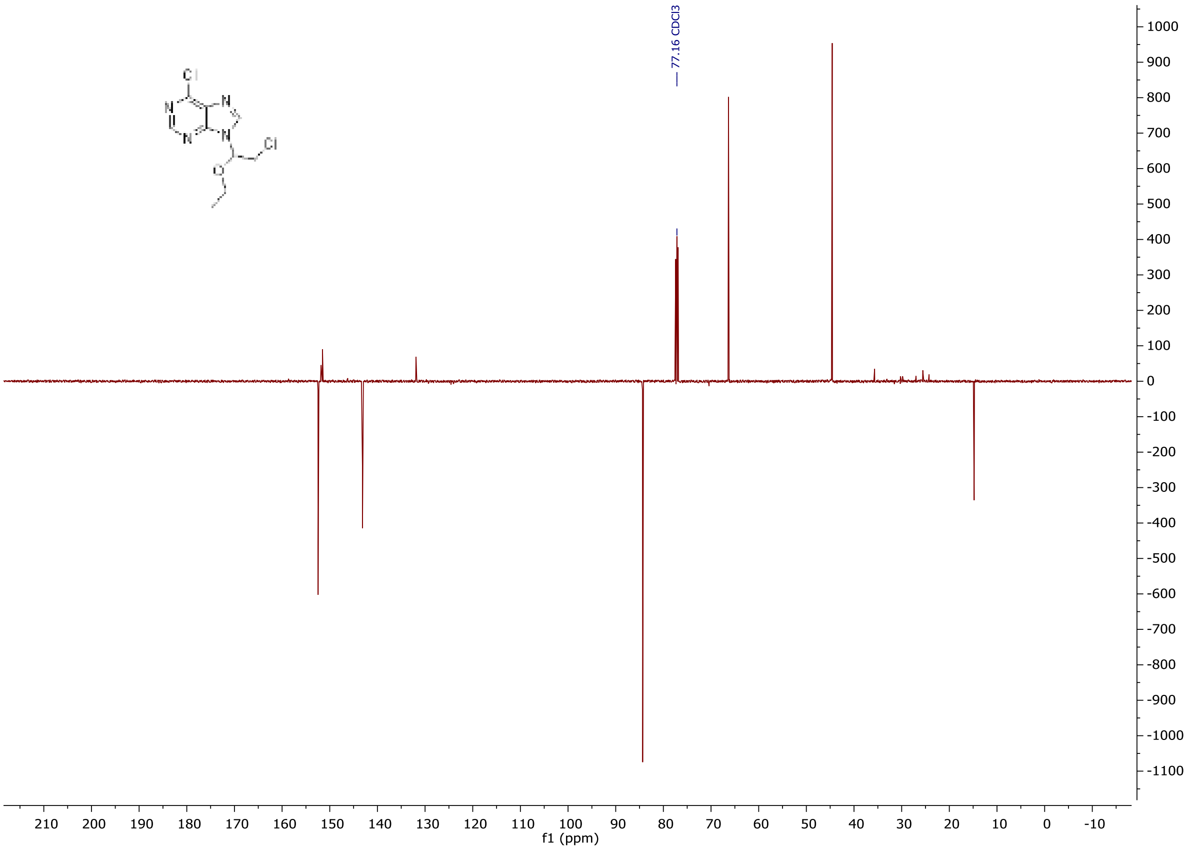

6-Chloro-9-(1-ethoxyprop-2-yn-1-yl)-2-fluoro-9*H*-purine (**34**)

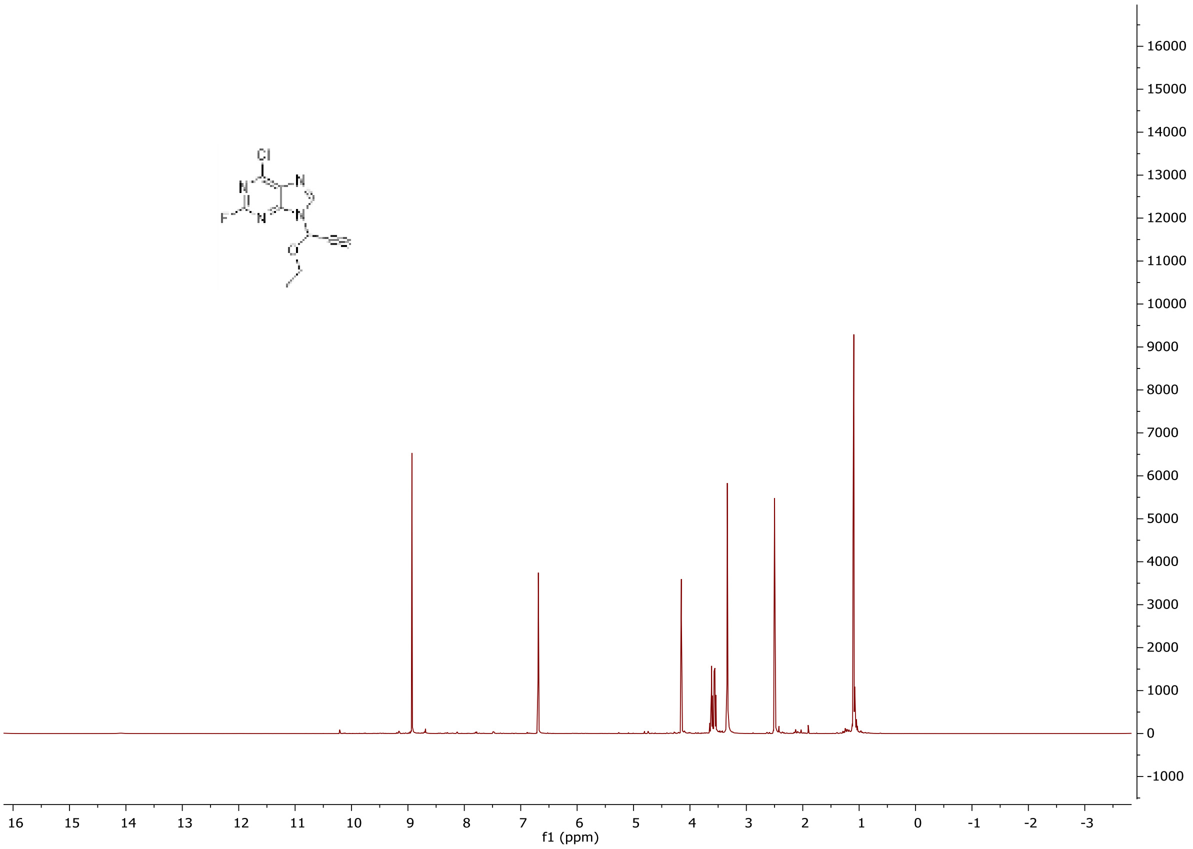

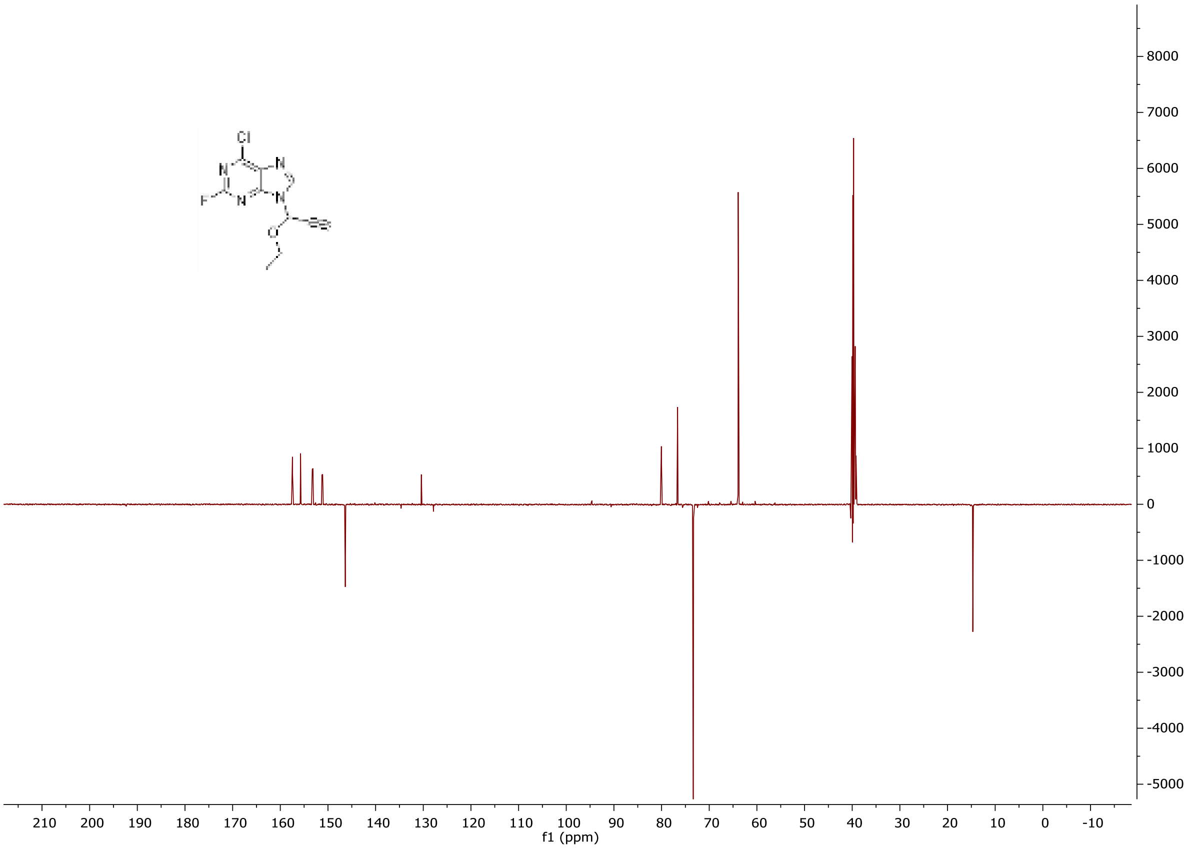

4-(Adenine-9-yl)-4-methoxybutan-2-one (**35**)

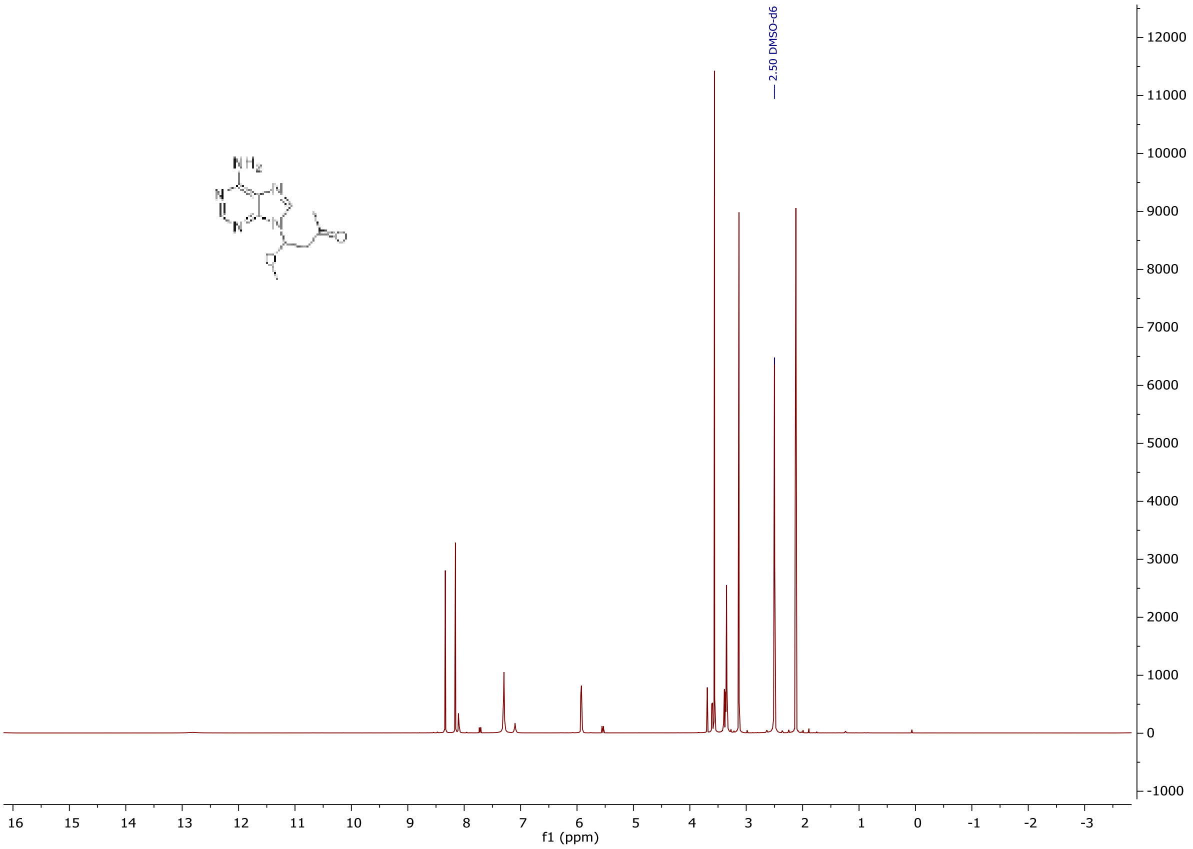

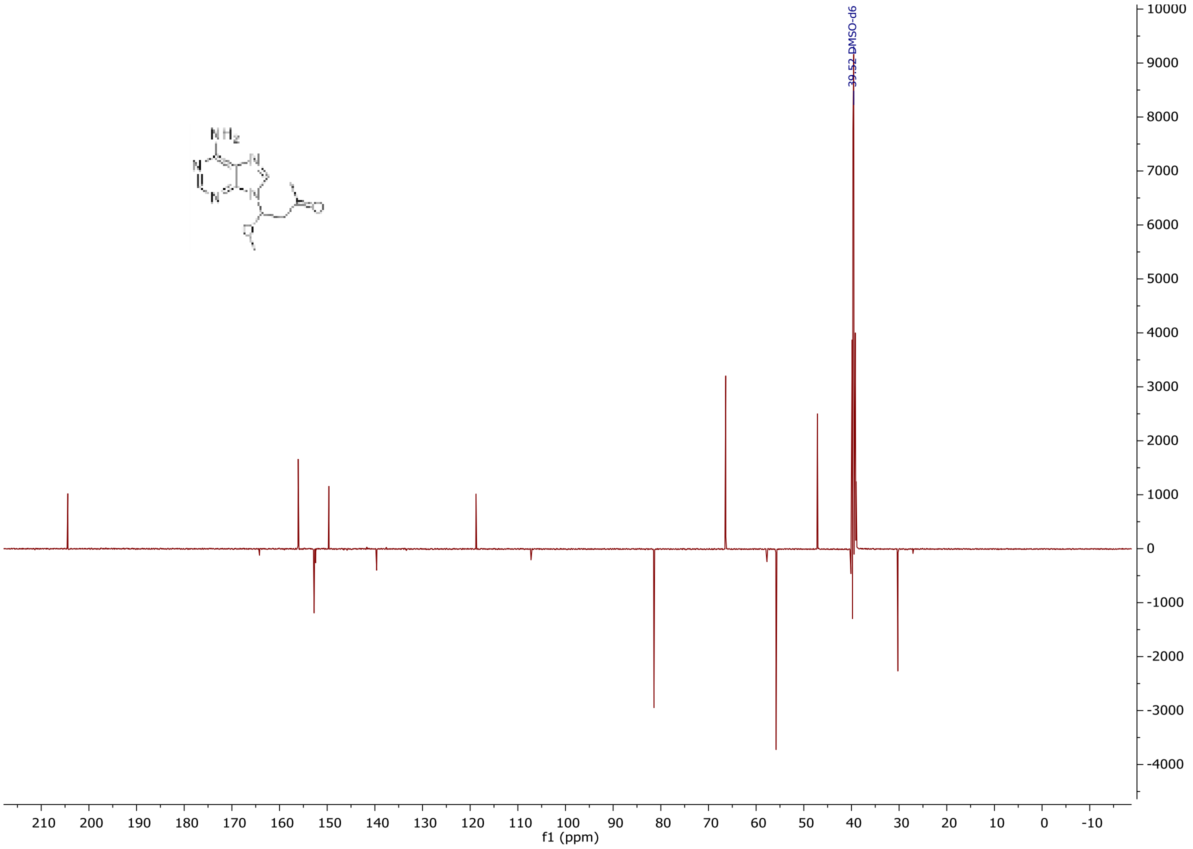

N,*N'*-(9-(1-Ethoxyprop-2-yn-1-yl)-9*H*-purine-2,6-diyl)diacetamide (**36**)

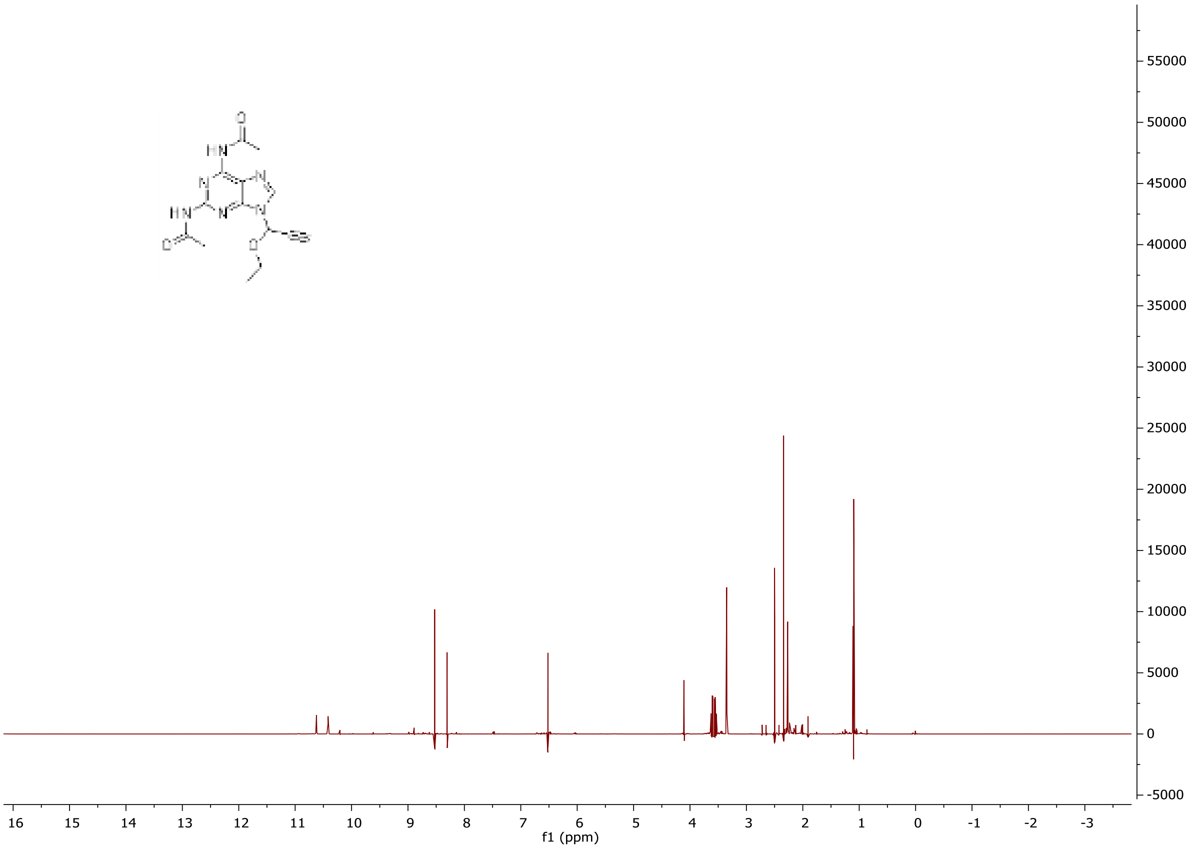

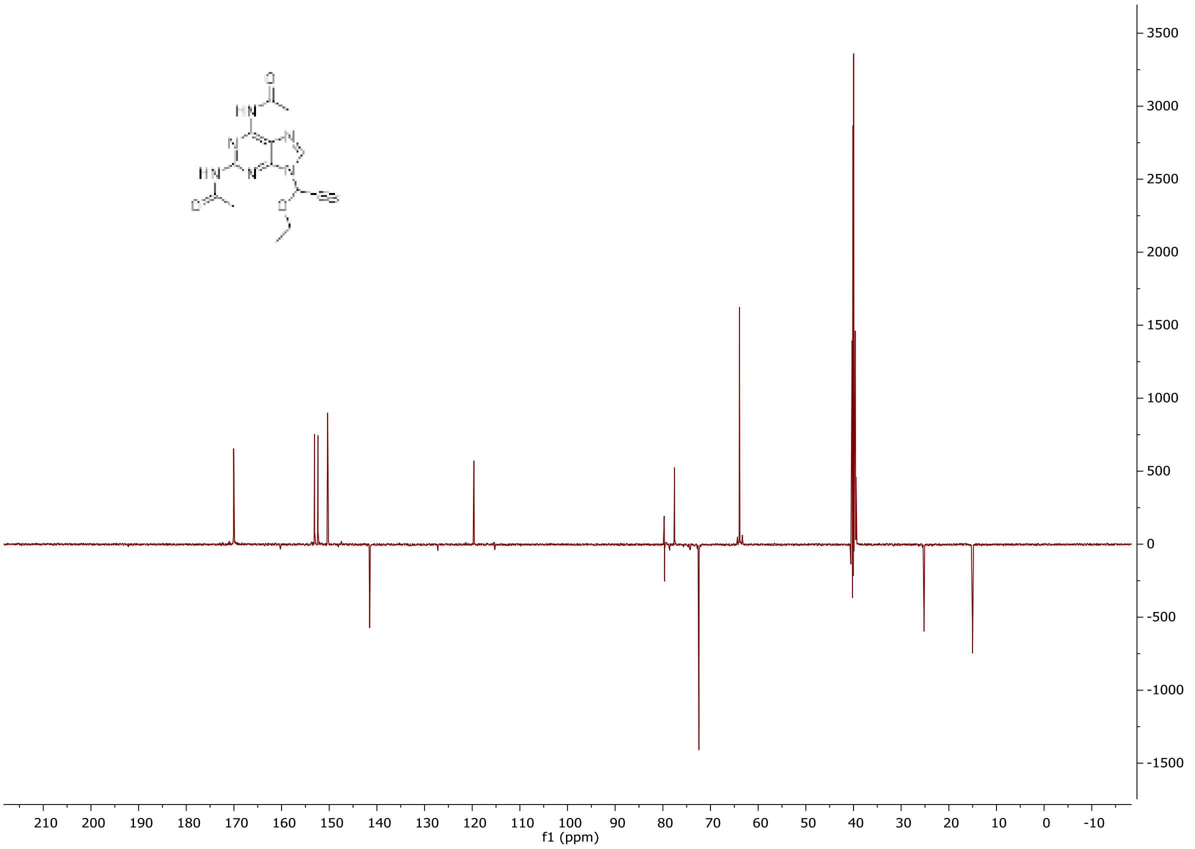

2-(4-Chloro-1*H*-pyrazolo[4,3-*c*]pyridin-1-yl)-2-ethoxyethyl acetate (**37**)

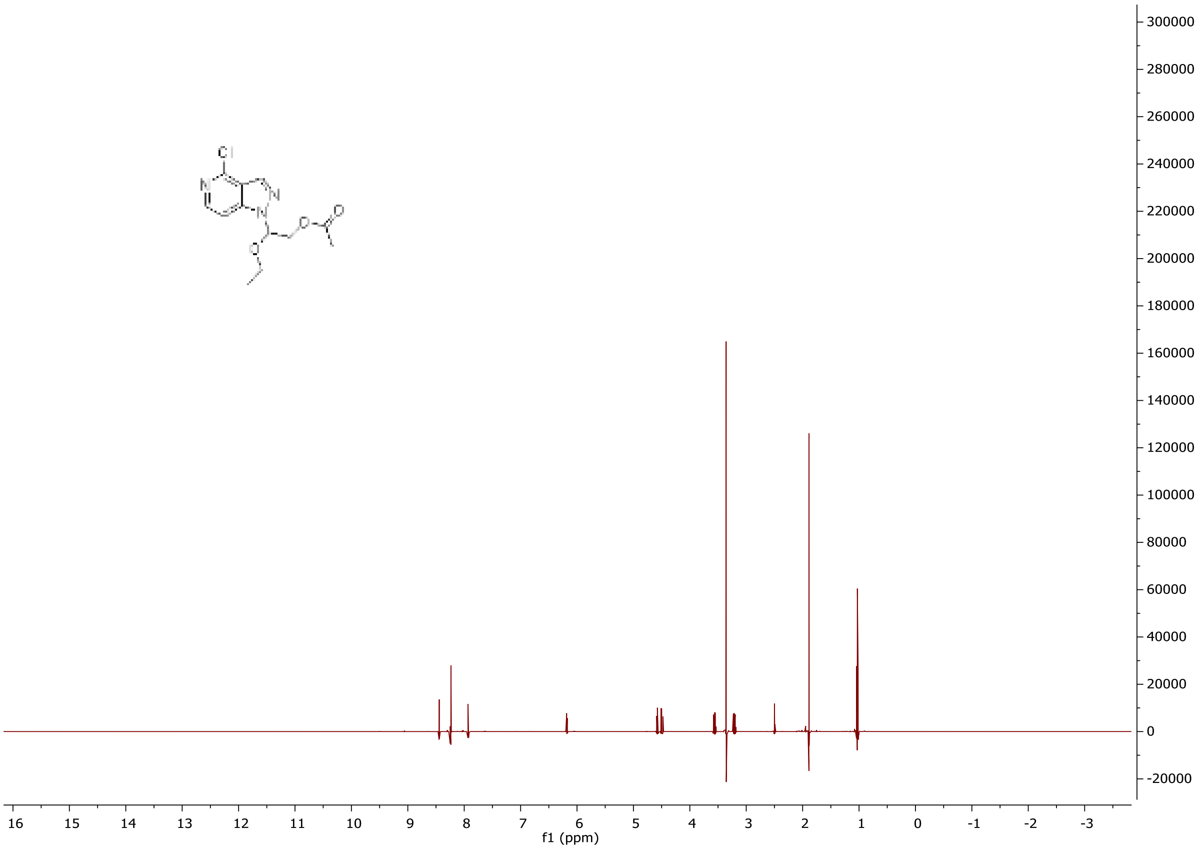

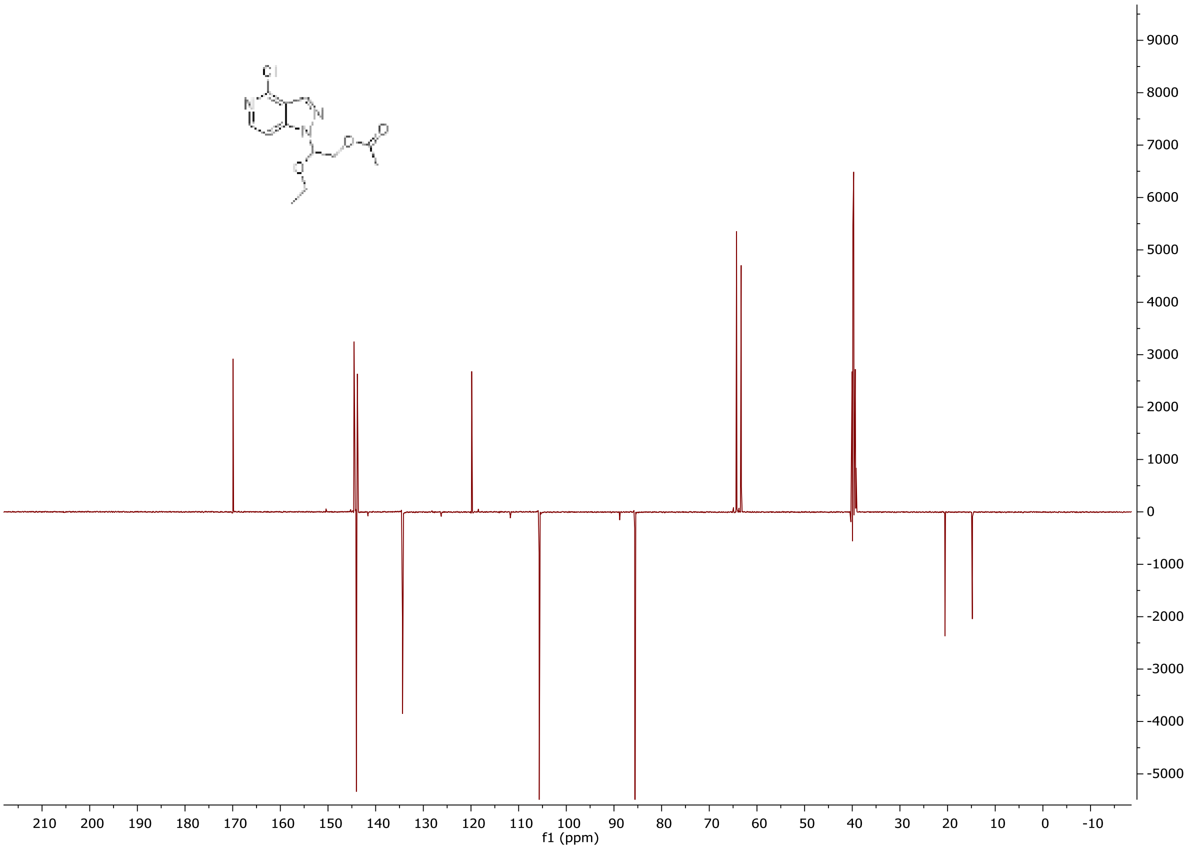

Ethyl 4-(4-chloro-1*H*-imidazo[4,5-*c*]pyridin-1-yl)-2-cyano-4-ethoxybutanoate (**38**)

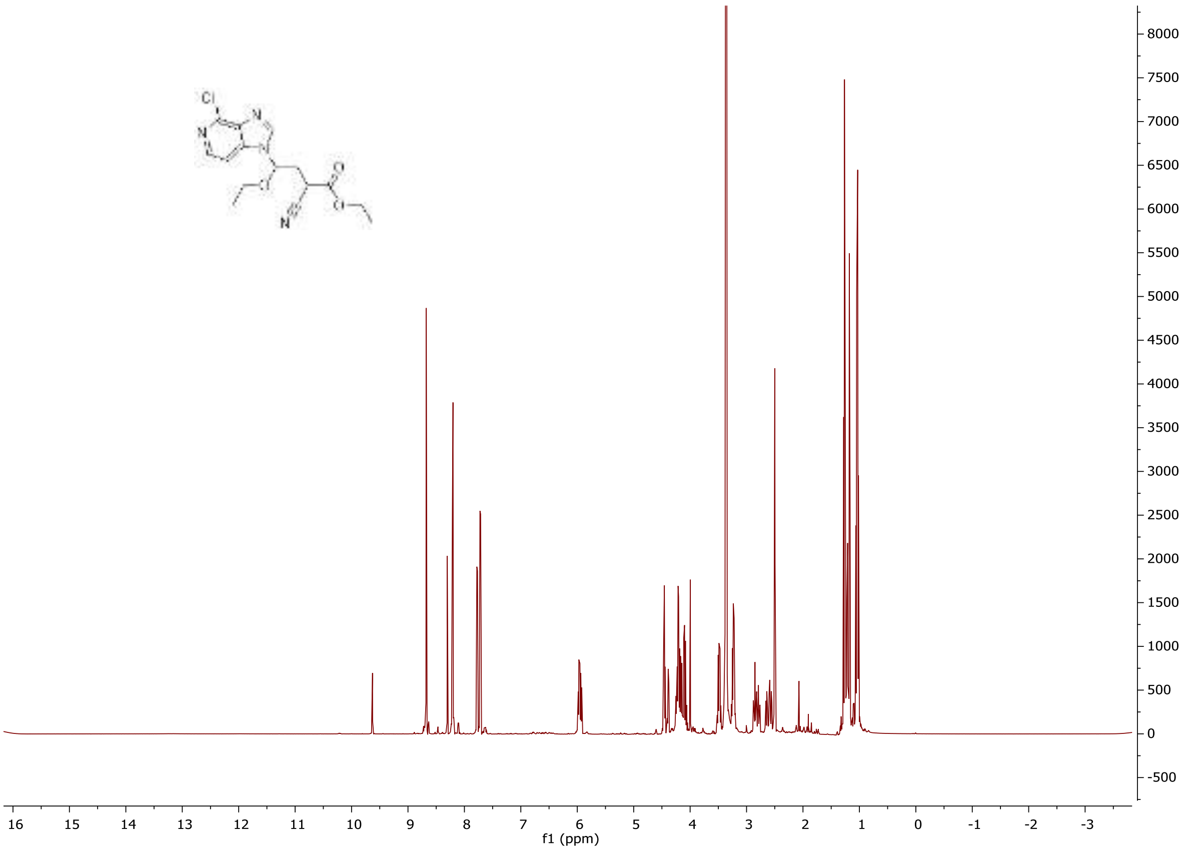

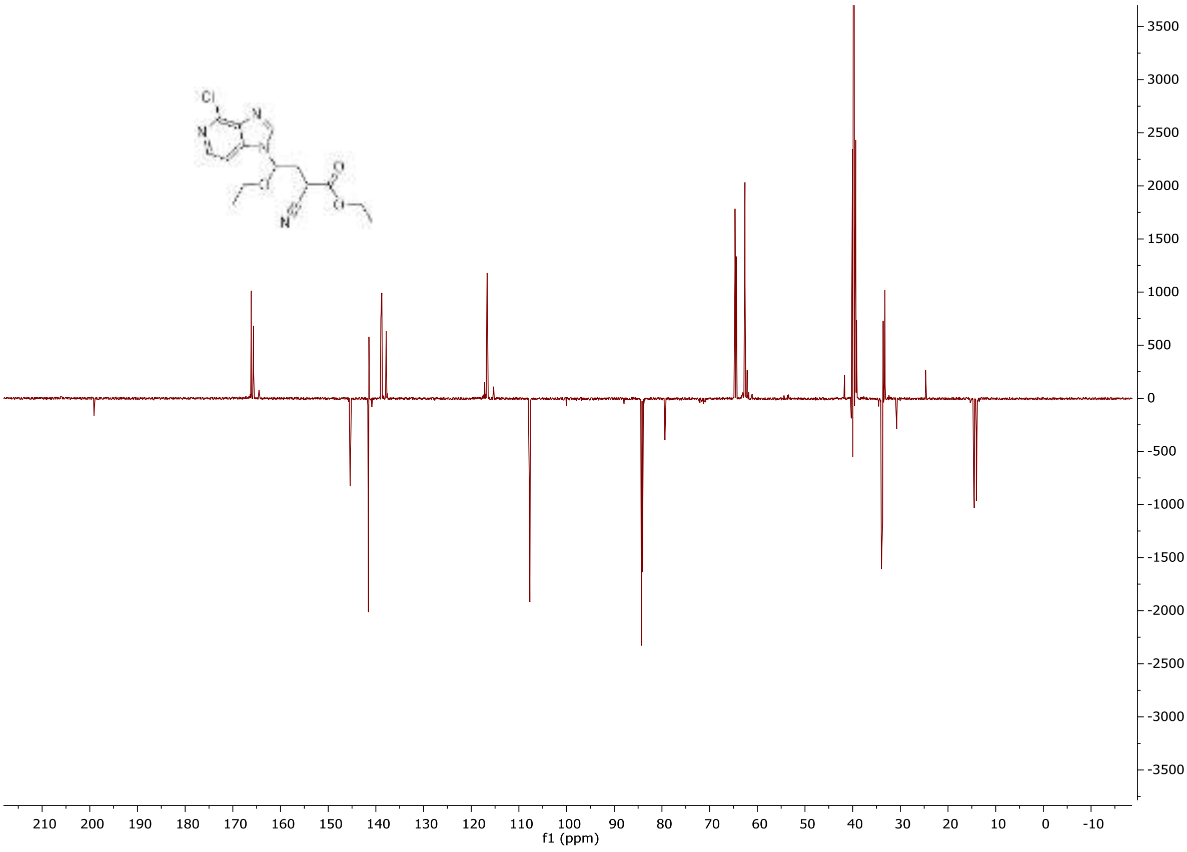

2-((1*H*-1,2,4-Triazol-1-yl)methoxy)ethyl acetate (**39**)

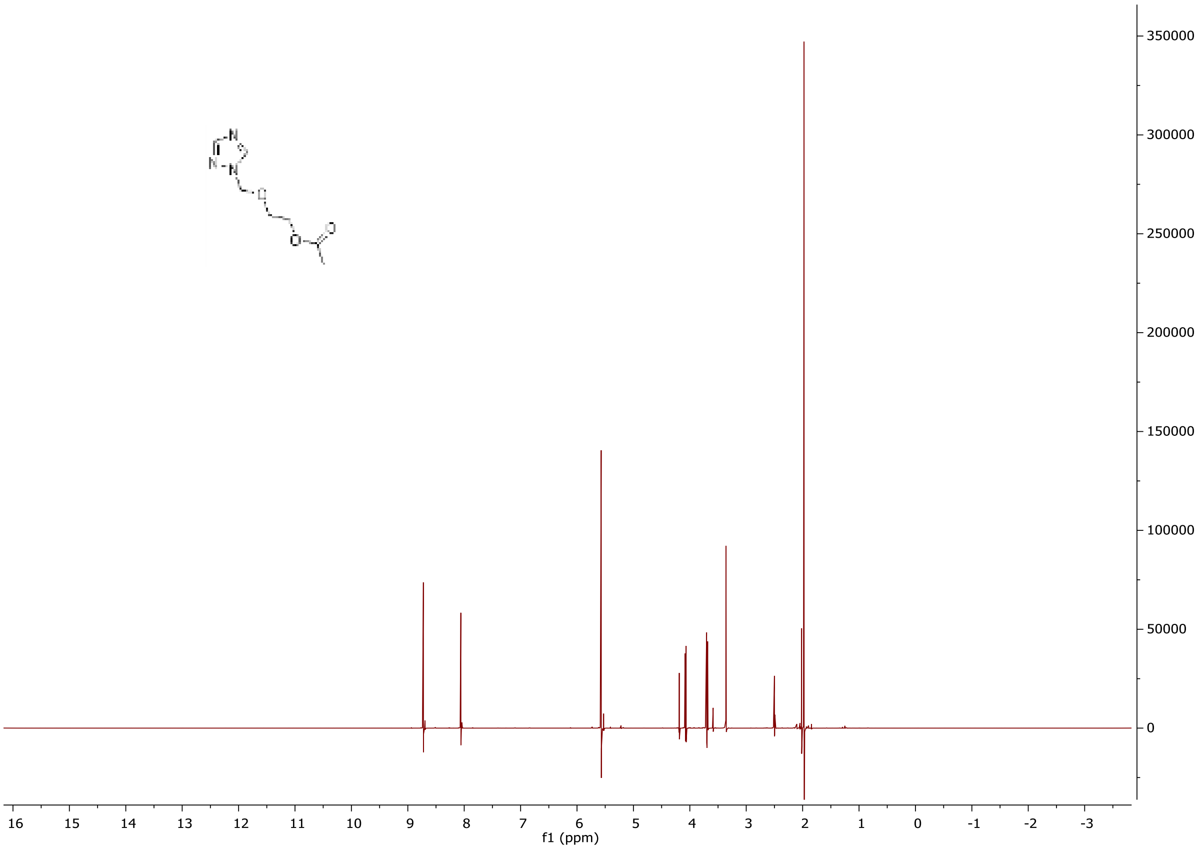

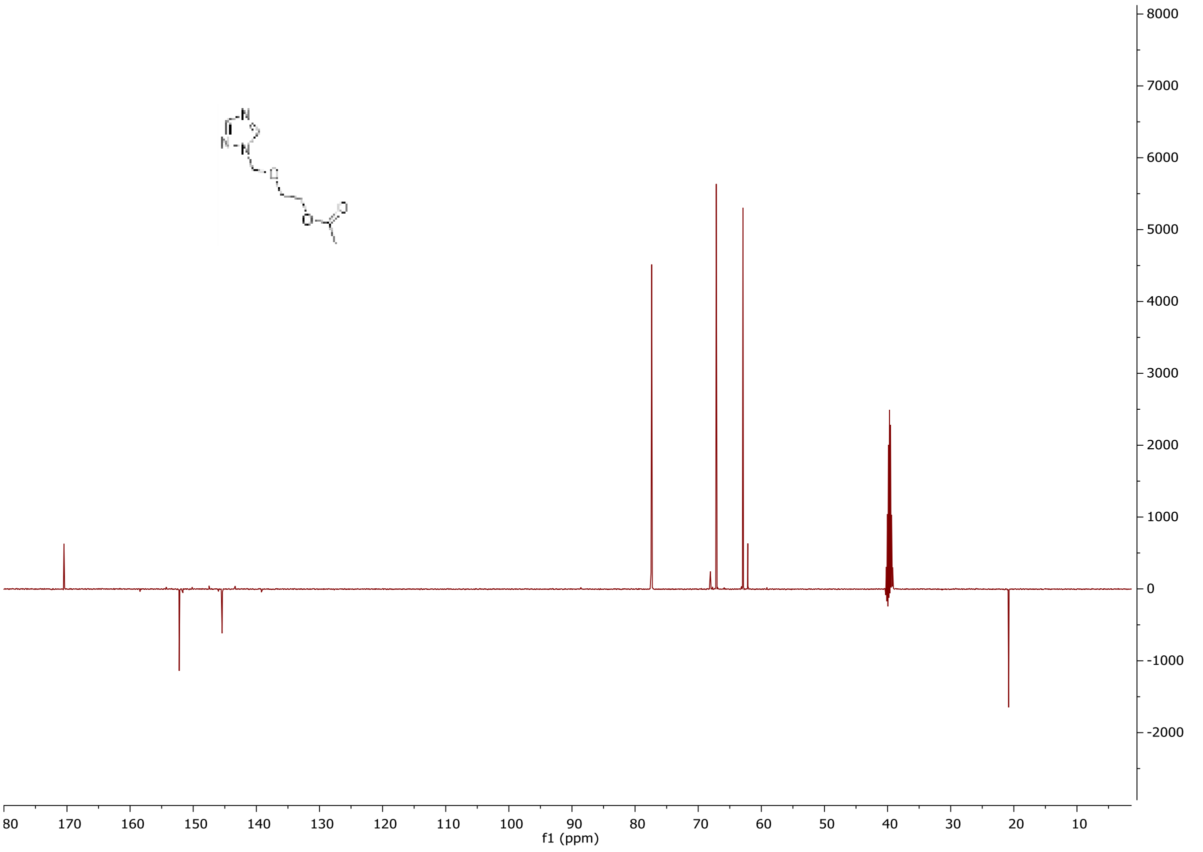

3-((2-Acetoxyethoxy)methyl)thiazol-3-ium trifluoromethanesulfonate (**40**)

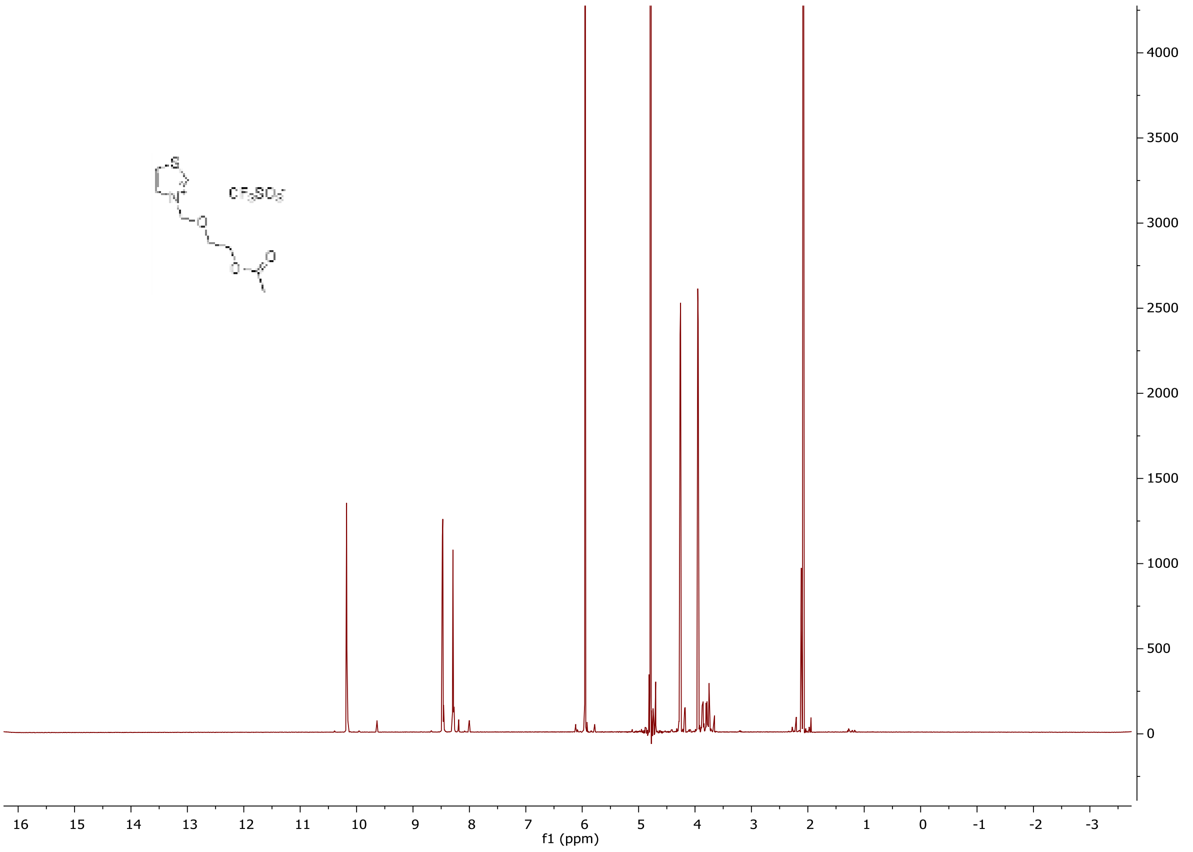

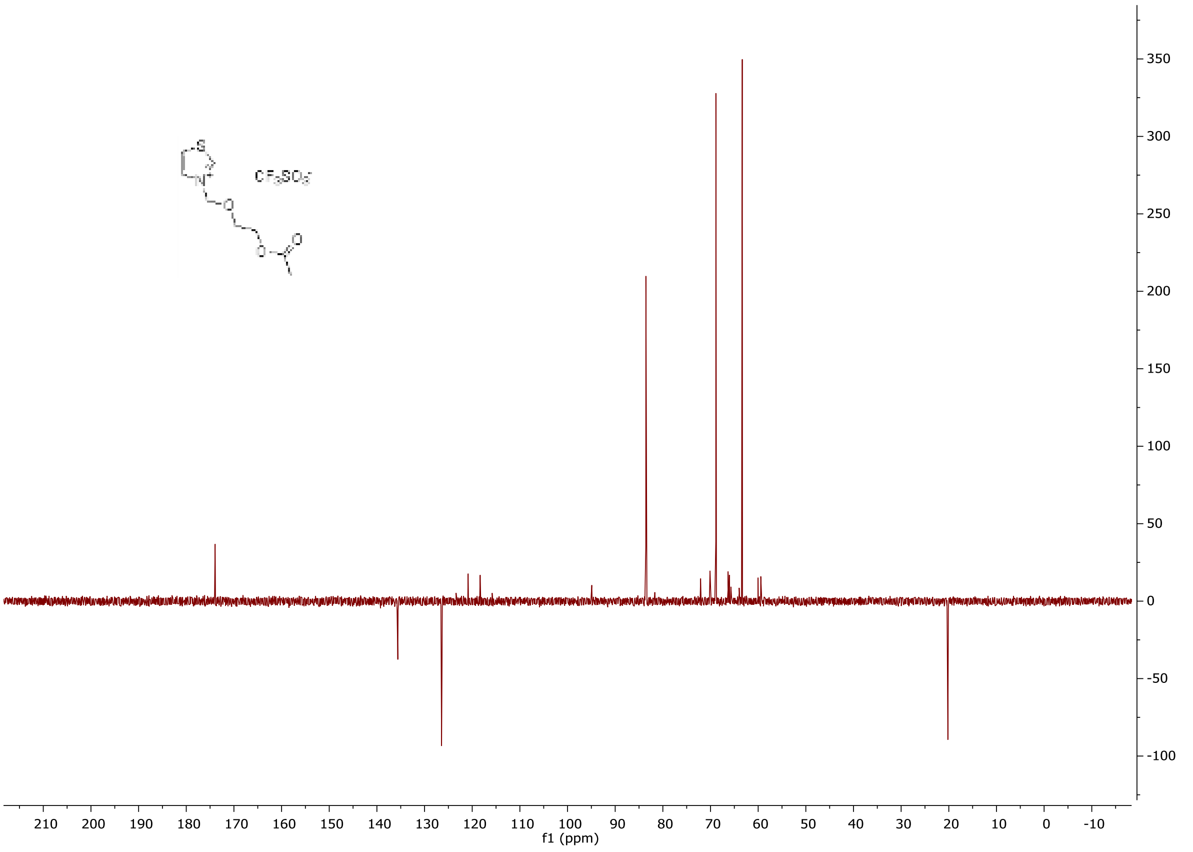

1-(((4-Acetoxytetrahydrofuran-3-yl)oxy)methyl)-4-nitro-1*H*-pyrazole-3-carboxylic acid (**41**)

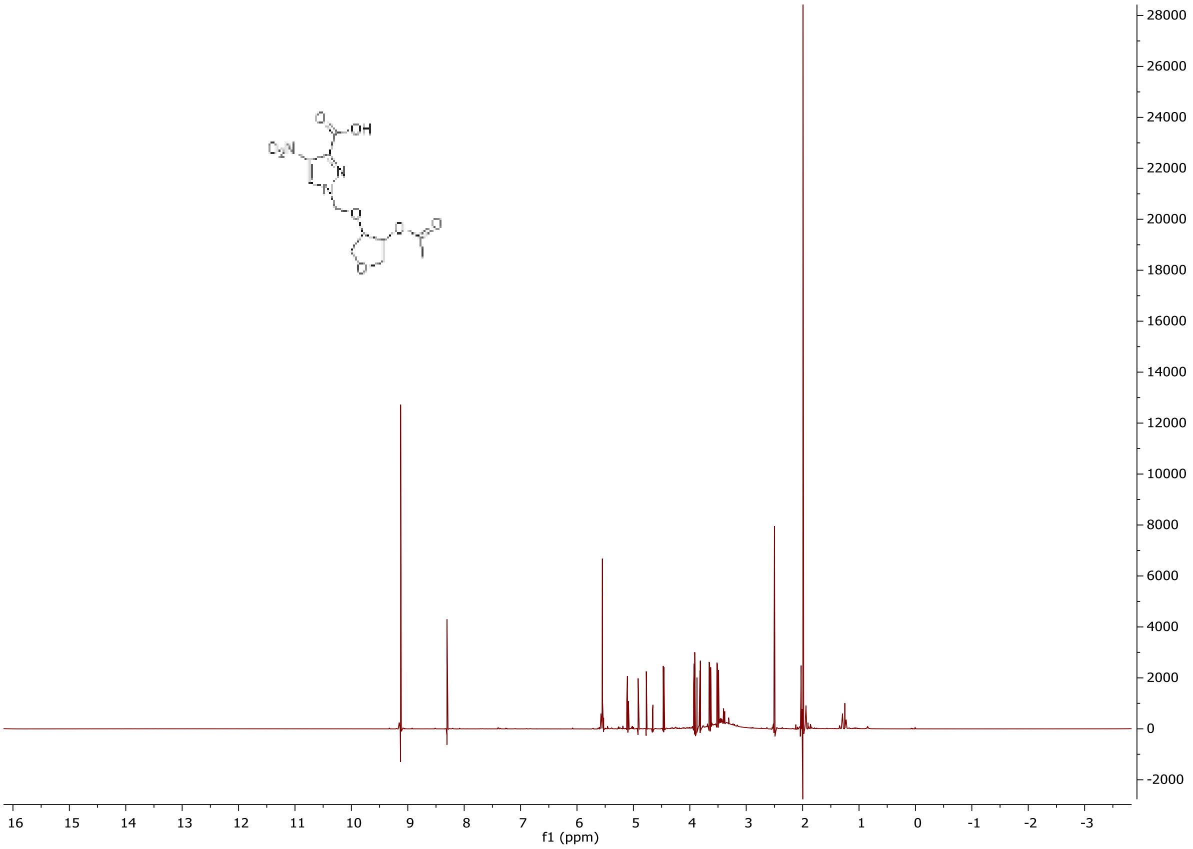

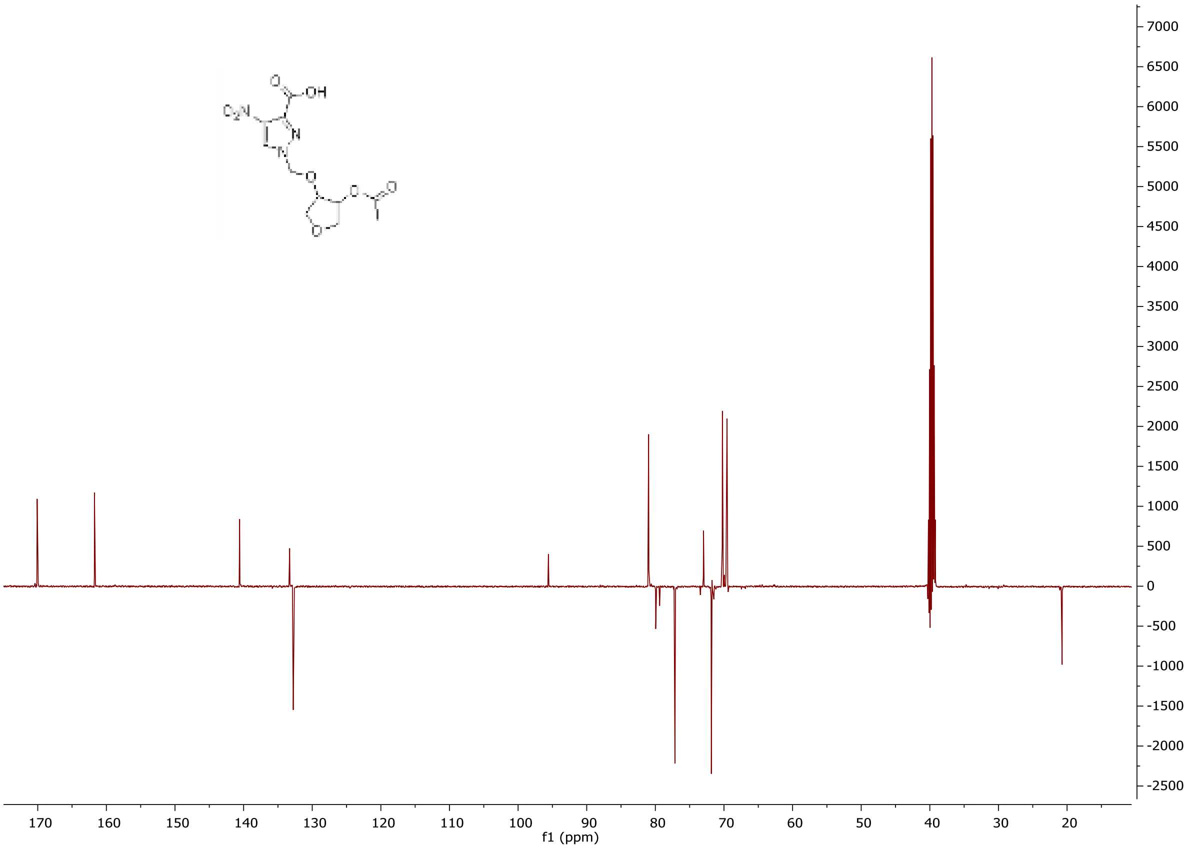

2-((5-Phenyl-1*H*-tetrazol-1-yl)methoxy)but-3-en-1-yl acetate (**42**)

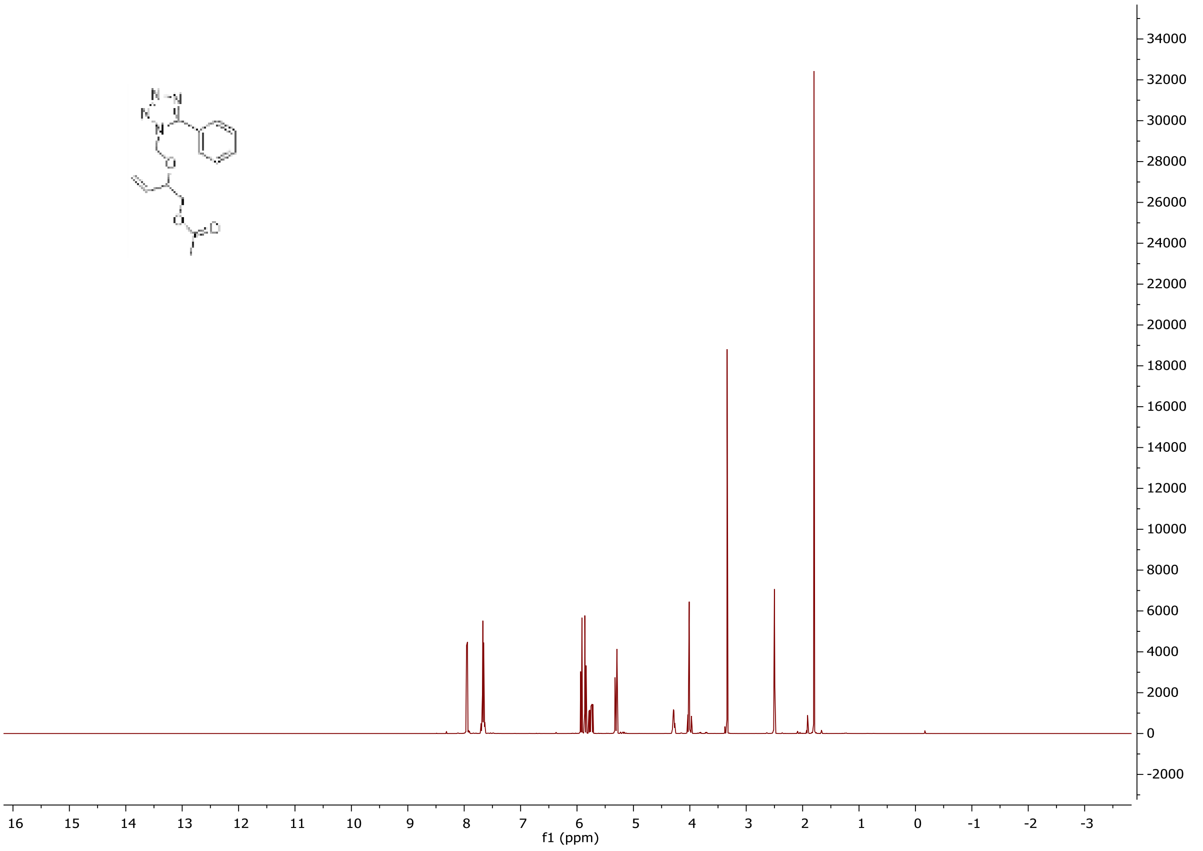

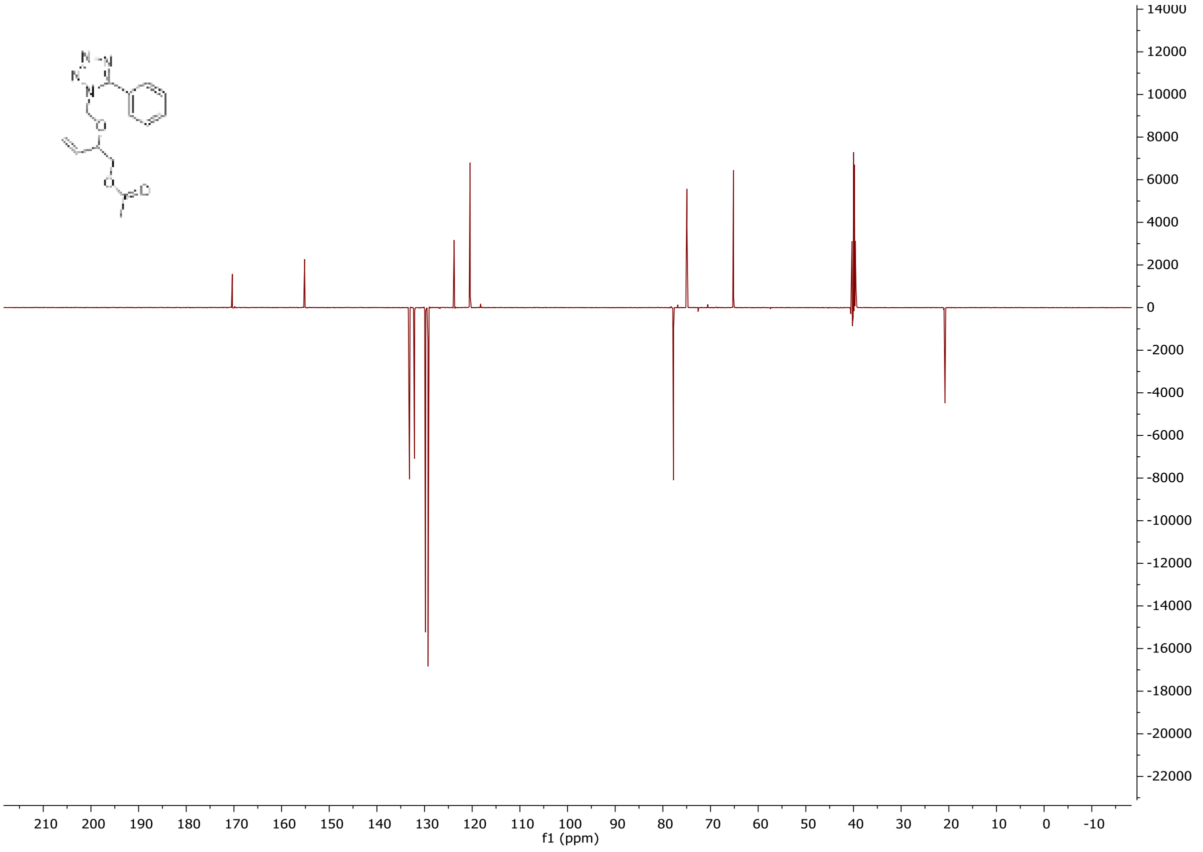

2-((5-Phenyl-2*H*-tetrazol-2-yl)methoxy)but-3-en-1-yl acetate (**43**)

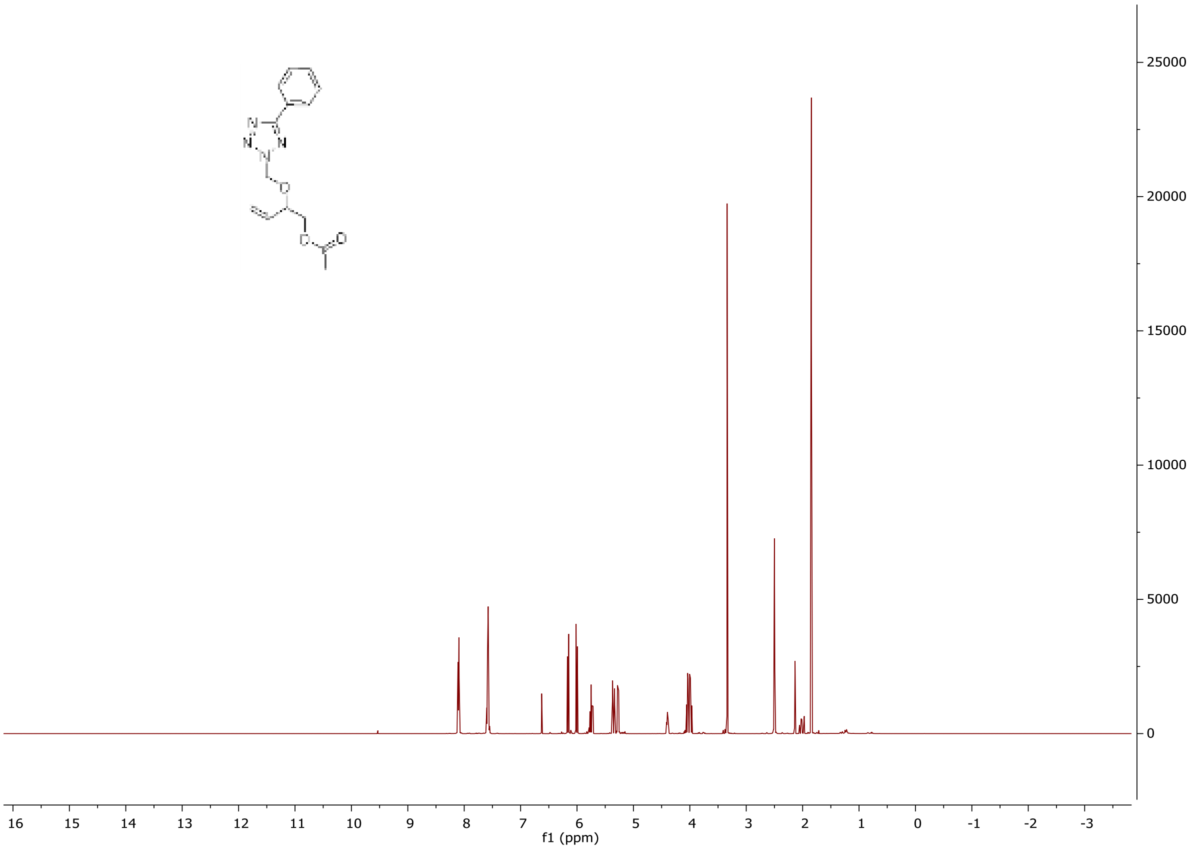

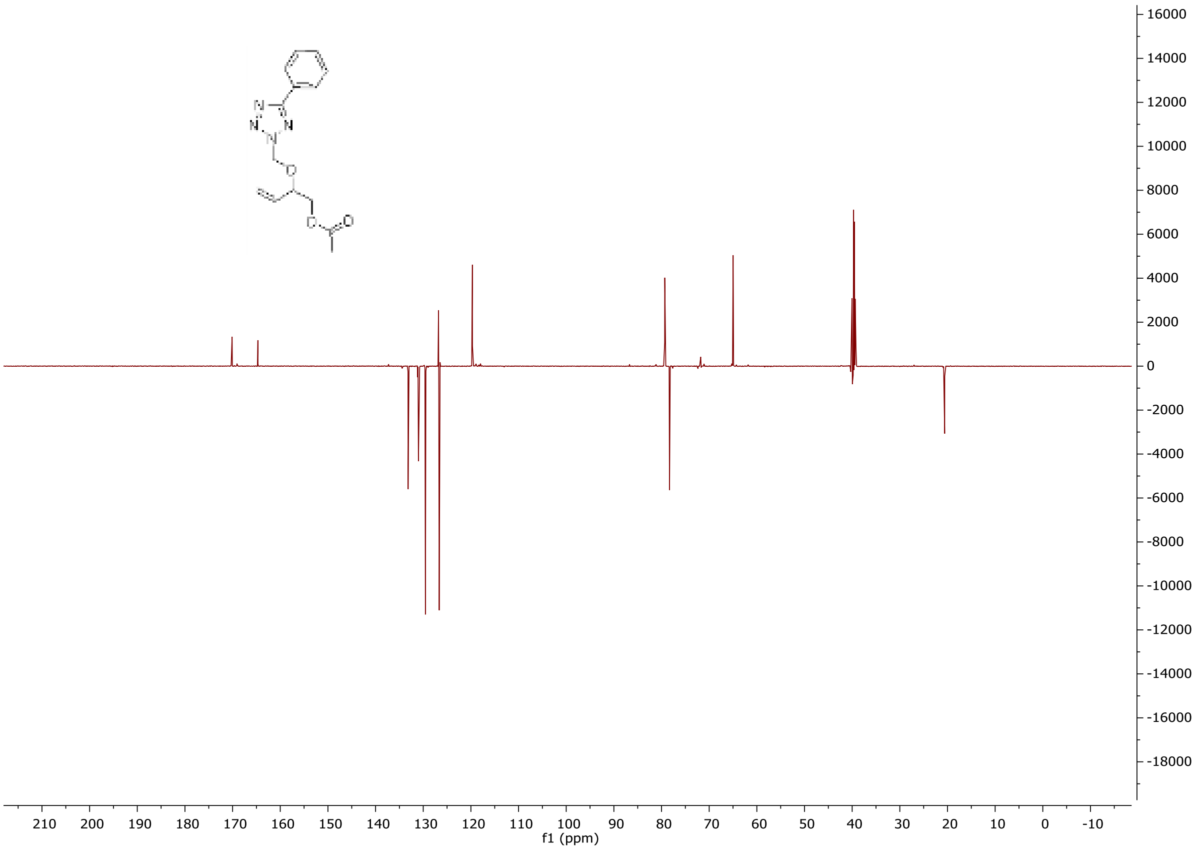

4-(1*H*-Benzo[*d*][1,2,3]triazol-1-yl)-4-methoxybutanenitrile (**45**)

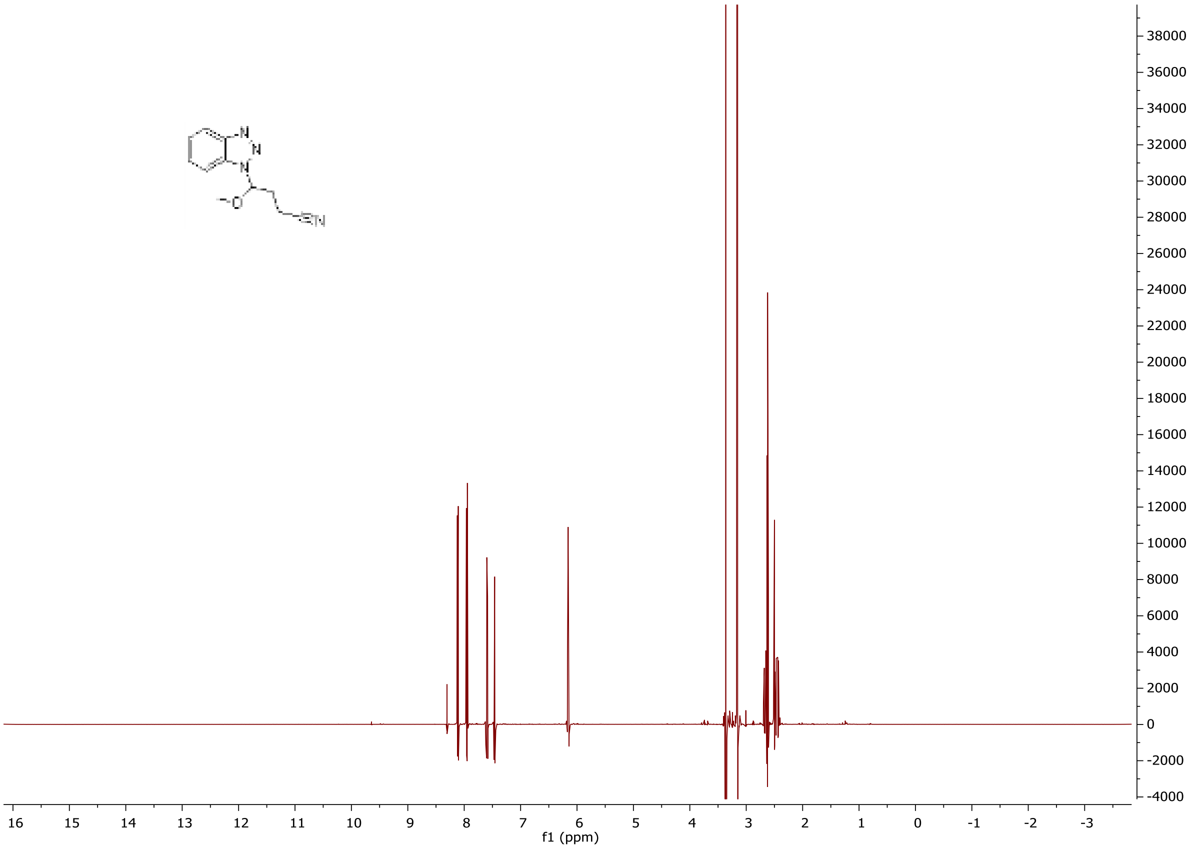

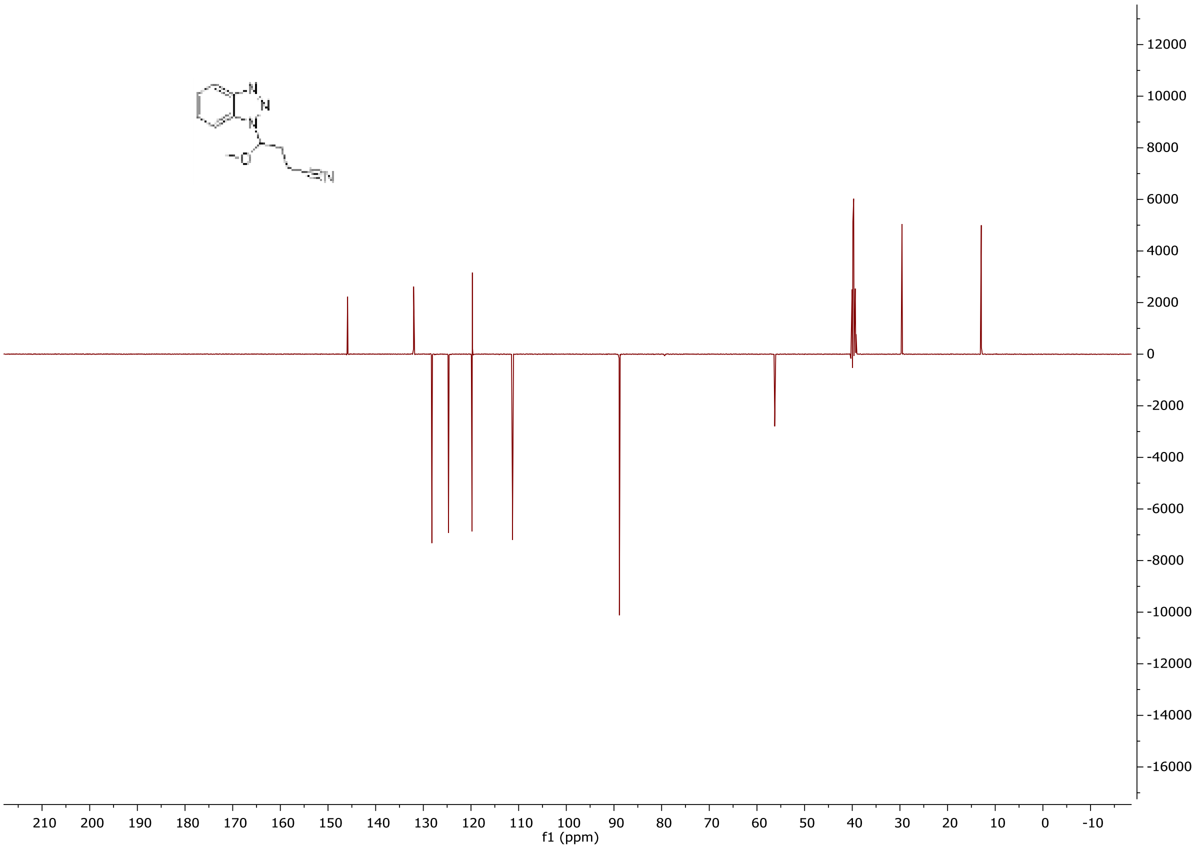

2-(2-Bromo-1-(6-chloro-9*H*-purin-9-yl)ethoxy)ethyl pivalate (**46**)

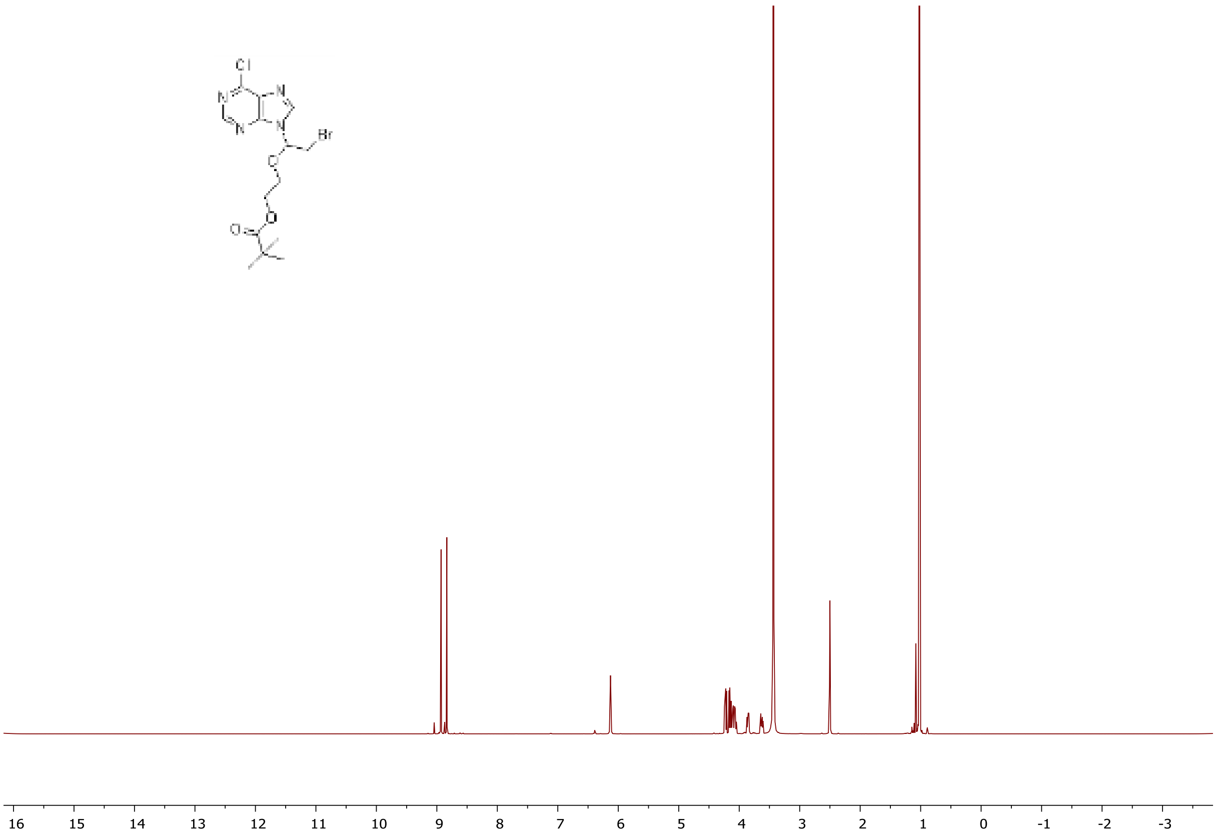

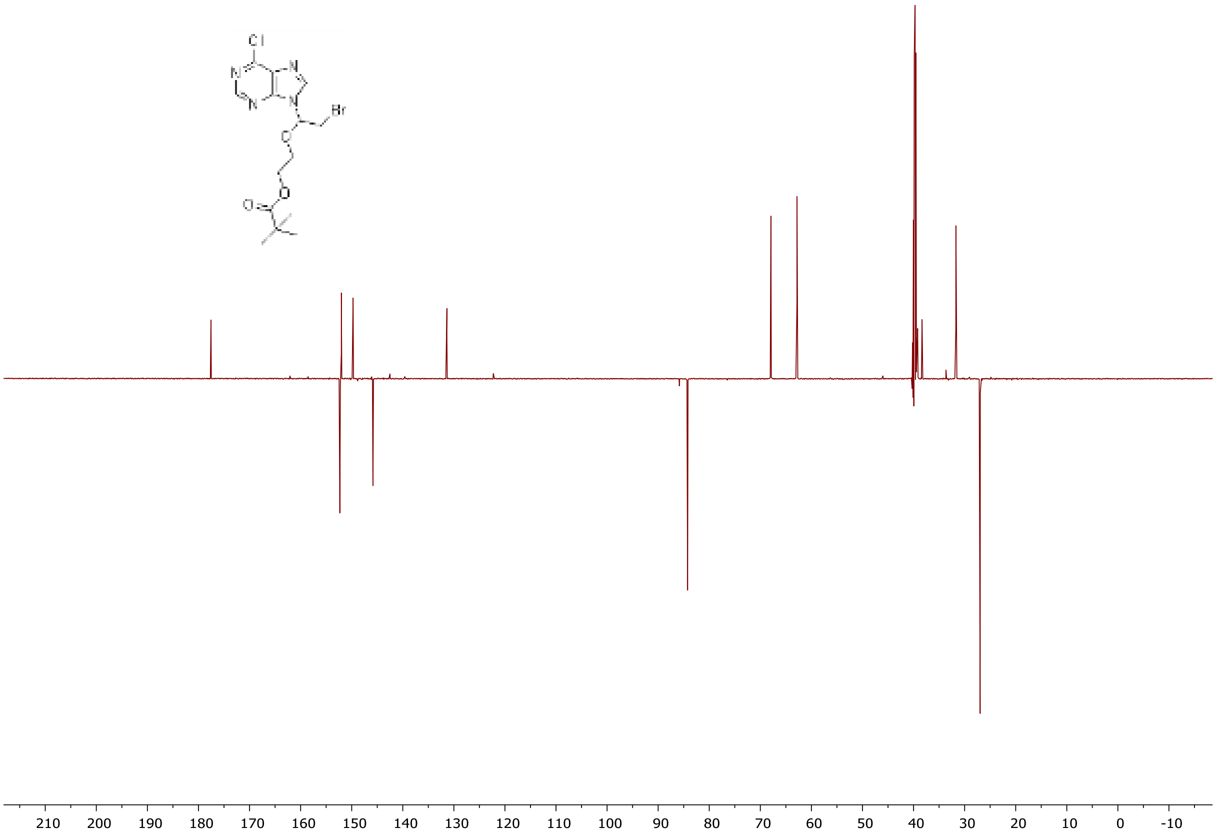

2-(2-Bromo-1-(6-chloro-9*H*-purin-9-yl)ethoxy)ethyl benzoate (**47**)

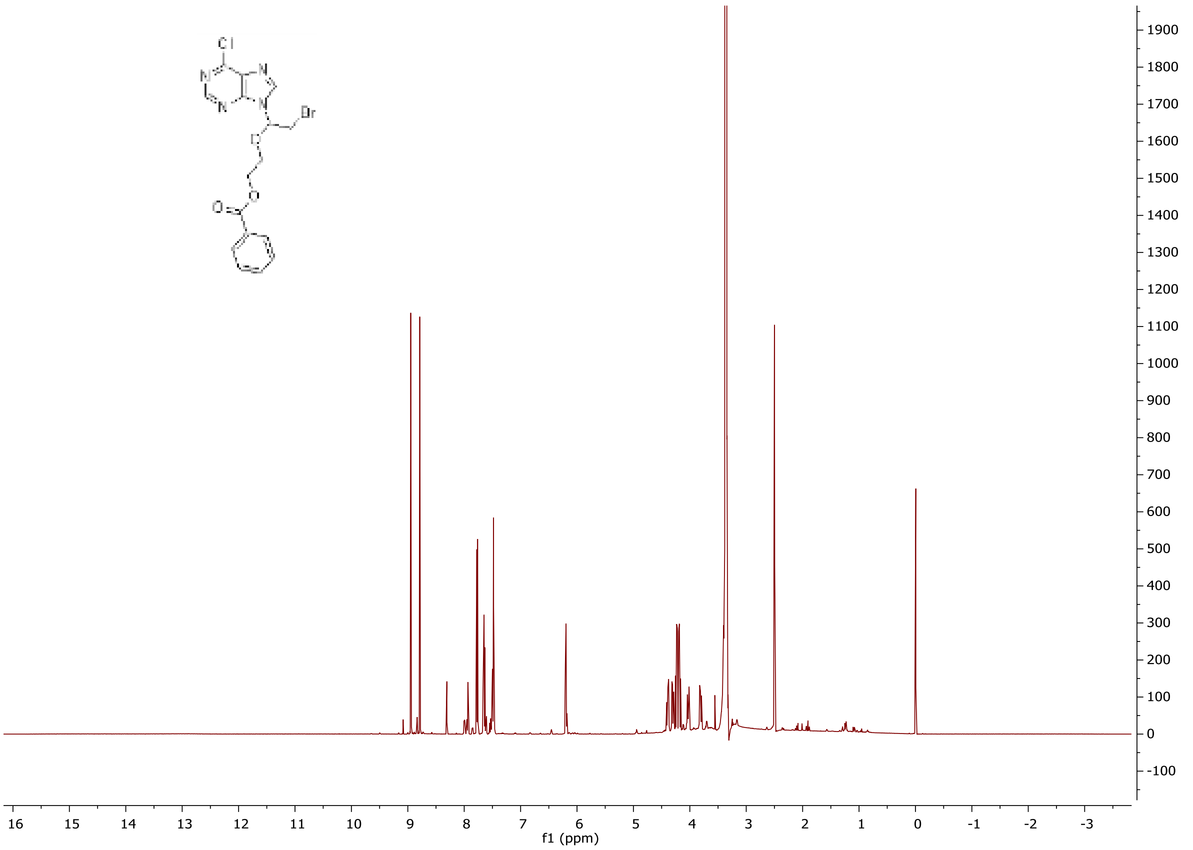

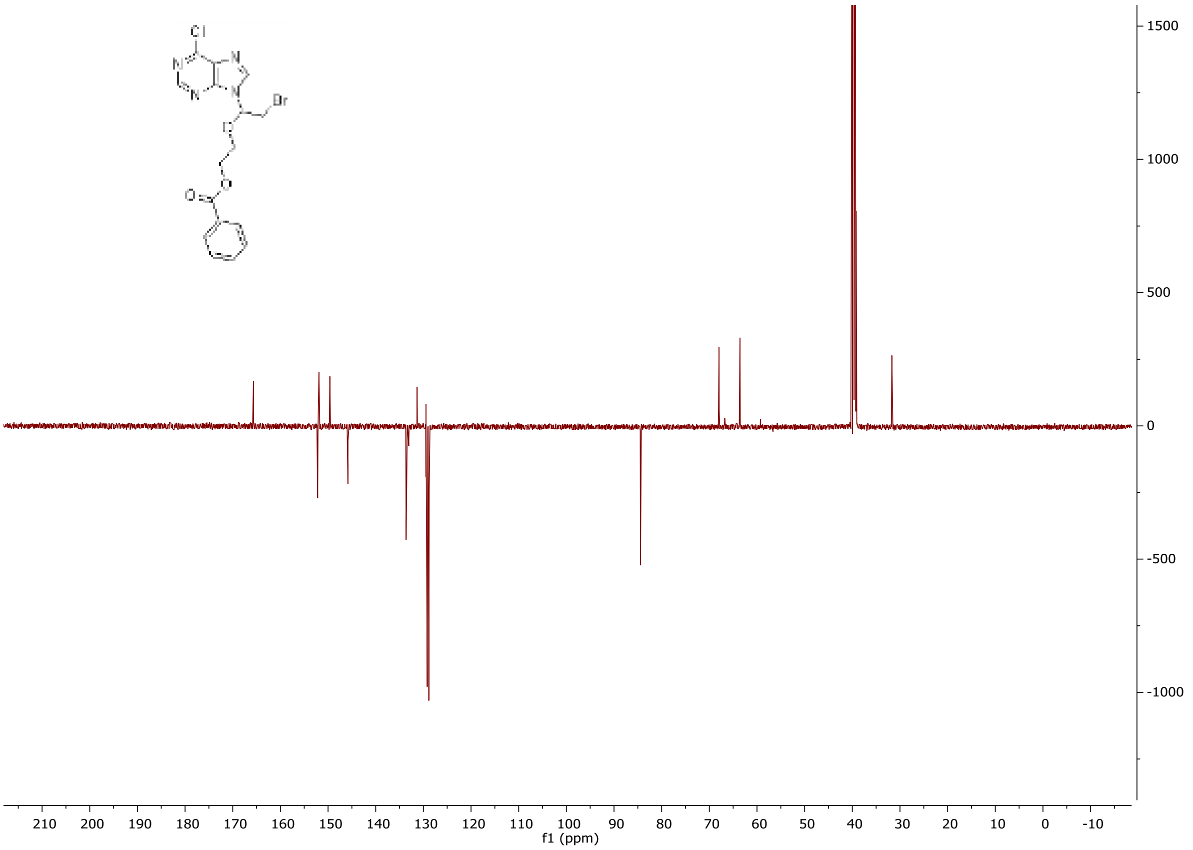

2-(2-Bromo-1-(6-chloro-9*H*-purin-9-yl)ethoxy)ethyl (2*S*)-2-(6-methoxynaphthalen-2-yl)-propanoate (**49**)

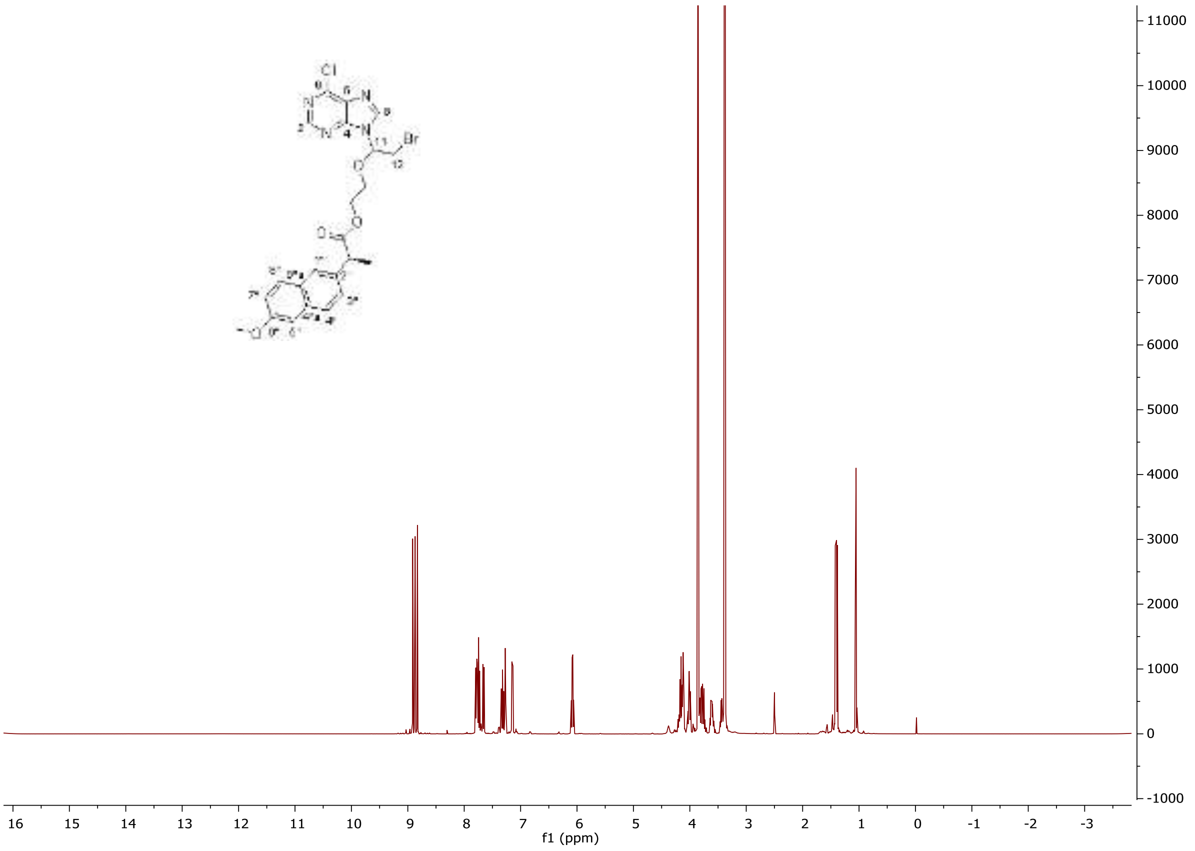

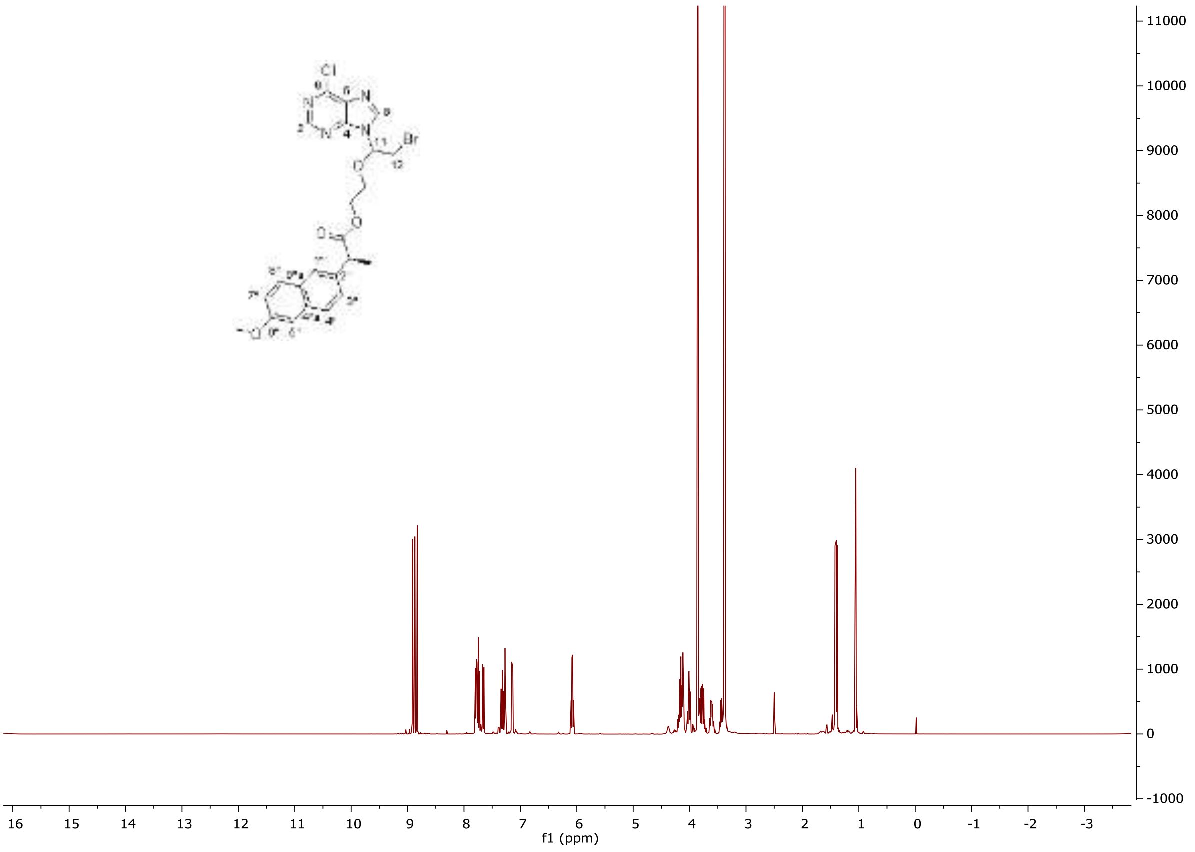

2-(2-Bromo-1-(6-chloro-9*H*-purin-9-yl)ethoxy)ethyl decanoate (**51**)

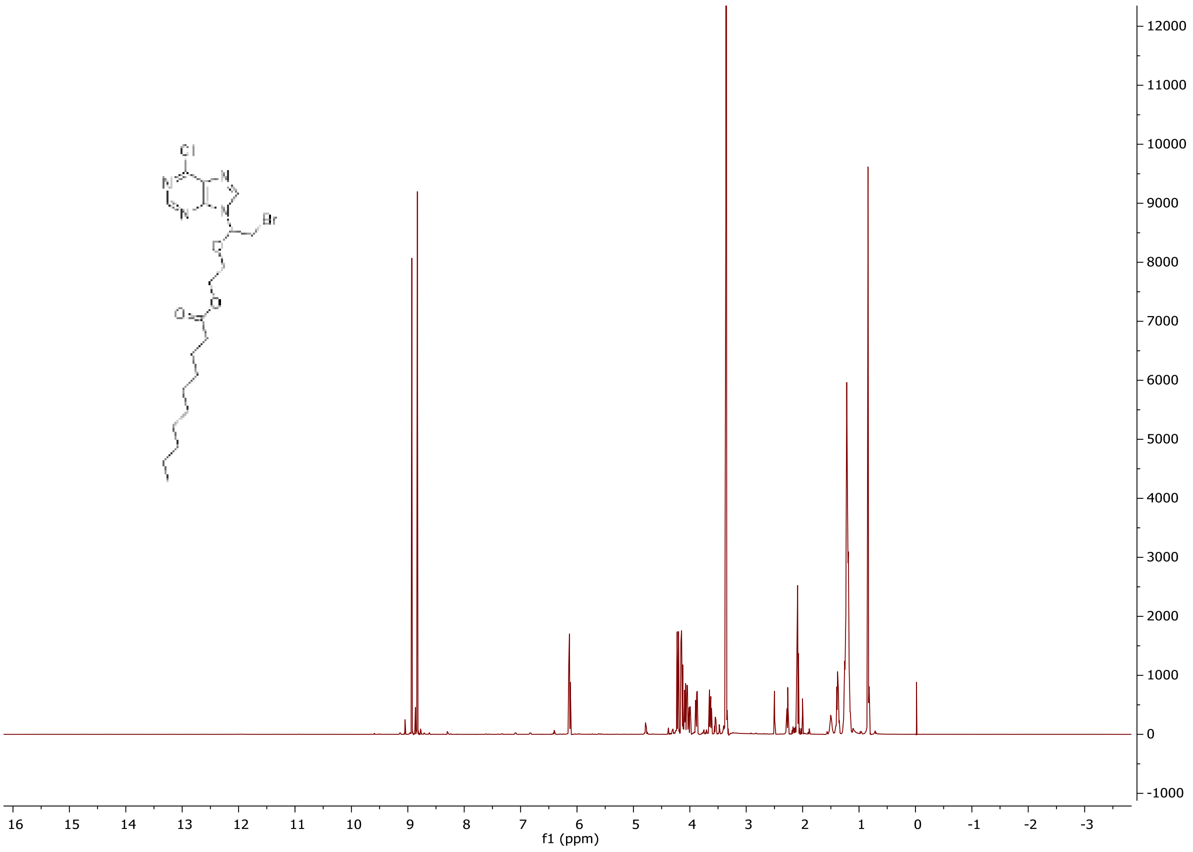

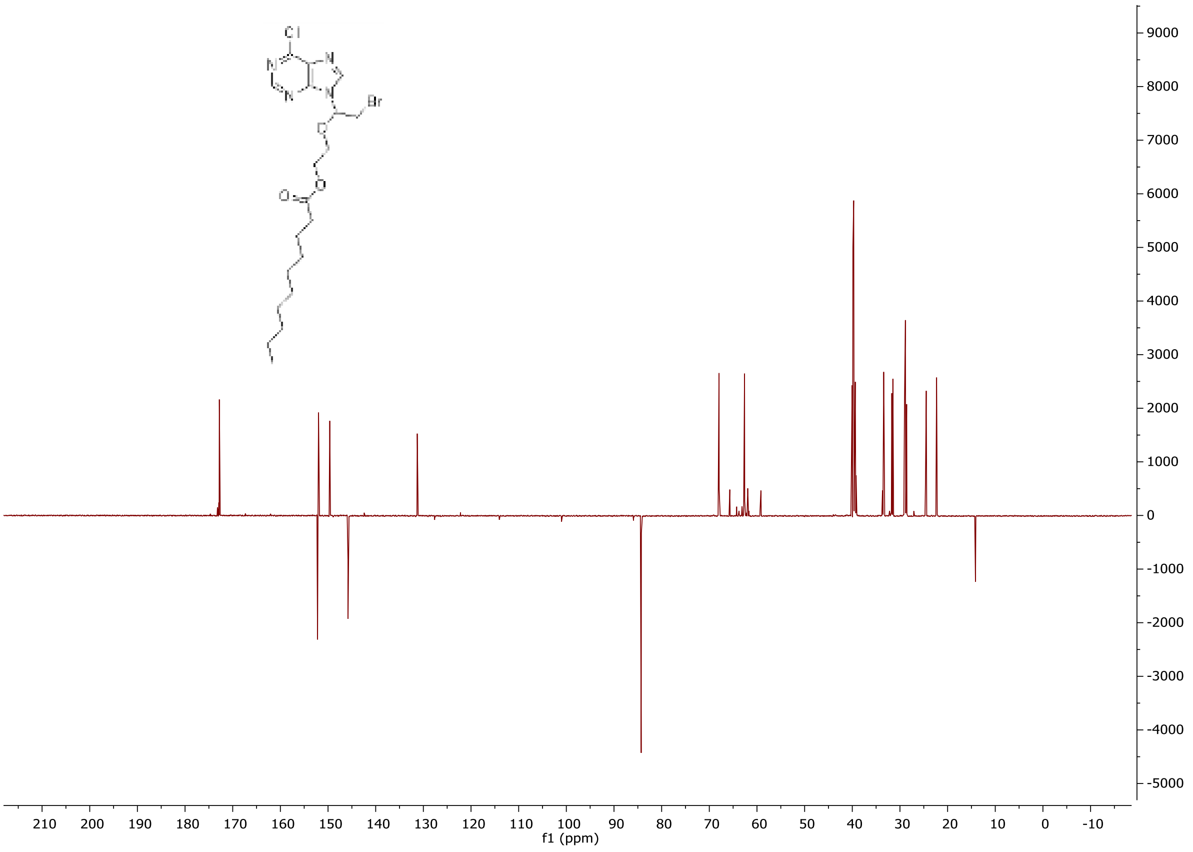

2-(1-Acetoxy-2-bromoethoxy)ethyl acetate (**52**)

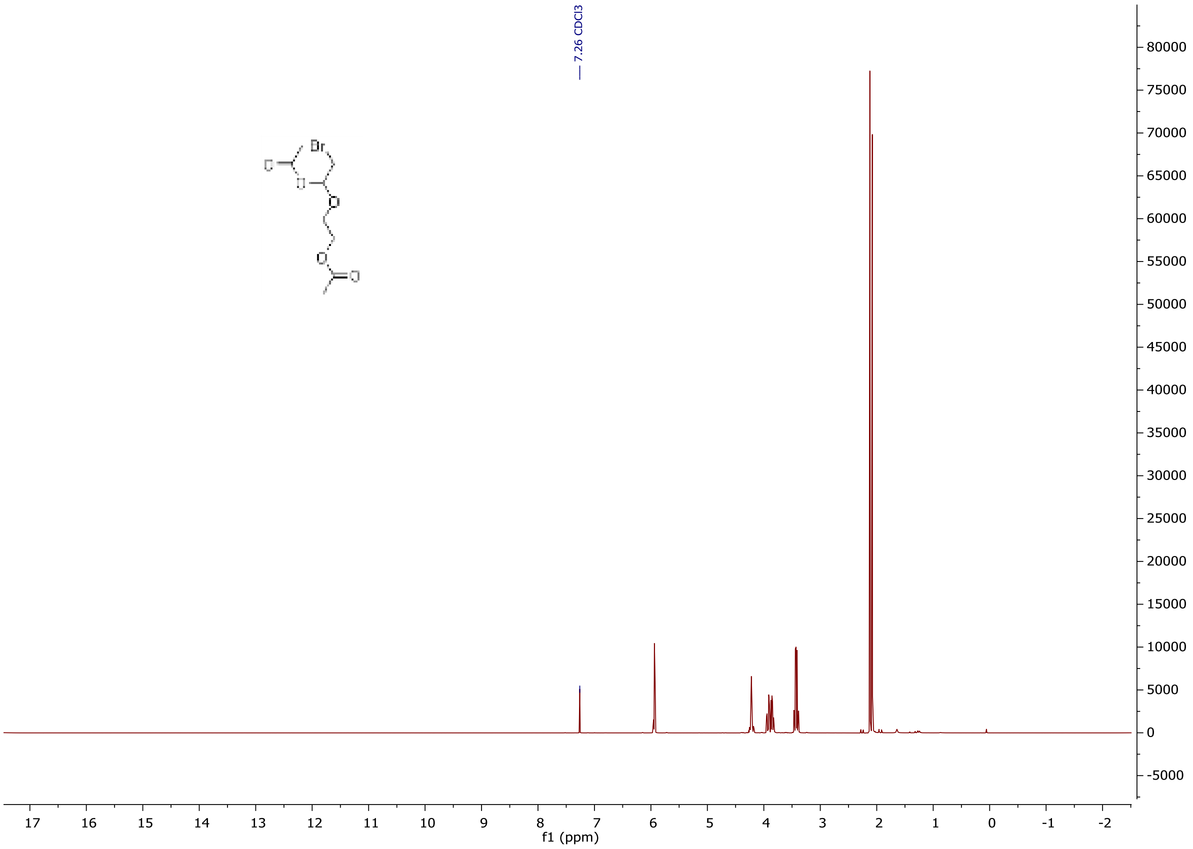

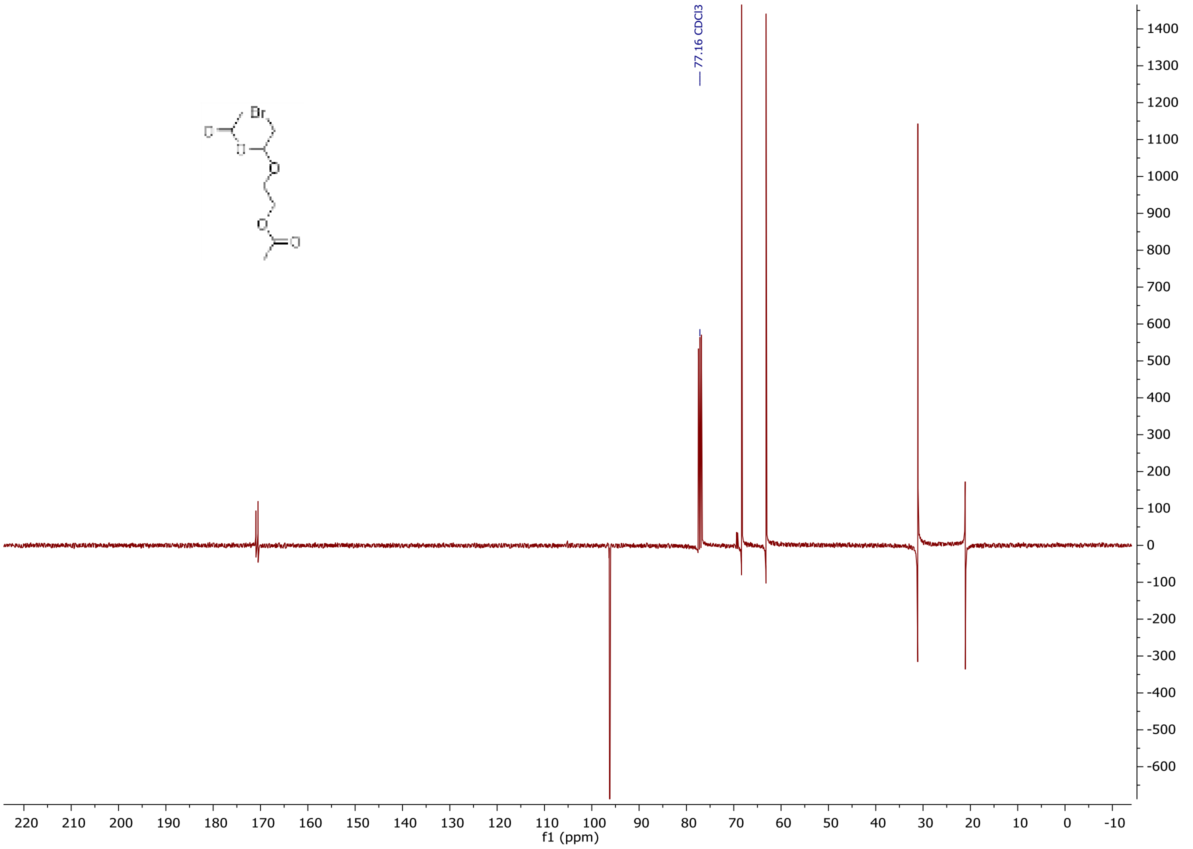

HMBC spectra of compounds 22 and 42:

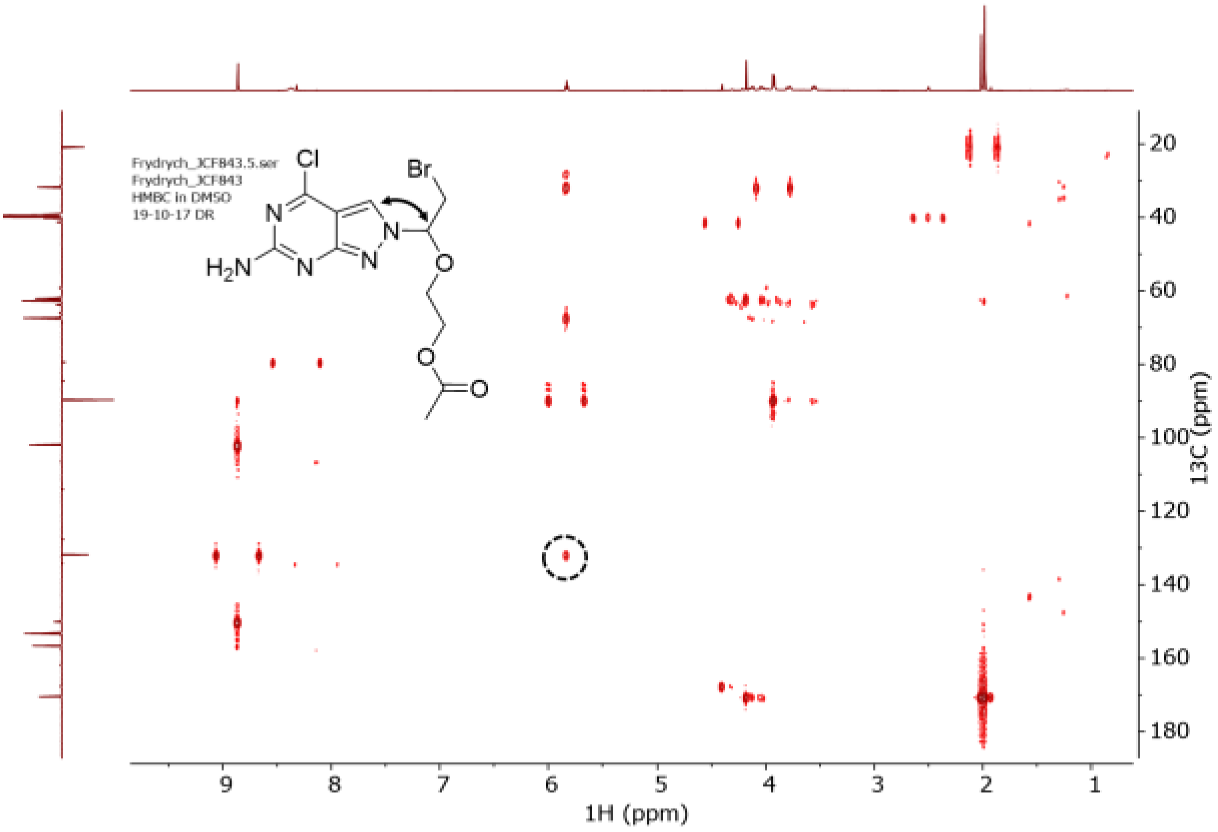

Figure S1. HMBC spectrum of compound **22** showing a three-bond correlation crucial for the stereochemical assignment.

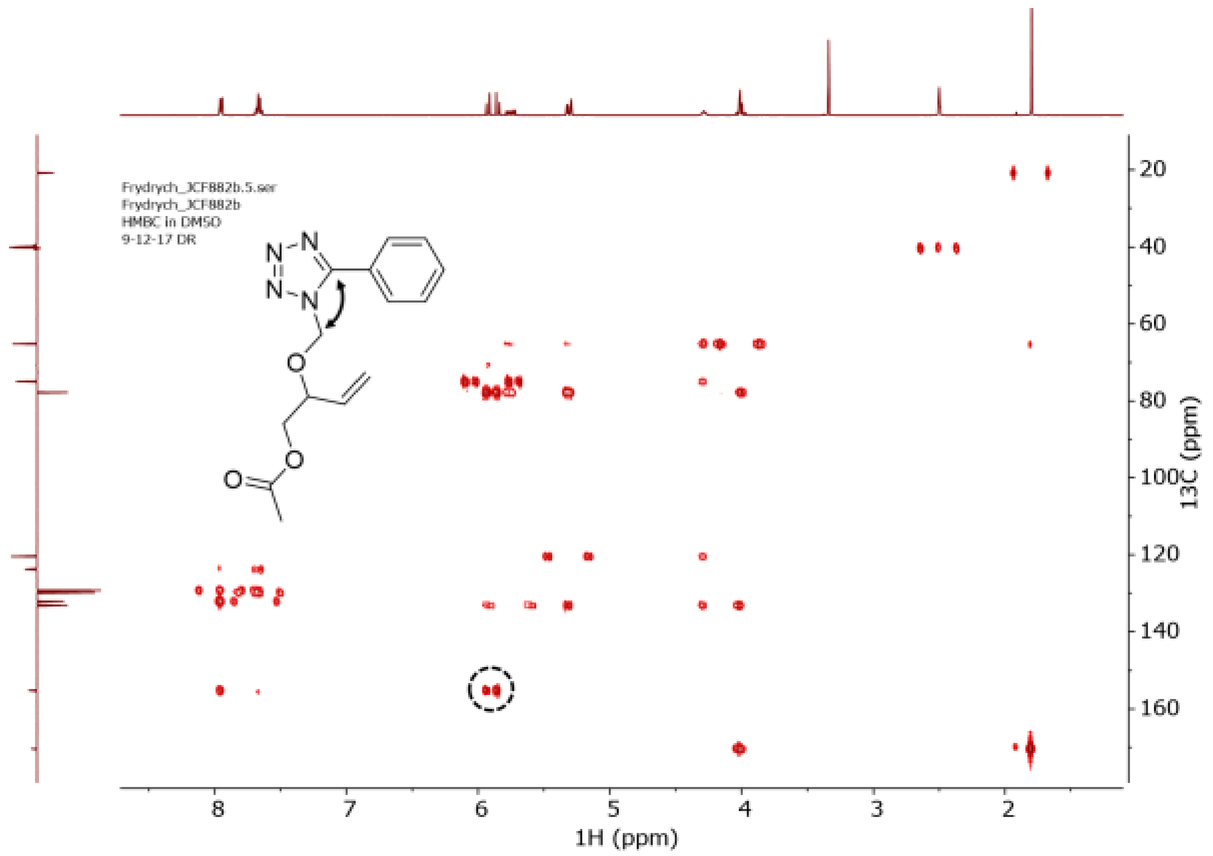

Figure S2. HMBC spectrum of compound **42** showing a three-bond correlation crucial for the stereochemical assignment.
